# The development and use of a molecular model for soybean maturity groups

**DOI:** 10.1186/s12870-017-1040-4

**Published:** 2017-05-30

**Authors:** Tiffany Langewisch, Julian Lenis, Guo-Liang Jiang, Dechun Wang, Vince Pantalone, Kristin Bilyeu

**Affiliations:** 1 Plant Genetics Research Unit, United States Department of Agriculture-Agricultural Research Service, University of Missouri, 110 Waters Hall, Columbia, MO 65211 USA; 2 Dow AgroSciences LLC, 454 E 300N Road, Gibson City, IL 60936 USA; 3 Agricultural Research Station, Virginia State University, P.O. Box 9061, Petersburg, VA 23806 USA; 4 Department of Plant, Soil, and Microbial Sciences, Michigan State University, Plant and Soil Sciences Building, 1066 Bogue St., Room 348E, East Lansing, MI 48824 USA; 5 Department of Plant Sciences, University of Tennessee, 2431 Joe Johnson Drive, Knoxville, TN 37996 USA

**Keywords:** Maturity group, *E* genes, *Glycine max*, Soybean

## Abstract

**Background:**

Achieving appropriate maturity in a target environment is essential to maximizing crop yield potential. In soybean [*Glycine max* (L.) Merr.], the time to maturity is largely dependent on developmental response to dark periods. Once the critical photoperiod is reached, flowering is initiated and reproductive development proceeds. Therefore, soybean adaptation has been attributed to genetic changes and natural or artificial selection to optimize plant development in specific, narrow latitudinal ranges. In North America, these regions have been classified into twelve maturity groups (MG), with lower MG being shorter season than higher MG. Growing soybean lines not adapted to a particular environment typically results in poor growth and significant yield reductions. The objective of this study was to develop a molecular model for soybean maturity based on the alleles underlying the major maturity loci: *E1*, *E2*, and *E3*.

**Results:**

We determined the allelic variation and diversity of the *E* maturity genes in a large collection of soybean landraces, North American ancestors, Chinese cultivars, North American cultivars or expired Plant Variety Protection lines, and private-company lines. The *E* gene status of accessions in the USDA Soybean Germplasm Collection with SoySNP50K Beadchip data was also predicted. We determined the *E* allelic combinations needed to adapt soybean to different MGs in the United States (US) and discovered a strong signal of selection for *E* genotypes released in North America, particularly the US and Canada.

**Conclusions:**

The *E* gene maturity model proposed will enable plant breeders to more effectively transfer traits into different MGs and increase the overall efficiency of soybean breeding in the US and Canada. The powerful yet simple selection strategy for increasing soybean breeding efficiency can be used alone or to directly enhance genomic prediction/selection schemes. The results also revealed previously unrecognized aspects of artificial selection in soybean imposed by soybean breeders based on geography that highlights the need for plant breeding that is optimized for specific environments.

**Electronic supplementary material:**

The online version of this article (doi:10.1186/s12870-017-1040-4) contains supplementary material, which is available to authorized users.

## Background

In soybean [*Glycine max* (L.) Merr.], the transition from vegetative to reproductive growth is largely dependent on plant responses to relative changes in light and dark periods. Day length and temperature conditions are essential for triggering the onset of flowering, which ultimately affects when the soybean matures. Planting date and environmental factors also play a role in maturity, but photoperiod response is the most crucial for determining when a plant reaches physiological maturity. Since day length varies with latitude, soybean has been adapted to grow in specific latitudinal environments. Optimal growth and yield potential are only achieved when soybean is grown in its region of optimum adaptation. Soybean breeders in the United States (US) developed a classification system where soybean lines are assigned into one of twelve maturity groups (MGs) (00-X) based on their latitudinal adaptation [1, 2]. Early flowering and maturing lines grown in shorter seasons in the northern latitudes have lower MG numbers [1, 2]. The later flowering and maturing lines grown in the southern extended season growing regions have higher maturity numbers [1, 2]. If northern early-maturing lines are grown in southern regions, they will flower sooner, have less vegetative growth, and typically have lower yield than the later-maturing lines. Conversely, if late-maturing lines are grown in northern regions, they often flower late in the growing season and do not mature before a killing frost. Even though a standard maturity classification system was established and the maturity of soybean lines is compared to a known maturity check, soybean maturities can vary among years and similar latitudinal locations because of different planting dates, relative maturity scoring, season lengths, temperature, and other environmental factors such as rainfall [2].

In addition to environmental cues, maturity is determined by maturity genes and the allelic variation of these genes. Previous maturity studies identified nine maturity loci: *E1* [3], *E2* [3], *E3* [4], *E4* [5], *E5* [6], *E6* [7], *E7* [8], *E8* [9], and *E9* [10]. In the *E* series, the dominant version of the gene confers later flowering and later maturity except for *E6* and *E9,* where the dominant alleles have an early-flowering phenotype [7, 10]. In recent years, *E1* [11], *E2* [12], *E3* [13], *E4* [14], and *E9* [15] have been molecularly characterized. *E1* is a novel legume-specific transcription factor that is distantly related to the B3 superfamily [11]. The *E1* allele is functional, *e1-as* is not fully functional, and both *e1-fs* and *e1-nl* are nonfunctional [11]. The *e1-as* allele has the missense mutation R15T [11]. The *e1-fs* allele is a frameshift mutation caused by a single-base deletion, and *e1-nl* is the deletion of the entire *E1* gene [11]. *E2* (*GmGIa*) is suggested to be involved in the circadian rhythm and flowering time pathway due to its homology to Arabidopsis GIGANTEA [12]. The *E2* allele is functional, and the nonfunctional *e2* allele has a T1561A single nucleotide polymorphism (SNP) causing a K521* nonsense mutation in exon 10 [12]. The *e2* allele also has an early-flowering phenotype, but the *E1* locus has a larger effect on flowering than the *E2* locus [16, 17]. Both *E3* (*GmPhyA3*) and *E4* (*GmPhyA2*) are phytochrome A genes that respond to different red-to-far-red light ratios under long-day conditions [18]. *E3-Ha* and *E3-Mi* are both functional alleles, but *E3-Mi* has a 2633 bp region deleted from the third intron [13]. The three nonfunctional alleles, *e3-tr*, *e3-ns*, and *e3-fs*, have mutations that produce truncated proteins [13, 19]. The *e3-tr* allele is missing the last exon because of a 13.33-kb genomic deletion [13]. The *e3-ns* allele has a C3139T nonsense mutation in exon three, and *e3-fs* has a single-base insertion in exon one that causes a frameshift [19]. Another nonfunctional allele, *e3-Mo,* has a G1050R missense mutation [13]. *E4* has a functional allele and five nonfunctional alleles [14, 20]. The nonfunctional *e4* (SORE-1) allele has a 6238 bp Ty1/copia-like retrotransposon inserted in the first exon producing a truncated protein with 237 amino acids [14]. The other nonfunctional alleles, *e4-oto*, *e4-tsu*, *e4-kam*, and *e4-kes,* have single base-pair deletions that result in truncated proteins with 456, 759, 894, and 979 amino acids, respectively [14, 20].

Studies have been conducted in China and Japan to examine how allelic variation of these maturity genes affects flowering time and maturity in different geographic locations. Zhai et al. scored flowering time and maturity and genotyped *E1*-*E4* for 180 cultivars that were grown in three northern and three southern locations in China in 2011 and 2012 [21]. They identified eight genotypic classes of different allele combinations where the earliest-maturing lines had genotypes with *e1-fs*, *e1-nl*, or *e1-as e2 e3-tr e4* and the later-maturing lines were *E1 E2 E3 E4* [21]. The nonfunctional *e1* and *e3-tr* alleles were detected in 38% and 33% of the lines, respectively [21]. The nonfunctional *e4* alleles were rare at a presence rate of 7% [21]. The nonfunctional *e2* allele was prevalent over *E2* at 84%, which was in contrast to the other allele distributions [21]. In addition to developing molecular markers to identify maturity alleles, Tsubokura et al. genotyped *E1*-*E4* and recorded flowering data for 63 landraces, cultivars, and experimental lines that were distributed across nine ecological types in Japan [17]. The early-flowering lines had two or three recessive alleles, and the late-flowering lines were *E1 E2 E3 E4* [17]. The distribution of functional and nonfunctional alleles was similar to that found by Zhai et al. [21], with *e1* at 27%, *e3-tr* at 41%, and *e4* at 14% as well as the prominence of the e2 allele at 84% [17]. This research also suggested that the allelic combinations of the *E* genes may explain 62–66% of flowering variation [17].

However, no comprehensive study has been conducted to establish the relationship between allelic variation of the major maturity genes and flowering and maturity in North America. Our work characterized allelic variation and distribution of *E1*, *E2*, and *E3* for a large collection of soybean lines from different geographic locations with particular focus on soybean accessions developed and released in the US. Most importantly, we applied this knowledge to create a molecular maturity model using the most prominent *E* allelic combinations for different MGs in the US. This maturity model can be applied towards improving target breeding and trait introgression for various production environments.

## Results

### *E1* and *E2* selection differs between North American and Chinese cultivars

To understand maturity gene allelic diversity and selection, we determined *E1*, *E2*, and *E3* genotypes for 238 soybean accessions in different categories, including 127 landraces (Additional file 1: Table S1), 48 Chinese cultivars (Additional file 1: Table S2), 17 North American ancestors (Table 1), and 46 North American cultivars (Additional file 1: Table S3). We directly genotyped *E1/e1-as*, *E2/e2*, and *E3-Ha/E3-Mi/e3-tr* in 17 North American ancestors, 52 landraces, and 25 North American elite cultivars selected from those lines. For the remaining lines taken from the Zhou’s 302 resequencing SNP dataset [22], *E1* and *E2* were identified by their causative SNPs, and *E3* was classified by visualizing the *E3* chromosomal region with SNPViz [23]. We also genotyped *E4* and *e4 (SORE-1)* in the North American ancestors (Table 1). Since all the ancestor lines were *E4,* and the nonfunctional *e4* alleles are rare and have only been found in a small northern region in Japan [20], we elected not to include *E4* in the remainder of this study.

**Table 1:** Genotypes of major maturity genes of the North American ancestors

PI Number	Name	MG	*E1/e1-as*	*E2/e2*	*E3/e3-tr*	*E4/e4 (SORE-1)*
PI 548382	Manitoba Brown	00	*E1*	***e2***	***e3-tr***	*E4*
PI 548311	Capital	0	***e1-as***	*E2*	***e3-tr***	*E4*
PI 548379	Mandarin (Ottawa)	0	***e1-as***	***e2***	***e3-tr***	*E4*
PI 548391	Mukden	II	*E1*	***e2***	*E3-Ha*	*E4*
PI 548406	Richland	II	***e1-as***	***e2***	***e3-tr***	*E4*
PI 548298	A.K. (Harrow)	III	*E1*	*E2*	*E3-Ha*	*E4*
PI 548318	Dunfield	III	*E1*	***e2***	*E3-Ha*	*E4*
PI 548348	Illini	III	*E1*	*E2*	*E3-Ha*	*E4*
PI 548362	Lincoln	III	***e1-as***	*E2*	*E3-Ha*	*E4*
PI 548603	Perry	IV	***e1-as***	*E2*	*E3-Mi*	*E4*
FC 33243	Anderson	IV	*E1*	*E2*	*E3-Ha*	*E4*
PI 548488	S-100	V	*E1*	*E2*	*E3-Ha*	*E4*
PI 548456	Haberlandt	VI	*E1*	*E2*	***e3-tr***	*E4*
PI 548477	Ogden	VI	*E1*	*E2*	*E3-Ha*	*E4*
PI 548445	CNS	VII	*E1*	*E2*	*E3-Mi*	*E4*
PI 548485	Roanoke	VII	*E1*	*E2*	*E3-Ha*	*E4*
PI 548657	Jackson	VII	*E1*	*E2*	*E3-Ha*	*E4*

Bold allele names indicate the recessive early alleles

About 90% of the landraces and a significant portion of the Chinese cultivars and North American ancestors were *E1* (Table 1). Interestingly, *e1-as* was prevalent for over 70% of the North American cultivars, suggesting artificial selection of *e1-as* from the North American ancestors (Table 1). The *e2* allele was predominant in the landraces and Chinese cultivars, but 71% of the North American ancestors were *E2* (Table 2). Despite the *E2* preference in the North American ancestors, the *E2* and *e2* alleles were evenly distributed in the North American cultivars (Table 2). The prevalence of *E3* in the landraces was maintained in the Chinese cultivars as well as in the North American ancestors and, subsequently, in the North American cultivars (Table 2).

**Table 2:** Allelic variation of *E1*, *E2*, and *E3* for landraces, Chinese cultivars, North American ancestors, and US cultivars

	Landraces	Chinese Cultivars	North American Ancestors	US Cultivars
	%	%	%	%
***e1-nl***	-	-	-	2
***e1-as***	9	29	29	**72**
*E1*	**91**	**71**	**71**	26
***e2***	**60**	**77**	29	43
*E2*	40	23	**71**	**57**
***e3-tr***	17	17	29	26
*E3*	**83**	**83**	**71**	**74**
*N*	*127*	*48*	*17*	*46*

Bold allele names indicate the recessive early alleles. Bold percentage values represent the more frequent alleles at each locus

The landraces, Chinese cultivars, North American ancestors, and North American cultivars can be grouped into *E* genotype groups that are defined as a single genotype representing the combined allele status of *E1*, *E2*, and *E3*. These lines were distributed among several *E* genotype groups, but most of the lines from each category belonged to either one or two *E* genotype groups. Eight main *E* genotype groups were identified, but other genotype groups, such as *e1-nl e2 E3*, were possible when rare alleles are present. Seventy-seven percent of the landraces were *E1 e2 E3* or *E1 E2 E3* (Table 3). Half of the Chinese cultivars were also *E1 e2 E3*, but the remaining lines were distributed from 4 to 15% in five other genotype groups (Table 3). The North American ancestors also maintain this trend with 46% of lines being *E1 E2 E3*. In contrast, three-fourths of the North American cultivars had an *e1-as* genotype, and 22% and 28% of them are *e1-as e2 E3* or *e1-as E2 E3*, respectively (Table 3).

**Table 3:** Distribution of *E* genotypes in landraces, Chinese cultivars, North American ancestors, and US cultivars

	Landraces	Chinese Cultivars	North American Ancestors	US Cultivars
	%	%	%	%
*e1-nl e2 E3*	–	–	–	2
*e1-as e2 e3-tr*	2	4	12	11
*e1-as e2 E3*	4	10	–	22
*e1-as E2 e3-tr*	2	–	6	11
*e1-as E2 E3*	2	15	12	**28**
*E1 e2 e3-tr*	9	13	6	4
*E1 e2 E3*	**44**	**50**	12	4
*E1 E2 e3-tr*	4	–	6	–
*E1 E2 E3*	33	8	**46**	17

Bold values represent the most frequent allele combinations

### Predicted *E* Genotypes of the USDA soybean germplasm collection reveal that multiple *E* allele combinations belong in each MG

Examining *E1*, *E2*, and *E3* allelic diversity and variation among landraces, North American ancestors, and cultivated lines from China and North America provided a snapshot of *E* allele distribution and selection. However, the analysis was not a completely comprehensive view of *E* gene variation and only represented 1% of the available *Glycine max* accessions. The USDA Soybean Germplasm Collection houses 21,729 Glycine accessions, including over 19,000 *Glycine max*. The *Glycine max* accessions are from over 90 countries with 77% of lines originating in China. Seed and phenotypic data for this collection is available at the Germplasm Resources Information Network (GRIN, http://www.ars-grin.gov/npgs/) of the National Plant Germplasm System. Since genotyping *E1*, *E2*, and *E3* for all of the *Glycine max* accessions was not feasible, we used the publicly-available SoySNP50K genotypes for this collection to predict the *E* alleles based on SNPs that were highly significantly associated with the predominant alleles of each *E* gene [24]. *E1/e1-as* and *E2/e2* were each predicted using one associated SNP, and two markers were identified and used to predict *E3/e3-tr* (see Methods). The *E* gene alleles for these three maturity genes were predicted for 17,762 *Glycine max* accessions with available SoySNP50K data.

The soybean accessions in the germplasm collection have been characterized for agronomic phenotypes in the US for MG regardless of origin. For the *Glycine max* accessions analyzed here, the majority are adapted to mid-latitude ranges, and only 9.6% (MGs 000–0) and 14.5% (MGs VII-X) are adapted to the extreme northern and southern latitudes (Table 4). The predicted functional *E1* allele was more common in middle and late MGs (MGs III-X) than predicted *e1-as* (Table 4). The predicted *e1-as* allele was prevalent in the early MGs 0-I but not for MG 000 (Table 4). Our predictions did not include the nonfunctional *e1-nl* or *el-fs* alleles [11]. The predicted nonfunctional *e2* allele was predominant across all MGs (Table 4). All MGs had a high frequency of predicted *E3* except for the very early MGs 000-I, which had a mostly equal distribution of *E3* and *e3-tr* (Table 4). For this analysis, no distinction was made between *E3-Ha* and *E3-Mi*, and only functional *E3* and nonfunctional *e3-tr* were predicted. Both *e1-as* and *e3-tr* were prevalent in early-maturing lines and the functional *E1* and *E3* in later maturing lines. In general, lines from later MGs were more likely to have the predicted functional alleles *E1* and *E3* than the nonfunctional *e1-as* or *e3-tr*. The exception to this trend was the high prediction for *E1* in MG 000, but the predictions did not include the null alleles of *E1* (*e1-nl* and *e1-fs*, noted herein as e1-n*), which can be present in early-maturing lines.

**Table 4:** Allelic variation of predicted *E1*, *E2*, and *E3* for *Glycine max* accessions from the USDA Soybean Germplasm Collection

	MG 000	MG 00	MG 0	MG I	MG II	MG III	MG IV	MG V	MG VI	MG VII	MG VIII	MG IX	MG X
	%	%	%	%	%	%	%	%	%	%	%	%	%
***e1-as***	30	**55**	**70**	**62**	48	31	12	1	3	4	4	2	1
*E1*	**70**	45	30	38	**52**	**69**	**88**	**99**	**97**	**96**	**96**	**98**	**99**
***e2***	**97**	**96**	**94**	**83**	**72**	**66**	**82**	**86**	**80**	**70**	**85**	**89**	**91**
*E2*	3	4	6	17	28	34	18	14	20	30	15	11	9
***e3-tr***	**61**	**51**	**52**	43	29	35	27	33	17	10	9	8	5
*E3*	39	49	48	**57**	**71**	**65**	**73**	**67**	**83**	**90**	**91**	**92**	**95**
*N*	*138*	*495*	*1065*	*1605*	*1973*	*1900*	*4111*	*2436*	*1458*	*885*	*899*	*692*	*105*

Bold allele names indicate the recessive early alleles. Bold percentage values represent the more frequent alleles at each locus

When evaluating the soybean germplasm collection of *E* predictions *en masse*, we found that multiple *E* genotype combinations were classified within the same MG. The eight most common *E* genotype groups were distributed throughout the MGs. Early MGs are typically *e1-as e2 E3/e3-tr*, and later MGs are mostly *E1 e2 E3* (Table 5). However, accessions with different *E* genotypes can be classified into the same MG. The eight *E* genotype groups were not evenly distributed within a MG or across MGs. For example, 50% or more of the lines from the early MGs 00-III consisted of two to three more prevalent *E* genotypes (Table 5). The later MGs V-IX consistently had only one *E* genotype, *E1 e2 E3*, that comprised more than 50% of the lines within those MGs (Table 5). Ultimately, lines classified in the same MG can have more than one *E* genotype. Comparable results were also observed by Zhai et al. who reported that similar flowering phenotypes could have different *E* allele combinations [21].

**Table 5:** Distribution of predicted *E* genotypes for *Glycine max* accessions from the USDA Soybean Germplasm Collection

	MG 000	MG 00	MG 0	MG I	MG II	MG III	MG IV	MG V	MG VI	MG VII	MG VIII	MG IX	MG X
	%	%	%	%	%	%	%	%	%	%	%	%	%
*e1-as e2 e3-tr*	17	**30**	**39**	19	2	1	0.63	0.45	2	3	3	0.72	0.95
*e1-as e2 E3*	11	**22**	**26**	**28**	**22**	4	1	0.33	0.48	0.90	0.56	1	–
*e1-as E2 e3-tr*	–	0.40	2	7	8	3	0.32	0.16	0.14	–	–	–	–
*e1-as E2 E3*	3	3	2	9	15	**23**	10	0.45	–	–	0.78	–	–
*E1 e2 e3-tr*	**44**	**20**	10	16	17	**29**	24	30	13	5	5	6	4
*E1 e2 E3*	25	**23**	19	19	**30**	**32**	**56**	**55**	**64**	**61**	**76**	**81**	**87**
*E1 E2 e3-tr*	–	0.81	0.38	1	1	2	2	2	1	2	0.67	0.58	–
*E1 E2 E3*	–	0.20	0.66	1	4	7	6	11	19	28	14	10	9
*N*	*138*	*495*	*1065*	*1605*	*1973*	*1900*	*4111*	*2436*	*1458*	*885*	*899*	*692*	*105*

Bold values represent the values of frequencies greater than or equal to 20%

### Geographic location influences the distribution of predicted *E* Genotypes of the USDA soybean germplasm collection

**Fig. 1:**
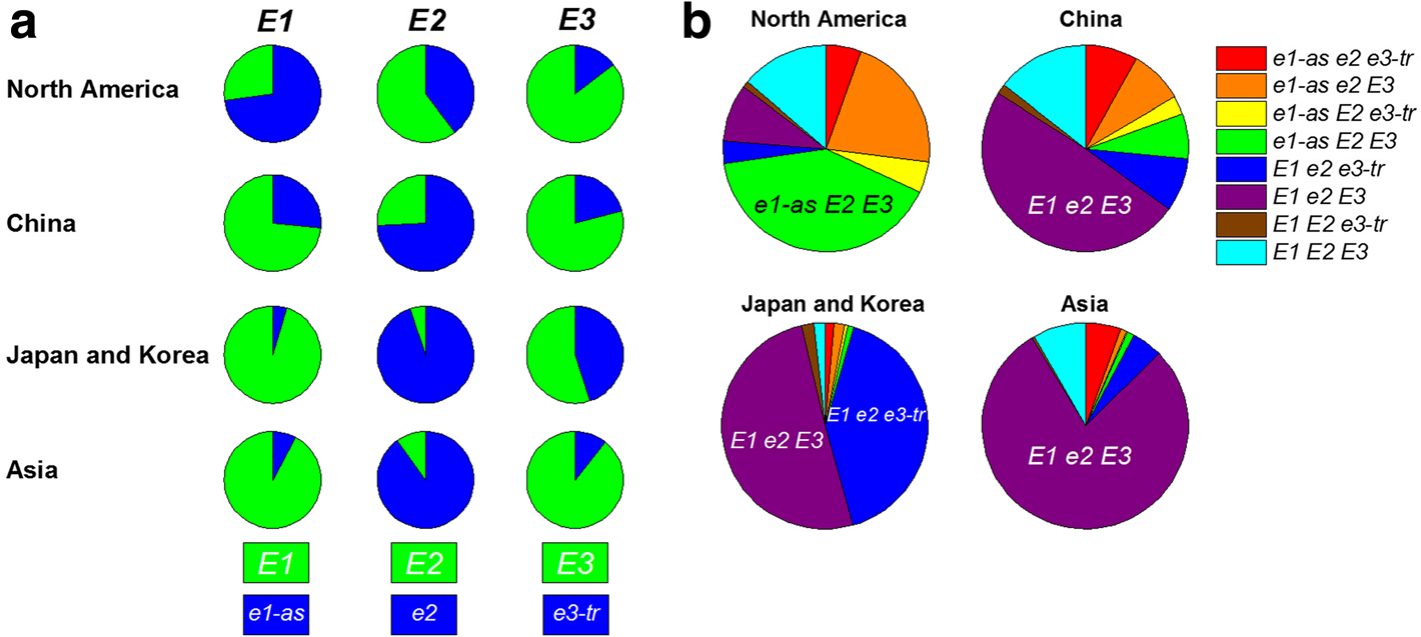
Distribution of predicted *E* alleles and genotypes for *Glycine max* accessions from the USDA Soybean Germplasm Collection by country or region of origin. **a** The allele frequencies of predicted *E1*, *E2*, and *E3* are shown for four geographical locations—North America, China, Japan and Korea, and Asia (16 Asian countries excluding China, Japan, and Korea are referred to as Asia with 90% of the soybean lines originating in Vietnam, Indonesia, India, and Nepal). Japan and Korea data were combined because their distributions of predicted *E* alleles were indistinguishable). The functional *E1*, *E2*, and *E3* alleles are *green*, and *e1-as* and the nonfunctional alleles, *e2* and *e3-tr*, are *blue*. **b** Eight predicted *E* genotype frequencies are grouped by geographic location

Recent genomic studies have provided insight into the genetic architecture and population structure of soybean, particularly as it relates to the development of cultivars for North American environments from a very limited set of ancestral lines [25]. Both ADMIXTURE [26] and principal components analysis (PCA) have painted a picture of distinct population structures for various soybean accessions divided by country of origin [25]. We investigated the predicted *E* genotypes from the accessions in the USDA Soybean Germplasm Collection in the context of country of origin to determine if *E* genotypes were characteristic for different geographies. Nearly 88% of the soybean germplasm collection originated in China, Japan, Korea, a group of other Asian countries, and the US. For this study, 16 Asian countries, excluding China, Japan, and Korea are referred to as Asia with 90% of their soybean lines originating in Vietnam, Indonesia, India, and Nepal. Accessions from China and the combined group of Japan and Korea each accounted for over 30% of the collection, while lines from North America and Asia each comprised about 10% of the collection. Over 90% of the Japanese, Korean, and Asian soybean accessions were predicted to be functional *E1* and nonfunctional *e2* (Fig. 1a). Chinese lines were also mostly predicted as *E1* and *e2* but to a lesser extent than the other Eastern Hemisphere locations. Only lines developed in North America had a majority of the accessions predicted as *e1-as* and *E2,* which is in contrast to the other geographic regions. However, predicted functional *E3* was predominant in all locations, except that the predicted *E3* and *e3-tr* were almost evenly distributed in Japanese and Korean accessions.

Even though each of the eight *E* genotype groups was represented in all geographic locations, only three genotypes—*e1-as E2 E3*, *E1 e2 e3-tr*, and *E1 e2 E3*—were predominant (Fig. 1b). In North America, more than 40% of lines were *e1-as E2 E3*. The genotypic groups *e1-as e2 E3* and *E1 E2 E3* comprised 22 and 14% of lines, respectively in North America. Nearly half of the Chinese accessions were predicted to be *E1 e2 E3* with an additional 15% of lines predicted as *E1 E2 E3*. Accessions from Japan and Korea were primarily *E1 e2 e3-tr* or *E1 e2 E3* with relatively few lines categorized in the other E genotype groups. Over three-fourths of Asian lines were predicted to be *E1 e2 E3*. Asia has a large number of late-maturing lines from MG V and greater compared to other regions. Even though few Asia accessions were from the early MGs 0-III, 5% of lines were predicted to be *e1-as e2 e3-tr*. Although a stark contrast of predicted *E* allele and genotype distributions was evident between soybean accessions from North America and the other regions examined, China generally had the most variation of *E1* and *E2* alleles as well as *E* genotype groups compared to the other Eastern Hemisphere locations, which are remarkably similar to one another with the exception of *E3*.

### Allelic variation of *E* genes contributes to MG classification

Since achieving appropriate maturity when developing cultivars is essential for public and private breeders, we explored the correlation of *E* gene variation within defined MGs for established high-yielding US cultivars. Private-company breeding programs are largely responsible for commercial variety development and the release of new cultivars in the US. These cultivars are always intellectual property and often were protected from unauthorized use by the US Plant Cultivar Protection (PVP) Act. Since the legal protection of PVP lines expires after 20 years, many of these lines are now released for public use. We completed a large-scale direct genotyping survey of *E1*, *E2*, and *E3* for 651 expired PVP (ex-PVP) lines from MGs 00-IX (Additional file 1: Table S4) and 123 more modern private-company well characterized US cultivars and experimental lines (Additional file 1: Table S5).

**Fig. 2.**
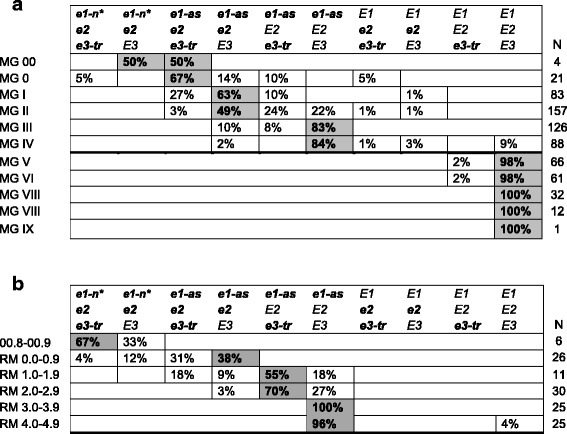
Classification of *E* genotype groups by MG based on ex-PVP and private-company soybean lines. Reading across each row, the frequency of the *E* genotype is shown as a percentage of the total number of lines with that *E* genotype compared to all lines examined within a MG. For example, for MG III, 83% of the 126 lines had the *e1-as E2 E3* genotype while 10% had *e1-as e2 E3*, and 8% had *e1-as E2 e3-tr.* The *E* genotypes with the highest percentage in each MG are bolded and highlighted gray. The alleles *e1-nl* and *e1-fs* are combined as e1-n*. **a** The *E* genotypes for the ex-PVP lines are grouped into MG 0-IX. **b** The *E* genotypes from the private-company lines provided by Dow AgroSciences are arranged by RM. Two *E* genotype groups were excluded because they only occurred with about 10% frequency in RM 0.0–0.9 (e1-n* E2 e3-tr and e1-n* E2 E3).

Very few ex-PVP cultivars were adapted to the extreme northern or southern latitudinal regions, and the majority of cultivars were MG I-IV. These cultivars were divided into ten *E* genotype groups, but 98% of the lines belonged to one of five categories—*e1-as e2 e3-tr*, *e1-as e2 E3*, *e1-as E2 e3-tr*, *e1-as E2 E3*, and *E1 E2 E3* (Fig. 2a). Ignoring the rare genotypes, the earliest MGs were *e1-as e2* e3-*tr*. MG I and II were largely *e1-as e2 E3*. MGs III and IV were almost exclusively *e1-as E2 E3*. MG V and later were almost entirely completely functional *E1 E2 E3*. Even though many cultivars within a specific MG shared a predominant *E* genotype, different *E* genotypes were found in each MG, especially for MGs I and II (Fig. 2a). For example, 49% of MG II was *e1-as e2 E3*, but 24% and 22% were *e1-as E2 e3-tr* and *e1-as E2 E3*, respectively (Fig. 2a). The identification of *E* genotypes based on variation of *E* alleles from cultivars from MGs 0-IX revealed that the very early-maturing lines were nonfunctional *e1 e2 e3-tr*, and late-maturing lines were *E1 E2 E3*, with a distinct separation occurring between MG IV and V for *e1-as* and *E1*, respectively. However, multiple *E* genotypes could be found in a single MG.

We directly genotyped *E1*, *E2*, and *E3* in 123 high-yield private cultivars with stringent maturity scoring criteria that included testing in multiple US locations and years (Additional file 1: Table S5). These cultivars from a single company were scored for relative maturity (RM) using days to maturity and comparing those values with checks of known RM, and they ranged from RM 00.8 to 4.9. Nine *E* genotype groups were identified with 70% of the lines being either *e1-as E2 e3-tr* or *e1-as E2 E3* (Fig. 2b). The very early RM lines most often utilized the null alleles of *e2* with either e1-n* or *e1-as* alleles. Fifty-five percent and 70% of RM 1.0–1.9 and RM 2.0–3.9, respectively, were *e1-as E2 e3-tr* (Fig. 2b). The RM 3.0–4.9 lines were all *e1-as E2 E3* with only one exception (Fig. 2b).

The identified *E* genotype groups overlapped between the ex-PVP lines and the private-company lines, but the most frequent *E* genotype within a MG differed for all maturities except MG III and MG IV. The ex-PVP lines were primarily *e1-as e2 E3* for MG I and MG II, but *e1-as E2 e3-tr* was more common in the private-company lines (Fig. 2). Interestingly, all of the private cultivars except one examined between RM 1.0 and 4.9 had *e1-as* alleles, while 71% of the ex-PVP lines had *e1-as*. However, the ex-PVP lines included 172 late-maturing lines (MG V and later). Between MG I and MG IV, 97% of the ex-PVP lines had *e1-as* alleles. The functional *E2* and *E3* alleles were predominant in the ex-PVP and private-company lines. About 69% and 74% of the ex-PVP and private-company lines were *E2*, respectively. Similarly, the majority of ex-PVP lines were *E3*, and about two thirds of the private-company lines were *E3*.

### An *E* gene molecular maturity model for US MGs

Our results provide a selection model to account for the vast majority of variation in soybean flowering time and maturity for major US production environments (MGs 0-V). The sum of the results presented here is the ability to target soybean breeding for different maturities in the US using the genotype of *E1*, *E2*, and *E3*, a concept we term the “molecular maturity model” (Table 6). This model was based mostly on cultivars developed for release in the US. Even though we presented evidence that multiple *E* allele combinations of *E1*, *E2*, and *E3* can be classified into the same MG, typically a MG had one prevalent *E* genotype (Fig. 2). We selected the most common *E* genotypes for MGs 0-V for the model to target US MG with high yield potential.

**Table 6:** Proposed *E* gene maturity model for the US

MG	*E1/e1-as*	*E2/e2*	*E3/e3-tr*
0	***e1-as***	***e2***	***e3-tr***
I	***e1-as***	***e2***	***e3-tr***
I	***e1-as***	***e2***	*E3*
II	***e1-as***	***e2***	*E3*
II	***e1-as***	*E2*	***e3-tr***
III	***e1-as***	*E2*	*E3*
IV	***e1-as***	*E2*	*E3*
V	*E1*	*E2*	*E3*

Bold allele names indicate the recessive early alleles

Generally the early MGs in the North have the missense *e1-as* allele and the *e2* and *e3-tr* nonfunctional alleles. As latitudes decrease from the northern to southern US, functional *E2* and *E3* alleles are more common. However, cultivars from the northern and central US are almost exclusively *e1-as*, and a dramatic shift to the functional *E1* allele occurs for MG V. Nearly all MG V and later lines have the completely functional genotype *E1 E2 E3*.

A use of the model can be to identify the most appropriate *E* genotype for a targeted MG for parent or progeny selection. Targeting maturity groups would be accomplished by selecting the allele combinations for *E1*, *E2*, and *E3* listed for each maturity group in the model. Alternatively, predicting the MG for experimental lines with characterized *E* genotypes is also possible. Soybean genomic selection strategies should explore the utility of using the *E* genotypes as fixed effects.

## Discussion

Natural and artificial selection are forces of crop evolution and improvement that act upon genetic variants that arise or are present in ancestral populations. Identifying signals of selection is an important step to develop knowledge that can be used to target crop improvement through selective breeding. Wild soybeans (*Glycine soja* Sieb. et Zucc) can be found from Southern China as well as throughout East Asia, and as far north as the Russian Far East (latitudes 24^o^ to 53^o^ North). Domesticated soybean has a historical range that extends north and south from central China possibly centered at the Huang-Huai Valley [27]. Wild soybeans expanding into a wide range of latitudes while maintaining photoperiod sensitivity implies strong natural selection for variant alleles of maturity genes. Early artificial selections by soybean farmers may have reflected this broad geographical range and matched maturity allele variants with the local growing season and environment as domestic soybean cultivation spread throughout East Asia. Modern soybean breeding in the US and Canada that started with a very limited set of ancestral, mostly Chinese, landraces led to artificial selection of a few critical alleles of the major maturity loci *E1*, *E2*, and *E3*. Variants in these three genes were shown here to be responsible for the majority of high-yielding soybean adaptation to US production zones based on daylength related to latitude. A severe genetic bottleneck resulted from the nearly exclusionary use of the missense *e1-as* allele for US MG 0 through IV, accounting for at least 24 million soybean production hectares per year in the US.

The major outcome of this project was the development of a molecular model for soybean MG in the US based on the alleles underlying the major maturity loci: *E1*, *E2*, and *E3*. Our aim was to understand the consequences of selection of alleles of the major maturity genes from soybean breeding targeted to a new continent with a limited number of founding ancestral lines. In order to make the model, we associated allelic variation and diversity of the *E* maturity genes in a large collection of soybean landraces, Chinese cultivars, North American ancestors, and North American cultivars from expired PVP lines as well as private-company lines. The model specifies the *E* allelic combinations needed to adapt soybean to different MGs in the US. The utility of the *E* gene molecular maturity model is the enhanced ability to transfer traits into different MGs and increase the overall efficiency of targeted breeding for specific MGs. In addition, genomic selection schemes should be evaluated in soybean with the *E* genes as fixed effects. Our *E* gene molecular maturity model can enhance the development of new cultivars with desirable traits through targeted plant breeding for specific US production environments.

Our research revealed a strong signal for artificial selection for the recessive *e1-as* allele in nearly all released cultivars in the US in MG 0-IV. In contrast, cultivars released in the US for MG V and later more closely matched the Eastern Hemisphere soybean landraces and cultivars which had a contrasting strong signal for artificial selection for functional *E1* alleles. This finding is surprising because the majority of Asian-originating soybeans have been classified with MGs IV or earlier when assessed in US production environments. We determined that the typical soybean originating from Asia has *E1* and *e2* alleles. Bernard et al. established that soybean lines with contrasting *E1* and *E2* genes (*E1 e2* versus *e1 E2*) had similar flowering times and very similar maturities of approximately MG III [3]. US soybean cultivars earlier than MG V excluded the *E1 e2* genotype, suggesting a negative impact on yield in the US for the predominant *E* genotype found in all Asian soybeans. This result also suggests that soybean breeding for US MG IV or earlier should include selection for *e1-as* when using Asian-originating soybean germplasm to increase overall genetic diversity or mining for new traits.

Our proposed *E* gene maturity model for the US provides guidance to select the *E1*, *E2*, and *E3* genotype to target for each of six major MGs (0-V) grown in the US based on our evaluation of approximately 800 US soybean cultivars. In general, earlier maturing soybeans require more recessive nonfunctional *E* genes with *E1* having a greater impact on MG than *E2*, and *E2* having a greater effect than *E3*. We understand that this maturity model is not perfect, and other maturity genes are likely to be causing additional subtle maturity effects. However, we have been able to use the *E* gene maturity model in our own targeted breeding efforts, and our preliminary results have demonstrated the general effectiveness of the model using just *E1*, *E2*, and *E3*. While the sample sizes were quite small, the extreme northern North American environments (MG 00 and 0) showed some evidence of artificial selection for *e2* alleles and utilization of null alleles of *E1*. The combined effects of very long photoperiods and a relatively short frost-free production season in the extreme northern environments are likely key factors in high frequency utilization of the selected alleles.

Our results generally correspond with previous reports by both Tsubokura et al. [17] and Zhai et al. [21] that characterized the *E1*, *E2*, and *E3* genotypes in different soybean accessions. However, our study included a much wider comprehensive approach with larger sample sizes and included a focus on not only geographical distribution between China and the US but also compared landraces, ancestors, and cultivars of soybeans. We explored the selection patterns of *E1* and *E2* between Chinese and US cultivars and documented the contrasting *E* genotypes between Asian soybeans and US released soybeans for MG IV and earlier.

Recent studies have also shed light on the signatures of selection for North American soybeans, including selection for adaptation to different latitudes. Vaughn and Li concluded that maturity defined population structure when investigating North American soybean populations [28]. One intriguing aspect of that work was an analysis based on the age of cultivar release (prior to 1970 and since 2000) that indicated shifts in selection over time, fixing some regions of the genome while other regions had high diversity within different MGs. We investigated the *E* genotype allele frequencies by decade of release and MG and also saw some evidence for selection over time for early MGs, though the sample size was very small (MG II shifted from 58% *e1-as e2 E3* and 15% *e1-as E2 e3-tr* in the 1970–1989 released lines to 30% *e1-as e2 E3* and 39% *e1-as E2 e3-tr* in the 1990–2009 set, for example). We also discovered differences in *E* genotype allele frequencies between the ex-PVP cultivars and the private-company lines, particularly in the early MGs. The private-company lines relied heavily on the *e1-as E2 e3-tr* genotype for RM 1.0–2.9, although we could see a shift in the predominant *E* genotype to *e1-as E2 E3* at about RM 2.5 (Additional file 1: Table S5). Individual breeding programs could be making private allele selections for their own programs. Indeed, when we predicted *E* genotypes for the Wen et al. dataset of 1062 improved early MG US released soybean lines from more than 40 breeding programs (MG I, II, and III), the predicted *e1-as e2 E3* genotype was relatively rare at less than 9% overall despite nearly 80% of the lines classified as MG I or MG II (Additional file 1: Table S6) [29]. Overall, our results indicate that *achieving* appropriate maturity can be accomplished with different *E* genotypes along with contributions from uncharacterized maturity genes, but *targeting* different MG can be enhanced utilizing our *E* gene maturity model.

One feature of our analyses in developing the maturity gene model was the ability to predict the *E1*, *E2*, and *E3* gene allele status for 17,762 soybean accessions from the USDA Soybean Germplasm Collection. We used a modified GWAS to predict with ~88–98% accuracy for the common functional or missense/nonfunctional alleles of *E1*, *E2*, and *E3*. We provided our data to SoyBase for incorporation into their tool of searchable and downloadable GRIN data (http://www.soybase.org/grindata/) [30]. This publicly-available tool allows users to identify the predicted *E1*, *E2*, and *E3* genotype for any of the soybean accessions with available data. The information will be important for selection of breeding parents.

Our analysis revealed but did not explain why multiple *E* allelic combinations can be present in different MGs. Also unanswered is why there has been such strong selection against the *E1 e2* combination in US-released soybean cultivars. Since our three-gene model does not completely explain soybean maturity, we propose that additional genes are either directly or indirectly interacting with the major maturity genes *E1*, *E2*, and *E3* to fine tune photoperiod response for plant development and optimized production potential in different latitudes. These additional genes may help further delineate MGs and provide *E* allelic combinations that can differentiate late and early lines within a single MG. More studies need to be conducted to identify the interactions with these uncharacterized genes and expand the molecular maturity gene model.

## Conclusions

Developmental transitions from vegetative through reproductive stages in plants have long been considered complex traits controlled by both genetic factors and the environment. A network of signaling pathways that controls flowering time and plant maturity is now apparent in many model species and crops. Here we determined that distinct allelic combinations of just three major soybean maturity genes have been artificially selected to maximally adapt soybean to high yielding production environments in the US. Use of our molecular maturity gene model can dramatically increase soybean breeding efficiency by selecting for desired allele combinations of *E1*, *E2*, and *E3*.

## Methods

### Plant material

In this study, 17 North American ancestors (Table 1), 52 landraces (Additional file 1: Table S1), and 25 North American cultivars (Additional file 1: Table S3) from Hyten et al. [31] and 651 ex-PVP lines from MGs 00-IX (Additional file 1: Table S4) were genotyped for *E1*, *E2*, and *E3*. Seeds for these lines were obtained from GRIN (https://npgsweb.ars-grin.gov/gringlobal/search.aspx?). MG classifications for these lines were taken directly from GRIN; however, many lines have self-reported MGs. Dow AgroSciences provided DNA and RM scores for 123 high-yielding lines roughly equivalent to MGs 00, 0, I, II, III, and IV (Additional file 1: Table S5). The RM scores were determined by estimating the average maturity date from multi-location trials and comparing it with checks of known RM. The MG phenotype was obtained from the US National Plant Germplasm System. Either field evaluations (Plant Introductions) or depositor-submitted (PVP) MG classifications were used.

### 302 Resequencing SNP dataset

We determined *E1*, *E2*, and *E3* genotypes for 302 wild and cultivated soybean lines using a publicly-available resequencing dataset from Zhou et al. [22]. This dataset consisted of 62 *Glycine soja* lines, 110 improved cultivars, and 130 landraces and included 16 North American ancestors, 20 elite cultivars, and 36 landraces from Hyten et al. [31]. Twenty-three North American cultivars, 48 Chinese cultivars (Additional file 1: Table S2), and 80 landraces were exclusive to the 302 resequencing dataset. These lines were sequenced with >11× coverage. The 33 billion 100-bp paired-end reads were aligned to the Williams 82 reference genome (Glyma v1.0) [22]. More than 9.7 million SNPs were identified, including the causative SNPs for *E1* (Gm06:20,007,173) and *E2* (Gm10:44,732,850). We used these causative SNPs to identify the *E1* and *E2* alleles for the resequenced lines. Since *E3* does not have a causative SNP, we examined the *E3* haplotype region (Gm19:47,509,802–47,520,760) using SNPViz to distinguish the functional *E3* from *e3-tr* [23]. However, we were not able to confidently differentiate between *E3-Ha* and *E3-Mi*. For our analysis, we excluded the 62 *Glycine soja* lines, 16 lines with missing SNP data for *E1* or *E2*, and a single cultivar from outside the US and China.

### DNA extraction

Genomic DNA from the North American ancestors was extracted using the manufacturer’s protocols for the DNeasy Plant Mini Kit (Qiagen, Valencia, CA). For each sample, 15 soybean seeds were ground into a fine powder using a coffee grinder, and 0.02 g of this ground seed powder was transferred to a micro centrifuge tube. Samples were then processed according to the DNeasy Plant Mini Kit protocol except only one DNA elution was performed at the end of the protocol. The DNA samples were then stored at −20 °C.

Genomic DNA from the ex-PVP lines was extracted with a modified 96-well small-scale SDS/NaCl DNA seed extraction protocol based on Edwards et al. [32]. One seed was placed into each well of a 96-square deep-well plate, and 1000 μL of extraction buffer (500 mM EDTA, 5 M NaCl, 2 M Tris-HCl pH 8, and 20% SDS) was added. The plates were incubated overnight at 70 °C. After adding 100 μL 5 M NaCl, the samples were incubated for 5 min at room temperature and centrifuged for 5 min at 3600 rpm. The supernatant (200 μL) was transferred to a 96-well plate containing 180 μL isopropanol. This plate was vortexed for 15 s and incubated for 25 min at room temperature. After centrifuging the plate for 5 min at 3600 rpm, the isopropanol was decanted. The remaining pellet was washed with 200 μL 70% ethanol, and the plate was centrifuged for 5 min at 3600 rpm. The ethanol was decanted, and the plate was centrifuged for an additional 2 min at 1500 rpm. The pellets were subsequently washed with 70% ethanol a second time and incubated at 80 °C for 90 min. Following the addition 100 μL 250 mM NaCl, the plate was incubated for 60 min at room temperature. DNA was precipitated a second time with 100 μL isopropanol. The plate was vortexed for 10 s and incubated for 25 min at room temperature. After a final ethanol wash and incubation, the pellet was suspended in 100 μL 1xTE. The samples were incubated overnight at 4 °C and gently vortexed. DNA was then diluted by 1:10 and stored at −20 °C.

### *E1* genotyping assay

A SimpleProbe assay was developed to distinguish *E1* and *e1-as* by detecting the causative G/C SNP (Gm06:20,007,173, W82 Glyma v1.0) with a melting curve analysis. PCR primers were designed from the Williams 82 *e1-as* sequence that was described by Xia et al. [11]. The primers E1up1f (5′-ACACTCAAATTAAGCCCTTTCAACC-3′) and E1r1 (5′-TCCTAAAGTTAGAGGCTTCGC-3′) amplified a 171-bp region, which included the G/C SNP. The SimpleProbe oligonucleotide (Fluorescein-SPC-GGTGGATTTCCTCTTCTTTTGACACTGC-Phosphate) was designed to the *E1* sequence on the anti-sense strand using the LightCycler Probe Design software (Roche Applied Science, Indianapolis, IN). PCR reactions were performed in 20 μl and included the DNA template, 0.5 μM forward primer E1up1f, 0.2 μM reverse primer E1r1, 0.2 μM SimpleProbe, buffer (40 mM Tricine-KOH [pH 8.0], 16 mM MgCl_2_, 3.75 μg ml^−1^ BSA), 5% DMSO, 200 μM dNTPs, and 0.2X Titanium Taq polymerase (BD Biosciences, Palo Alto, CA). The PCR reactions were run on the LightCycler 480 real-time PCR instrument (Roche Applied Science, Indianapolis, IN). The reactions were initially denatured at 95 °C for 3 min and then denatured at 95 °C for 20 s, annealed at 60 °C for 20 s, and elongated at 72 °C for 20 s for 45 cycles. After amplification, a melting curve was run at 95 °C for 1 min, 55 °C for 2 min, followed by a temperature increase of 2.2 °C/s to 77 °C with a continuous acquisition, and a cooling step of 40 °C for 30 s. Analysis of the negative first derivative of the melting curve resulted in a characteristic peak for *E1* and *e1-as* at 68 °C and 62 °C, respectively. For e1-n* allele calling, the *e1-nl* alleles produced no fluorescent signal, and *e1-fs* alleles produced a characteristic peak at 65 °C.

### *E2* genotyping assay

For detection of the *E2* and *e2* allele variants, a SimpleProbe assay was created.

The PCR primers E2for (5′-TGCAACCCCACTACAGCCT-3′) and E2Rev (5′-GAGGCAGAGCCAAAGCCTAT-3′) were designed from the Williams 82 sequence as described by Watanabe et al. 2011 [12]. This 222-bp amplicon includes the A/T SNP (Gm10:44,732,850, W82 Glyma v1.0) at base 1561 in exon 10 where a nonsense mutation occurs in the *e2* allele [12]. The SimpleProbe oligonucleotide (Fluorescein-SPC-GGCATGTCTTATGAAAATATTTGCTGC-Phosphate) was designed to the *E2* sequence on the sense strand using the LightCycler Probe Design software (Roche Applied Science, Indianapolis, IN). PCR reactions and melting curve parameters were identical to the *E1* genotyping assay except that the forward E2for and reverse E2Rev primers had a concentration of 0.2 μM and 0.5 μM, respectively. The melting curve from 55 °C to 77 °C distinguished the *E2* peak at 64 °C and the *e2* peak at 60 °C.

### *E3 and E4* genotyping assays

Separate PCR-based assays were designed for genotyping *E3* and *E4*. The *E3* genotyping assay was carried out as described in Langewisch et al. 2014 [23]. The genotyping of *E4* was performed as described by Liu et al. 2008 [14].

### Predicting *E1*, *E2*, and *E3* alleles for wild and cultivated soybean from the USDA soybean germplasm collection

We predicted the *E1*, *E2*, and *E3* alleles for 18,742 lines in the USDA Soybean Germplasm Collection. These accessions were genotyped for 52,051 SNPs with the Ilumina Infinium SoySNP50K Beadchip [24]. We first associated these SNP markers with each of the maturity genes. To identify associated SNPs, we conducted GWAS using a mixed-linear model approach in the program TASSEL 5.0 (Trait Analysis by aSSociation, Evolution, and Linkage) [33]. We downloaded the W82.a2 version of this data from SoyBase (http://www.soybase.org/dlpages/index.php#snp50k) [30]. We used the 41,895 markers on chromosomes 1–20. In lieu of observed phenotype data, we assigned a numerical value to the common alleles for *E1*, *E2*, and *E3* (based on direct genotype analysis or resequencing data). The completely-functional *E1* and *E2* alleles were coded 2, and their variant alleles, *e1-as* and *e2* were coded 1. The nonfunctional *e3-tr* was designated 1. The two functional alleles, *E3-Ha* and *E3-Mi*, were designated 2 and 3, respectively, or were grouped together. For this analysis, rare alleles, such as *e1-fs* and *e1-nl*, were not considered. *E1*, *E2*, and *E3* genotypes were available for 406 accessions with SoySNP50K data either through direct genotyping or identification of the *E1* and *E2* causative alleles from the Zhou 302 resequencing dataset [22]. Although 406 lines had known alleles for *E1* and *E2*, only 295 had known *E3* alleles. This collection of lines included the 17 North American ancestors, 81 landraces, 26 Chinese cultivars, 217 North American cultivars, and 49 *Glycine soja* lines, and 16 unknown classifications. Both the SoySNP50K genotypes and the *E* allele “phenotypes” for the 406 lines were inputted into TASSEL 5.0, and the mixed-linear model analysis included PCA with three covariants and a scaled identity by state kinship matrix to account for population structure and relatedness [33]. Manhattan plots were drawn to visualize the observed *p* values (Additional file 1: Figure S1).

We selected the top associated SNPs for *E1*, *E2*, and *E3* as proxy predictors of the allele status of these maturity genes. For a particular SNP location, the reference genome Williams 82 SNP allele represented the Williams 82 *E* genotype (e.g. *e1-as*, *E2*, or *E3*). The other SNP allele represented the *E* allele variant (e.g. *E1*, *e2*, or *e3-tr*). We tested prediction accuracy by predicting the *E* genotype of accessions with known *E* genotypes and then compared those predictions with the actual genotypes. For *E1*, SNP ss715593832 (A/G) at Gm06: 19,857,928 (W82.a2) had the greatest –log_10_ *p* value at 36.8 (Additional file 1: Figure S1A). This SNP predicted *E1* correctly 97% and *e1-as* at 98%. The *E2* associated SNP with the largest –log_10_ *p* value of 26 correctly predicted *E2* at 77% in *Glycine max* but incorrectly predicted *Glycine soja* lines (Additional file 1: Figure S1B). Even though ss715607475 (C/T) at Gm10:45,269,968 W82.a2 had a lower –log_10_ *p* value of 15.8, it correctly predicted *E2* in *Glycine max* and *Glycine soja* 79% and 83%, respectively. The *e2* allele was predicted correctly at 98% for *Glycine max* lines; however, *Glycine soja* lines were all *E2*. Initially, an *E3* associated SNP, ss715635690 (A/G, Gm19: 47,514,412 W82.a2) with a –log_10_ *p* value of 23, was identified by grouping *E3-Ha* and *E3-Mi* together in the GWAS analysis (Additional file 1: Figure S1C). This SNP successfully distinguished *E3* and *e3-tr*, but *E3-Mi* was miscalled as *e3-tr*. To improve predictability, two SNPs were chosen for predicting *E3* functional and *e3-tr*. A subsequent GWAS analysis was performed defining *E3-Ha* and *E3-Mi* as separate “phenotypes.” This analysis identified ss715635694 (A/C) at Gm19: 47,564,286 W82.a2 with a –log_10_ *p* value of 21.7 (Additional file 1: Figure S1D). When ss715635690 and ss715635694 were combined, *E3-Ha*, *E3-Mi*, and *e3-tr* were predicted 94%, 55%, and 92% correctly, respectively. Due to the relative inaccuracy for predicting *E3-Mi*, we predicted lines as either functional *E3* or *e3-tr*. The *E1*, *E2*, and *E3* allele status for every soybean accession available with SoySNP50K was predicted based on the allele of the associated SNPs: ss715593832 for *E1* or *e1-as*, ss715607475 for *E2* or *e2*, and the combination of ss715635690 and ss715635694 for *E3* or *e3-tr*. *E* genotype predictions for 963 *Glycine soja* and 17,762 *Glycine max* accessions are available at SoyBase (http://www.soybase.org/grindata/) [30]. The data is searchable. On the “grindata” page, users check the boxes next to *E1*, *E2*, and/or *E3* and click next. On the following page, users can identify germplasm based on the predicted *E* genotype data selected or retrieve the predicted *E* genotype data for a list of user-selected germplasm accessions.

## Additional file

Additional file 1: Table S1.
*E* genotypes of major maturity genes of landraces**. Table S2.** *E* genotypes of major maturity genes of Chinese cultivars. **Table S3.** *E* genotypes of major maturity genes in US cultivars. **Table S4.** *E* genotypes of major maturity genes of the ex-PVP collection. **Table S5.** *E* genotypes of major maturity genes of Dow AgroSciences soybean lines. **Table S6.** Allele calls and maturity gene predictions from the Wen, et al. 2015 dataset. **Figure. S1.** Identification of associated SoySNP50K SNPs used to predict the allele status of *E1*, *E2*, and *E3* for *Glycine max* accessions from the USDA Soybean Germplasm Collection. The Manhattan plot *p*-values arranged by chromosome are shown for GWAS that used known *E1*, *E2*, and *E3* alleles as “phenotypes.” *(A) p*-values for *E1*. *(B) p*-values for *E2. (C) p*-values for *E3-Ha* and *E3-Mi* alleles treated as one “phenotype.” *(D) p*-values for *E3-Ha* and *E3-Mi* treated as individual “phenotypes.” (DOCX 498 kb)

## Abbreviations

DTM: Days to maturity
GRIN: Germplasm Resources Information Network
GWAS: Genome-wide association study
MG: Maturity group
PCA: Principal components analysis
PCR: Polymerase chain reaction
PVP: Plant variety protection
RM: Relative maturity
SNP: Single nucleotide polymorphism
TASSEL: Trait Analysis by aSSociation, Evolution, and Linkage
US: United States
