# Supplementary material for: The development and use of a molecular model for soybean maturity groups
Source: BMC Plant Biol. 2017 May 30;17:91. doi: 10.1186/s12870-017-1040-4 (PMC5450301; doi:10.1186/s12870-017-1040-4)
Supplement: Additional file 1: Table S1. — E genotypes of major maturity genes of landraces. Table S2. E genotypes of major maturity genes of Chinese cultivars. Table S3. E genotypes of major maturity genes in US cultivars. Table S4. E genotypes of major maturity genes of the ex-PVP collection. Table S5. E genotypes of major maturity genes of Dow AgroSciences soybean lines. Table S6. Allele calls and maturity gene predictions from the Wen, et al. 2015 dataset. Figure. S1. Identification of associated SoySNP50K SNPs used to predict the allele status of E1, E2, and E3 for Glycine max accessions from the USDA Soybean Germplasm Collection. The Manhattan plot p-values arranged by chromosome are shown for GWAS that used known E1, E2, and E3 alleles as “phenotypes.” (A) p-values for E1. (B) p-values for E2. (C) p-values for E3-Ha and E3-Mi alleles treated as one “phenotype.” (D) p-values for E3-Ha and E3-Mi treated as individual “phenotypes.” (DOCX 498 kb) [file 12870_2017_1040_MOESM1_ESM.docx]

**Additional files**

**Table S1.** *E* genotypes of major maturity genes of landraces

| **PI Number** | **Name** | **MG** | ***E1/e1-as*** | ***E2/e2*** | ***E3/e3-tr*** |
| --- | --- | --- | --- | --- | --- |
| PI 603424 A | Ben di huang dou | 0 | ***e1-as*** | ***e2*** | ***e3-tr*** |
| PI 317336^*^ | Shinsei | 0 | *E1* | ***e2*** | ***e3-tr*** |
| PI 416890^*^ | Gokuwase natsu daizu | 0 | *E1* | ***e2*** | ***e3-tr*** |
| PI 423954 | Shirome | 0 | *E1* | ***e2*** | ***e3-tr*** |
| PI 153262^*^ | Roumanie | 0 | *E1* | ***e2*** | *E3* |
| PI 81775 | 1486 | I | ***e1-as*** | ***e2*** | ***e3-tr*** |
| PI 253658 B^*^ | No. 9 | I | ***e1-as*** | ***e2*** | *E3* |
| PI 467343^*^ | Yan-nong No. 2 | I | ***e1-as*** | ***e2*** | *E3* |
| PI 507355^*^ | Tokei 423 | I | ***e1-as*** | ***e2*** | *E3* |
| PI 603357 | Du Lu Dou | I | ***e1-as*** | *E2* | ***e3-tr*** |
| PI 317334 A^*^ | Kitamishiro | I | *E1* | ***e2*** | ***e3-tr*** |
| PI 407716^*^ | Jin nung No. 3 | I | *E1* | ***e2*** | ***e3-tr*** |
| PI 83945-3^*^ | Dairyu Tsurunoko | I | *E1* | ***e2*** | *E3* |
| PI 548417^*^ | Soysota | I | *E1* | ***e2*** | *E3* |
| PI 603318 | Xiao zhu yao | I | *E1* | ***e2*** | *E3-Ha* |
| PI 89138 | Zontanorukon | II | ***e1-as*** | ***e2*** | *E3-Ha* |
| PI 567481 | Bao ding huang dou | II | ***e1-as*** | *E2* | ***e3-tr*** |
| PI 437944^*^ | VIR 569 | II | ***e1-as*** | *E2* | *E3* |
| PI 398296 | KAS 173-3 | II | *E1* | ***e2*** | ***e3-tr*** |
| PI 603336 | Qing pi si li huang | II | *E1* | ***e2*** | ***e3-tr*** |
| PI 437653^*^ | Er-da-li | II | *E1* | ***e2*** | *E3* |
| PI 80822^*^ | Shiheigai Shirobana | III | ***e1-as*** | ***e2*** | *E3* |
| PI 88479^*^ | Kungchuling Improved No. 77 | II | *E1* | ***e2*** | *E3* |
| PI 567258 | He pi dou | II | *E1* | ***e2*** | *E3-Ha* |
| PI 567293 | Ben di huang dou | II | *E1* | ***e2*** | *E3-Mi* |
| PI 567525 | Cao qing huang dou | II | *E1* | ***e2*** | *E3-Mi* |
| PI 603756 | Bai dou | II | *E1* | ***e2*** | *E3-Mi* |
| PI 603420 | Hei dou | II | *E1* | *E2* | *E3-Ha* |
| PI 84631^*^ | S-56 | III | *E1* | ***e2*** | ***e3-tr*** |
| PI 84987^*^ | Oni Hadaka | III | *E1* | ***e2*** | ***e3-tr*** |
| PI 417398^*^ | Touhou torotou | III | *E1* | ***e2*** | ***e3-tr*** |
| PI 84987 A^*^ | Oni Hadaka | III | *E1* | ***e2*** | *E3* |
| PI 86024^*^ | Daidzuhinshu satei | III | *E1* | ***e2*** | *E3* |
| PI 404182^*^ | Sin i tu li rau | III | *E1* | ***e2*** | *E3* |
| PI 437321^*^ | Dunganscaja 462 | III | *E1* | ***e2*** | *E3* |
| PI 567364 | Ping luo huang da dou | III | *E1* | ***e2*** | *E3-Mi* |
| PI 567700 | Fu yang (19) | III | *E1* | ***e2*** | *E3-Mi* |
| PI 594451 | Liu yue bao | III | *E1* | ***e2*** | *E3-Mi* |
| PI 603384 | Ping ding xiang | III | *E1* | ***e2*** | *E3-Mi* |
| PI 603596 | Bai hua gu tian dou | III | *E1* | ***e2*** | *E3-Mi* |
| PI 603675 | Huai yin gua dou jia | III | *E1* | ***e2*** | *E3-Ha* |
| PI 157421^*^ | Ebony | III | *E1* | *E2* | ***e3-tr*** |
| PI 407849 | KAS 510-1 | III | *E1* | *E2* | ***e3-tr*** |
| PI 399043 | KLS 903 | III | *E1* | *E2* | *E3-Mi* |
| PI 437654^*^ | Er-hej-jan | III | *E1* | *E2* | *E3* |
| PI 80837^*^ | Mejiro | IV | *E1* | ***e2*** | ***e3-tr*** |
| PI 243541^*^ | Shakujo | IV | *E1* | ***e2*** | *E3* |
| PI 416971^*^ | Kaifuu gyuumou ou 1 | IV | *E1* | ***e2*** | *E3* |
| PI 567189 A^*^ | Ekhabac | IV | *E1* | ***e2*** | *E3* |
| PI 567368 | Xi he huang dou | IV | *E1* | ***e2*** | *E3-Mi* |
| PI 567395 | Lai wa dou | IV | *E1* | ***e2*** | *E3-Mi* |
| PI 567503 | Niu mao huang | IV | *E1* | ***e2*** | *E3-Mi* |
| PI 594615 | Liu yue zao | IV | *E1* | ***e2*** | *E3-Mi* |
| PI 594777 | Liu yue huang | IV | *E1* | ***e2*** | *E3-Ha* |
| PI 602991 | Niu jiao qi da hei dou | IV | *E1* | ***e2*** | *E3-Ha* |
| PI 339734^*^ |  | IV | *E1* | *E2* | ***e3-tr*** |
| PI 437679^*^ | Nan-cou | IV | *E1* | *E2* | *E3* |
| PI 438498^*^ | Sable | IV | *E1* | *E2* | *E3* |
| PI 548342^*^ | Higan | IV | *E1* | *E2* | *E3* |
| PI 548402^*^ | Peking | IV | *E1* | *E2* | *E3* |
| PI 196166^*^ | No. 2296 | V | *E1* | ***e2*** | *E3* |
| PI 567298 | Chan yao dou | V | *E1* | ***e2*** | *E3-Ha* |
| PI 587752 | Xian ning dong huang dou jia | V | *E1* | ***e2*** | *E3-Mi* |
| PI 587848^*^ | Wu chang hei dong dou | V | *E1* | ***e2*** | *E3* |
| PI 588053 A | Xiao li huang | V | *E1* | ***e2*** | *E3-Mi* |
| PI 594579 | Zhong he tian cheng dou | V | *E1* | *E2* | *E3-Mi* |
| PI 587666 | Er dao zao | VI | *E1* | ***e2*** | *E3-Mi* |
| PI 407801 |  | VI | *E1* | *E2* | ***e3-tr*** |
| PI 424391 | KAS 521-15 | VI | *E1* | *E2* | ***e3-tr*** |
| PI 59845 | Sohgetsu | VI | *E1* | *E2* | *E3-Ha* |
| PI 408342 | KAERI 590-6 | VI | *E1* | *E2* | *E3-Mi* |
| PI 594629 | Xiao hua lian | VI | *E1* | *E2* | *E3-Ha* |
| PI 594770 A | Fu sui chang ping hei dou | VI | *E1* | *E2* | *E3-Ha* |
| PI 603516 | Xiao ma yi dan | VI | *E1* | *E2* | *E3-Mi* |
| PI 97094 | 1243 | VII | *E1* | *E2* | *E3-Ha* |
| PI 587552 | Nan jing da ping ding huang yi No.1 | VII | *E1* | *E2* | *E3-Mi* |
| PI 578457 A^*^ | May den | VIII | *E1* | *E2* | *E3* |
| PI 587799 | Wu chang zao huang dou | VIII | *E1* | *E2* | *E3-Ha* |
| PI 423967 | Nabeshima | IX | *E1* | *E2* | *E3-Mi* |
| PI 587906 | Huang dou | IX | *E1* | *E2* | *E3-Mi* |
| PI 588047 | Huang ke wu dou | IX | *E1* | *E2* | *E3-Ha* |
| PI 594554 | Huang pi tian dou | IX | *E1* | *E2* | *E3-Mi* |
| PI 594597 | Ning yuan ba yue huang | IX | *E1* | *E2* | *E3-Mi* |
| PI 594773 | Fu sui qu li dou | IX | *E1* | *E2* | *E3-Ha* |
| PI 594788 | Da zao dou | IX | *E1* | *E2* | *E3-Mi* |
| PI 587946 | Ping nan qiu da dou | X | *E1* | *E2* | *E3-Mi* |
| PI 588000 | Shi yue huang | X | *E1* | *E2* | *E3-Ha* |
| IGDB-081^*^ | Qing Dou |  | ***e1-as*** | ***e2*** | ***e3-tr*** |
| IGDB-083^*^ | PI Xian Da Zi Huo Cao |  | ***e1-as*** | *E2* | *E3* |
| IGDB-190^*^ | Hei He Xiao Huang Dou |  | *E1* | ***e2*** | ***e3-tr*** |
| IGDB-063^*^ | Yu Shi Dou |  | *E1* | ***e2*** | *E3* |
| IGDB-067^*^ | Xin Xian Xiao Huang Dou |  | *E1* | ***e2*** | *E3* |
| IGDB-068^*^ | Xiao Mi Dou |  | *E1* | ***e2*** | *E3* |
| IGDB-069^*^ | Xiao Huang Dou |  | *E1* | ***e2*** | *E3* |
| IGDB-071^*^ | Xiao Bai Dou |  | *E1* | ***e2*** | *E3* |
| IGDB-073^*^ | Xia Men Teng Zai Dou |  | *E1* | ***e2*** | *E3* |
| IGDB-076^*^ | Tian E Dan |  | *E1* | ***e2*** | *E3* |
| IGDB-080^*^ | Sha Xian Qin Dou |  | *E1* | ***e2*** | *E3* |
| IGDB-082^*^ | PI Xian Nian Zhuang Liu Yue Xian |  | *E1* | ***e2*** | *E3* |
| IGDB-177^*^ | Pei Xian Xiao You Dou |  | *E1* | ***e2*** | *E3* |
| IGDB-179^*^ | Ni Dou |  | *E1* | ***e2*** | *E3* |
| IGDB-181^*^ | Nan Guan Xiao PI Qing |  | *E1* | ***e2*** | *E3* |
| IGDB-182^*^ | Long quan Da dou |  | *E1* | ***e2*** | *E3* |
| IGDB-184^*^ | Jin Huang No. 35 |  | *E1* | ***e2*** | *E3* |
| IGDB-188^*^ | Hong Hu Liu Yue Bao |  | *E1* | ***e2*** | *E3* |
| IGDB-189^*^ | Hei Wa Shi Dou |  | *E1* | ***e2*** | *E3* |
| IGDB-192^*^ | Guang Rao Da Qing Dou |  | *E1* | ***e2*** | *E3* |
| IGDB-195^*^ | Dai Mi Dou |  | *E1* | ***e2*** | *E3* |
| IGDB-199^*^ | Cu Dou |  | *E1* | ***e2*** | *E3* |
| IGDB-200^*^ | Bin Hai Da huang Ke Zi Jia |  | *E1* | ***e2*** | *E3* |
| IGDB-202^*^ | Bai Mao Dou |  | *E1* | ***e2*** | *E3* |
| IGDB-064^*^ | Yu Jiang Wu Yue Niu Mao Huang |  | *E1* | *E2* | *E3* |
| IGDB-065^*^ | You Pi Zhi Hei Dou |  | *E1* | *E2* | *E3* |
| IGDB-066^*^ | Yi Zheng Da Li Huang Dou |  | *E1* | *E2* | *E3* |
| IGDB-070^*^ | Xiao Bai Qi |  | *E1* | *E2* | *E3* |
| IGDB-074^*^ | Xia Hei Dou |  | *E1* | *E2* | *E3* |
| IGDB-077^*^ | Tai Xin Niu Mao Huang Yi |  | *E1* | *E2* | *E3* |
| IGDB-078^*^ | Sha Xin Dou |  | *E1* | *E2* | *E3* |
| IGDB-079^*^ | Sha Xian Wu Dou |  | *E1* | *E2* | *E3* |
| IGDB-180^*^ | Ni Ding Hua Mei Dou |  | *E1* | *E2* | *E3* |
| IGDB-183^*^ | Jin Shan Cha Zhu Shi Dou |  | *E1* | *E2* | *E3* |
| IGDB-185^*^ | Ji Shan De Da Li Hei Dou |  | *E1* | *E2* | *E3* |
| IGDB-187^*^ | Hong Zhu Dou |  | *E1* | *E2* | *E3* |
| IGDB-191^*^ | He Dou |  | *E1* | *E2* | *E3* |
| IGDB-194^*^ | Dong Shan Bai Ma Dou |  | *E1* | *E2* | *E3* |
| IGDB-196^*^ | Da Tun Xiao Hei Dou |  | *E1* | *E2* | *E3* |
| IGDB-198^*^ | Da Li Huang |  | *E1* | *E2* | *E3* |

^*^*E* genotypes were determined from the Zhou et al. 2015 resequencing dataset (22). *E1* and *E2* were identified by the causative SNP, and functional *E3* and nonfunctional *e3-tr* were identified by haplotype analysis of the *E3* region using SNPViz (23). Functional *E3* was not classified as *E3-Ha* or *E3-Mi*.

**Table S2.** *E* genotypes of major maturity genes of Chinese cultivars

| Code ID^*^ | Name | *E1/e1-as* | *E2/e2* | *E3/e3-tr* |
| --- | --- | --- | --- | --- |
| IGDB-215 | Su Nong No.33 | ***e1-as*** | ***e2*** | ***e3-tr*** |
| IGDB-216 | Su Nong No.25 | ***e1-as*** | ***e2*** | ***e3-tr*** |
| IGDB-222 | Harbin 91-6065 | ***e1-as*** | ***e2*** | *E3* |
| IGDB-281 | Ji Yu No.90 | ***e1-as*** | ***e2*** | *E3* |
| IGDB-283 | Hei He No.1 | ***e1-as*** | ***e2*** | *E3* |
| IGDB-291 | Fen Dou No.85 | ***e1-as*** | ***e2*** | *E3* |
| IGDB-298 | Dong Nong No.26 | ***e1-as*** | ***e2*** | *E3* |
| IGDB-280 | Jin Da No.26 | ***e1-as*** | *E2* | *E3* |
| IGDB-289 | Fen Dou No.88 | ***e1-as*** | *E2* | *E3* |
| IGDB-290 | Fen Dou No.86 | ***e1-as*** | *E2* | *E3* |
| IGDB-292 | Fen Dou No.79 | ***e1-as*** | *E2* | *E3* |
| IGDB-293 | Fen Dou No.78 | ***e1-as*** | *E2* | *E3* |
| IGDB-295 | Fen Dou No.63 | ***e1-as*** | *E2* | *E3* |
| IGDB-302 | Cang Dou-11 | ***e1-as*** | *E2* | *E3* |
| IGDB-213 | Tie Feng No.22 | *E1* | ***e2*** | ***e3-tr*** |
| IGDB-217 | Su Nong No.14 | *E1* | ***e2*** | ***e3-tr*** |
| IGDB-218 | Su Nong No.10 | *E1* | ***e2*** | ***e3-tr*** |
| IGDB-271 | Liao Dou No.17 | *E1* | ***e2*** | ***e3-tr*** |
| IGDB-284 | He Feng No.25 | *E1* | ***e2*** | ***e3-tr*** |
| IGDB-285 | He Feng No.23 | *E1* | ***e2*** | ***e3-tr*** |
| IGDB-201 | Beijing-IGDB-1 | *E1* | ***e2*** | *E3* |
| IGDB-204 | Zhong Huang No.50 | *E1* | ***e2*** | *E3* |
| IGDB-205 | Zhong Huang No.40 | *E1* | ***e2*** | *E3* |
| IGDB-207 | Zhong Huang No.35 | *E1* | ***e2*** | *E3* |
| IGDB-209 | Zhong Huang No.14 | *E1* | ***e2*** | *E3* |
| IGDB-211 | Xi Zhang Da Dou No.20 | *E1* | ***e2*** | *E3* |
| IGDB-212 | Wei No.6823 | *E1* | ***e2*** | *E3* |
| IGDB-214 | Tai Wan No.1 | *E1* | ***e2*** | *E3* |
| IGDB-219 | Shu Xian No.205 | *E1* | ***e2*** | *E3* |
| IGDB-220 | Sheng Dou No.9 | *E1* | ***e2*** | *E3* |
| IGDB-221 | Shen Li No.3 | *E1* | ***e2*** | *E3* |
| IGDB-261 | Jilin 21 | *E1* | ***e2*** | *E3* |
| IGDB-268 | Lu Dou No. 11 | *E1* | ***e2*** | *E3* |
| IGDB-269 | Liao Dou No.3 | *E1* | ***e2*** | *E3* |
| IGDB-270 | Liao Dou No. 21 | *E1* | ***e2*** | *E3* |
| IGDB-272 | Liao Dou No.15 | *E1* | ***e2*** | *E3* |
| IGDB-274 | Jiu Nong No.30 | *E1* | ***e2*** | *E3* |
| IGDB-275 | Jin Da No.75 | *E1* | ***e2*** | *E3* |
| IGDB-278 | Jin Da No.62 | *E1* | ***e2*** | *E3* |
| IGDB-286 | He Dou No.13 | *E1* | ***e2*** | *E3* |
| IGDB-294 | Fen Dou No.65 | *E1* | ***e2*** | *E3* |
| IGDB-299 | Chang Nong No.16 | *E1* | ***e2*** | *E3* |
| IGDB-300 | Chang Nong No. 15 | *E1* | ***e2*** | *E3* |
| IGDB-301 | Chang Nong No. 13 | *E1* | ***e2*** | *E3* |
| IGDB-206 | Zhong Huang No.38 | *E1* | *E2* | *E3* |
| IGDB-276 | Jin Da No.73 | *E1* | *E2* | *E3* |
| IGDB-279 | Jin Da No.52 | *E1* | *E2* | *E3* |
| IGDB-288 | Fen Dou No.89 | *E1* | *E2* | *E3* |

*Chinese cultivars are identified by a code from Zhou et al. 2015 (22). *E* genotypes were determined from the Zhou et al. 2015 resequencing dataset. *E1* and *E2* were identified by the causative SNP, and functional *E3* and nonfunctional *e3-tr* were identified by haplotype analysis of the *E3* region using SNPViz (23). Functional *E3* was not classified as *E3-Ha* or *E3-Mi*.

**Table S3.** *E* genotypes of major maturity genes in US cultivars

| **PI Number** | **Name** | **MG** | ***E1/e1-as*** | ***E2/e2*** | ***E3/e3-tr*** |
| --- | --- | --- | --- | --- | --- |
| PI 508083 | Dassel | 0 | ***e1-as*** | ***e2*** | ***e3-tr*** |
| PI 513382 | Glenwood | 0 | ***e1-as*** | ***e2*** | ***e3-tr*** |
| PI 591433^*^ | OT94-37 | 0 | ***e1-as*** | ***e2*** | ***e3-tr*** |
| PI 542403 | Dawson | 0 | ***e1-as*** | ***e2*** | *E3-Ha* |
| PI 548638 | OAC Libra | 0 | ***e1-as*** | ***e2*** | *E3-Ha* |
| PI 548643 | Maple Glen | 0 | ***e1-nl*** | ***e2*** | *E3-Ha* |
| PI 548644 | OAC Musca | 0 | ***e1-as*** | ***e2*** | *E3-Ha* |
| PI 591431^*^ | OT94-49 | 0 | *E1* | ***e2*** | ***e3-tr*** |
| PI 591435^*, †^ | OT95-41 | I | ***e1-as*** | ***e2*** | *E3-Ha* |
| PI 548524 | Weber | I | ***e1-as*** | *E2* | ***e3-tr*** |
| PI 547716^*^ | L62-667 | II | ***e1-as*** | ***e2*** | ***e3-tr*** |
| PI 533655 | Burlison | II | ***e1-as*** | ***e2*** | *E3-Mi* |
| PI 547686^*^ | L62-956 | II | ***e1-as*** | ***e2*** | *E3* |
| PI 547690^*^ | L63-1212 | II | ***e1-as*** | ***e2*** | *E3* |
| PI 547779^*^ | L72D-4110 | II | ***e1-as*** | ***e2*** | *E3* |
| PI 548512 | Century | II | ***e1-as*** | ***e2*** | *E3-Ha* |
| PI 548573^†^ | Harosoy | II | ***e1-as*** | ***e2*** | *E3-Ha* |
| PI 525453 | Conrad | II | ***e1-as*** | *E2* | ***e3-tr*** |
| PI 540552 | Hoyt | II | ***e1-as*** | *E2* | ***e3-tr*** |
| PI 548520 | Preston | II | ***e1-as*** | *E2* | ***e3-tr*** |
| PI 548540^*^ | Corsoy | II | ***e1-as*** | *E2* | ***e3-tr*** |
| PI 548565^*^ | Gnome | II | ***e1-as*** | *E2* | *E3* |
| PI 591541^*^ | L74-102 | II | *E1* | ***e2*** | ***e3-tr*** |
| PI 548190^*^ | T176 | II | *E1* | ***e2*** | *E3* |
| PI 591511^*^ | L89-1581 | III | ***e1-as*** | *E2* | *E3* |
| PI 548182^*^ | T157 | III | ***e1-as*** | ***e2*** | ***e3-tr*** |
| PI 548631^*^ | Williams | III | ***e1-as*** | *E2* | *E3* |
| PI 536635 | Sprite | III | ***e1-as*** | *E2* | *E3-Ha* |
| PI 548634 | Zane | III | ***e1-as*** | *E2* | *E3-Ha* |
| PI 556511 | A3127 | III | ***e1-as*** | *E2* | *E3-Ha* |
| PI 515961 | Pennyrile | IV | ***e1-as*** | *E2* | *E3-Ha* |
| PI 547409^*^ | L62-1251 | IV | ***e1-as*** | *E2* | *E3* |
| PI 547460^*^ | L64-1083 | IV | ***e1-as*** | *E2* | *E3* |
| PI 547488^*^ | L67-3207 | IV | ***e1-as*** | *E2* | *E3* |
| PI 547562^*^ | L72-2157 | IV | ***e1-as*** | *E2* | *E3* |
| PI 591495^*^ | L93-2740 | IV | ***e1-as*** | *E2* | *E3* |
| PI 536636 | Ripley | IV | *E1* | ***e2*** | *E3-Ha* |
| PI 548604 | Pershing | IV | *E1* | *E2* | *E3-Mi* |
| PI 518664 | Hutcheson | V | *E1* | *E2* | *E3-Ha* |
| PI 508266 | Young | VI | *E1* | *E2* | *E3-Mi* |
| PI 533602 | Lloyd | VI | *E1* | *E2* | *E3-Ha* |
| PI 548985 | Kershaw | VI | *E1* | *E2* | *E3-Ha* |
| PI 522236 | Thomas | VII | *E1* | *E2* | *E3-Ha* |
| PI 553047 | Gordon | VII | *E1* | *E2* | *E3-Ha* |
| PI 536637 | Perrin | VIII | *E1* | *E2* | *E3-Ha* |
| PI 634883^*^ | Elf |  | ***e1-as*** | *E2* | *E3* |

^*^*E* genotypes were determined from the Zhou et al. 2015 resequencing dataset (22). *E1* and *E2* were identified by the causative SNP, and functional *E3* and nonfunctional *e3-tr* were identified by haplotype analysis of the *E3* region using SNPViz (23). Functional *E3* was not classified as *E3-Ha* or *E3-Mi*.

^†^The *E3* genotype was taken from Tardivel et al. 2014.

**Table S4.** E genotypes of major maturity genes of the PVP collection

| **PI number** | **Name** | **PVP issued** | **MG** | ***E1/e1*** | ***E2/e2*** | ***E3/e3-tr*** |
| --- | --- | --- | --- | --- | --- | --- |
| PI 556751 | KG20 | 1986 | 00 | ***e1-as*** | ***e2*** | ***e3-tr*** |
| PI 562372 | Agassiz | 1994 | 00 | ***e1-as*** | ***e2*** | ***e3-tr*** |
| PI 556749 | KG30 | 1986 | 00 | ***e1-nl*** | ***e2*** | *E3-Ha* |
| PI 556812 | A0949 | 1986 | 00 | ***e1-as*** | ***e2*** | *E3-Ha* |
| PI 556517 | BEECHWOOD | 1977 | 0 | ***e1-fs*** | ***e2*** | ***e3-tr*** |
| PI 508083 | Dassel | 1987 | 0 | ***e1-as*** | ***e2*** | ***e3-tr*** |
| PI 513382 | Glenwood | 1988 | 0 | ***e1-as*** | ***e2*** | ***e3-tr*** |
| PI 548560 | Evans | 1975 | 0 | ***e1-as*** | ***e2*** | ***e3-tr*** |
| PI 548615 | Simpson | 1983 | 0 | ***e1-as*** | ***e2*** | ***e3-tr*** |
| PI 555463 | BT1422 | 1992 | 0 | ***e1-as*** | ***e2*** | ***e3-tr*** |
| PI 556643 | S09-90 | 1982 | 0 | ***e1-as*** | ***e2*** | ***e3-tr*** |
| PI 556756 | J-72 | 1985 | 0 | ***e1-as*** | ***e2*** | ***e3-tr*** |
| PI 556771 | 9061 | 1986 | 0 | ***e1-as*** | ***e2*** | ***e3-tr*** |
| PI 556836 | 9091 | 1987 | 0 | ***e1-as*** | ***e2*** | ***e3-tr*** |
| PI 556844 | S06-57 | 1987 | 0 | ***e1-as*** | ***e2*** | ***e3-tr*** |
| PI 556849 | A0358 | 1987 | 0 | ***e1-as*** | ***e2*** | ***e3-tr*** |
| PI 556895 | S07-80 | 1991 | 0 | ***e1-as*** | ***e2*** | ***e3-tr*** |
| PI 561584 | 9062 | 1994 | 0 | ***e1-as*** | ***e2*** | ***e3-tr*** |
| PI 562373 | Lambert | 1994 | 0 | ***e1-as*** | ***e2*** | ***e3-tr*** |
| PI 542403 | Dawson | 1985 | 0 | ***e1-as*** | ***e2*** | *E3-Ha* |
| PI 542404 | Ozzie | 1985 | 0 | ***e1-as*** | ***e2*** | *E3-Ha* |
| PI 556767 | B095 | 1985 | 0 | ***e1-as*** | ***e2*** | *E3-Ha* |
| PI 556737 | J82 | 1985 | 0 | ***e1-as*** | *E2* | ***e3-tr*** |
| PI 556856 | NATTOKING K86 | 1988 | 0 | ***e1-as*** | *E2* | ***e3-tr*** |
| PI 537096 | Minnatto | 1991 | 0 | *E1* | ***e2*** | ***e3-tr*** |
| PI 542065 | 9162 | 1993 | I | ***e1-as*** | ***e2*** | ***e3-tr*** |
| PI 542393 | BT 1790 | 1994 | I | ***e1-as*** | ***e2*** | ***e3-tr*** |
| PI 548681 | SRF 100 | 1973 | I | ***e1-as*** | ***e2*** | ***e3-tr*** |
| PI 556490 | PETERSON 85 | 1975 | I | ***e1-as*** | ***e2*** | ***e3-tr*** |
| PI 556512 | 118-11 | 1977 | I | ***e1-as*** | ***e2*** | ***e3-tr*** |
| PI 556543 | DOTSON | 1978 | I | ***e1-as*** | ***e2*** | ***e3-tr*** |
| PI 556554 | AP 10 | 1979 | I | ***e1-as*** | ***e2*** | ***e3-tr*** |
| PI 556574 | TC 137 | 1980 | I | ***e1-as*** | ***e2*** | ***e3-tr*** |
| PI 556598 | DSR-141 | 1981 | I | ***e1-as*** | ***e2*** | ***e3-tr*** |
| PI 556613 | L4404 | 1981 | I | ***e1-as*** | ***e2*** | ***e3-tr*** |
| PI 556638 | A1179 | 1982 | I | ***e1-as*** | ***e2*** | ***e3-tr*** |
| PI 556665 | 1282 | 1983 | I | ***e1-as*** | ***e2*** | ***e3-tr*** |
| PI 556682 | AP 120 | 1984 | I | ***e1-as*** | ***e2*** | ***e3-tr*** |
| PI 556694 | L4504 | 1983 | I | ***e1-as*** | ***e2*** | ***e3-tr*** |
| PI 556695 | L4503 | 1983 | I | ***e1-as*** | ***e2*** | ***e3-tr*** |
| PI 556776 | A1214 | 1986 | I | ***e1-as*** | ***e2*** | ***e3-tr*** |
| PI 556785 | KG60 | 1986 | I | ***e1-as*** | ***e2*** | ***e3-tr*** |
| PI 556837 | 9181 | 1987 | I | ***e1-as*** | ***e2*** | ***e3-tr*** |
| PI 556853 | AP 1776 | 1987 | I | ***e1-as*** | ***e2*** | ***e3-tr*** |
| PI 556872 | 9111 | 1988 | I | ***e1-as*** | ***e2*** | ***e3-tr*** |
| PI 556883 | AP 1650 | 1989 | I | ***e1-as*** | ***e2*** | ***e3-tr*** |
| PI 556896 | S17-18 | 1991 | I | ***e1-as*** | ***e2*** | ***e3-tr*** |
| PI 508084 | Sibley | 1987 | I | ***e1-as*** | ***e2*** | *E3-Ha* |
| PI 540554 | Bell | 1992 | I | ***e1-as*** | ***e2*** | *E3-Ha* |
| PI 542042 | Kato | 1991 | I | ***e1-as*** | ***e2*** | *E3-Ha* |
| PI 542057 | 9191 | 1992 | I | ***e1-as*** | ***e2*** | *E3-Ha* |
| PI 542399 | BT 2585 | 1992 | I | ***e1-as*** | ***e2*** | *E3-Ha* |
| PI 546038 | Kasota | 1992 | I | ***e1-as*** | ***e2*** | *E3-Ha* |
| PI 546487 | Archer | 1992 | I | ***e1-as*** | ***e2*** | *E3-Ha* |
| PI 548536 | Coles | 1978 | I | ***e1-as*** | ***e2*** | *E3-Ha* |
| PI 548581 | Hodgson 78 | 1978 | I | ***e1-as*** | ***e2*** | *E3-Ha* |
| PI 548618 | Vinton | 1980 | I | ***e1-as*** | ***e2*** | *E3-Ha* |
| PI 548625 | Vinton 81 | 1982 | I | ***e1-as*** | ***e2*** | *E3-Ha* |
| PI 548683 | SRF 150 | 1973 | I | ***e1-as*** | ***e2*** | *E3-Ha* |
| PI 550728 | 9131 | 1992 | I | ***e1-as*** | ***e2*** | *E3-Ha* |
| PI 550740 | A1662 | 1992 | I | ***e1-as*** | ***e2*** | *E3-Ha* |
| PI 555464 | BT 2877 | 1993 | I | ***e1-as*** | ***e2*** | *E3-Ha* |
| PI 556501 | S 1346 | 1976 | I | ***e1-as*** | ***e2*** | *E3-Ha* |
| PI 556502 | S 1244 | 1977 | I | ***e1-as*** | ***e2*** | *E3-Ha* |
| PI 556503 | B186 | 1975 | I | ***e1-as*** | ***e2*** | *E3-Ha* |
| PI 556507 | A1564 | 1977 | I | ***e1-as*** | ***e2*** | *E3-Ha* |
| PI 556519 | FFR 111 | 1977 | I | ***e1-as*** | ***e2*** | *E3-Ha* |
| PI 556520 | GUTWEIN 180 | 1977 | I | ***e1-as*** | ***e2*** | *E3-Ha* |
| PI 556522 | DS 160 | 1977 | I | ***e1-as*** | ***e2*** | *E3-Ha* |
| PI 556557 | SRF 150P | 1979 | I | ***e1-as*** | ***e2*** | *E3-Ha* |
| PI 556591 | 1981 | 1980 | I | ***e1-as*** | ***e2*** | *E3-Ha* |
| PI 556599 | DSR-171 | 1981 | I | ***e1-as*** | ***e2*** | *E3-Ha* |
| PI 556602 | DSR-120 | 1981 | I | ***e1-as*** | ***e2*** | *E3-Ha* |
| PI 556634 | SRF 101 | 1982 | I | ***e1-as*** | ***e2*** | *E3-Ha* |
| PI 556637 | A1937 | 1981 | I | ***e1-as*** | ***e2*** | *E3-Ha* |
| PI 556644 | S18-84 | 1982 | I | ***e1-as*** | ***e2*** | *E3-Ha* |
| PI 556657 | B152 | 1982 | I | ***e1-as*** | ***e2*** | *E3-Ha* |
| PI 556658 | S14-60 | 1984 | I | ***e1-as*** | ***e2*** | *E3-Ha* |
| PI 556667 | 1082 | 1983 | I | ***e1-as*** | ***e2*** | *E3-Ha* |
| PI 556750 | KG70 | 1986 | I | ***e1-as*** | ***e2*** | *E3-Ha* |
| PI 556765 | S15-50 | 1985 | I | ***e1-as*** | ***e2*** | *E3-Ha* |
| PI 556768 | L1771 | 1986 | I | ***e1-as*** | ***e2*** | *E3-Ha* |
| PI 556777 | A1895 | 1986 | I | ***e1-as*** | ***e2*** | *E3-Ha* |
| PI 556779 | A1525 | 1986 | I | ***e1-as*** | ***e2*** | *E3-Ha* |
| PI 556843 | B117 | 1988 | I | ***e1-as*** | ***e2*** | *E3-Ha* |
| PI 556873 | 9161 | 1988 | I | ***e1-as*** | ***e2*** | *E3-Ha* |
| PI 556877 | DSR-128 | 1988 | I | ***e1-as*** | ***e2*** | *E3-Ha* |
| PI 556879 | DSR-155 | 1988 | I | ***e1-as*** | ***e2*** | *E3-Ha* |
| PI 556897 | S19-90 | 1991 | I | ***e1-as*** | ***e2*** | *E3-Ha* |
| PI 556908 | 9121 | 1991 | I | ***e1-as*** | ***e2*** | *E3-Ha* |
| PI 556926 | TK 89 | 1991 | I | ***e1-as*** | ***e2*** | *E3-Ha* |
| PI 556927 | A1929 | 1991 | I | ***e1-as*** | ***e2*** | *E3-Ha* |
| PI 557010 | Bert | 1992 | I | ***e1-as*** | ***e2*** | *E3-Ha* |
| PI 557011 | Leslie | 1992 | I | ***e1-as*** | ***e2*** | *E3-Ha* |
| PI 561211 | 3172 | 1994 | I | ***e1-as*** | ***e2*** | *E3-Ha* |
| PI 562374 | Parker | 1994 | I | ***e1-as*** | ***e2*** | *E3-Ha* |
| PI 562628 | CX121 | 1994 | I | ***e1-as*** | ***e2*** | *E3-Ha* |
| PI 564524 | Alpha | 1994 | I | ***e1-as*** | ***e2*** | *E3-Ha* |
| PI 568240 | A1395 | 1995 | I | ***e1-as*** | ***e2*** | *E3-Mi* |
| PI 542056 | 9171 | 1992 | I | ***e1-as*** | *E2* | ***e3-tr*** |
| PI 548524 | Weber | 1980 | I | ***e1-as*** | *E2* | ***e3-tr*** |
| PI 548526 | Hardin | 1982 | I | ***e1-as*** | *E2* | ***e3-tr*** |
| PI 548588 | Lakota | 1982 | I | ***e1-as*** | *E2* | ***e3-tr*** |
| PI 556593 | 1677 | 1980 | I | ***e1-as*** | *E2* | ***e3-tr*** |
| PI 556755 | J-8287 | 1986 | I | ***e1-as*** | *E2* | ***e3-tr*** |
| PI 556757 | J-8389 | 1985 | I | ***e1-as*** | *E2* | ***e3-tr*** |
| PI 561205 | S12-22 | 1994 | I | ***e1-as*** | *E2* | ***e3-tr*** |
| PI 548561 | Hodgson | 1975 | I | *E1* | ***e2*** | *E3-Ha* |
| PI 556604 | HP 20-20 | 1981 | II | ***e1-as*** | ***e2*** | ***e3-tr*** |
| PI 556605 | HS 220 | 1981 | II | ***e1-as*** | ***e2*** | ***e3-tr*** |
| PI 556838 | 9202 | 1988 | II | ***e1-as*** | ***e2*** | ***e3-tr*** |
| PI 556898 | S20-26 | 1991 | II | ***e1-as*** | ***e2*** | ***e3-tr*** |
| PI 561582 | 9232 | 1994 | II | ***e1-as*** | ***e2*** | ***e3-tr*** |
| PI 518666 | Elgin 87 | 1988 | II | ***e1-as*** | ***e2*** | *E3-Ha* |
| PI 537094 | Kenwood | 1992 | II | ***e1-as*** | ***e2*** | *E3-Ha* |
| PI 537095 | Marcus | 1992 | II | ***e1-as*** | ***e2*** | *E3-Ha* |
| PI 540880 | S20-20 | 1992 | II | ***e1-as*** | ***e2*** | *E3-Ha* |
| PI 540882 | S28-18 | 1991 | II | ***e1-as*** | ***e2*** | *E3-Ha* |
| PI 542768 | Sturdy | 1991 | II | ***e1-as*** | ***e2*** | *E3-Ha* |
| PI 548511 | Beeson 80 | 1980 | II | ***e1-as*** | ***e2*** | *E3-Ha* |
| PI 548512 | Century | 1980 | II | ***e1-as*** | ***e2*** | *E3-Ha* |
| PI 548513 | Wells II | 1979 | II | ***e1-as*** | ***e2*** | *E3-Ha* |
| PI 548521 | BSR 201 | 1984 | II | ***e1-as*** | ***e2*** | *E3-Ha* |
| PI 548529 | Century 84 | 1985 | II | ***e1-as*** | ***e2*** | *E3-Ha* |
| PI 548569 | Hack | 1986 | II | ***e1-as*** | ***e2*** | *E3-Ha* |
| PI 548583 | Keller | 1985 | II | ***e1-as*** | ***e2*** | *E3-Ha* |
| PI 548584 | Miami | 1985 | II | ***e1-as*** | ***e2*** | *E3-Ha* |
| PI 548605 | Platte | 1984 | II | ***e1-as*** | ***e2*** | *E3-Ha* |
| PI 548630 | Wells | 1974 | II | ***e1-as*** | ***e2*** | *E3-Ha* |
| PI 548688 | XK505 | 1974 | II | ***e1-as*** | ***e2*** | *E3-Ha* |
| PI 548689 | B 216 | 1976 | II | ***e1-as*** | ***e2*** | *E3-Ha* |
| PI 548690 | S 1492 | 1976 | II | ***e1-as*** | ***e2*** | *E3-Ha* |
| PI 550729 | 9231 | 1993 | II | ***e1-as*** | ***e2*** | *E3-Ha* |
| PI 556474 | SRF 200 | 1974 | II | ***e1-as*** | ***e2*** | *E3-Ha* |
| PI 556483 | 3034 | 1974 | II | ***e1-as*** | ***e2*** | *E3-Ha* |
| PI 556492 | AGRIPRO 20 | 1975 | II | ***e1-as*** | ***e2*** | *E3-Ha* |
| PI 556495 | A2656 | 1976 | II | ***e1-as*** | ***e2*** | *E3-Ha* |
| PI 556497 | VORIS 295 | 1976 | II | ***e1-as*** | ***e2*** | *E3-Ha* |
| PI 556498 | P83-14 | 1976 | II | ***e1-as*** | ***e2*** | *E3-Ha* |
| PI 556509 | A2444 | 1977 | II | ***e1-as*** | ***e2*** | *E3-Ha* |
| PI 556513 | McKoy 1100 | 1976 | II | ***e1-as*** | ***e2*** | *E3-Ha* |
| PI 556518 | S1578 | 1977 | II | ***e1-as*** | ***e2*** | *E3-Ha* |
| PI 556525 | CX290 | 1977 | II | ***e1-as*** | ***e2*** | *E3-Ha* |
| PI 556547 | ROCKFORD | 1978 | II | ***e1-as*** | ***e2*** | *E3-Ha* |
| PI 556548 | A2858 | 1979 | II | ***e1-as*** | ***e2*** | *E3-Ha* |
| PI 556552 | RS 2300 | 1979 | II | ***e1-as*** | ***e2*** | *E3-Ha* |
| PI 556555 | GT 1200 | 1979 | II | ***e1-as*** | ***e2*** | *E3-Ha* |
| PI 556575 | AP 225C | 1980 | II | ***e1-as*** | ***e2*** | *E3-Ha* |
| PI 556580 | DUKE | 1980 | II | ***e1-as*** | ***e2*** | *E3-Ha* |
| PI 556585 | S2596 | 1980 | II | ***e1-as*** | ***e2*** | *E3-Ha* |
| PI 556622 | L4303 | 1982 | II | ***e1-as*** | ***e2*** | *E3-Ha* |
| PI 556624 | HS 235 | 1982 | II | ***e1-as*** | ***e2*** | *E3-Ha* |
| PI 556627 | AP 230 | 1982 | II | ***e1-as*** | ***e2*** | *E3-Ha* |
| PI 556628 | CS20 | 1981 | II | ***e1-as*** | ***e2*** | *E3-Ha* |
| PI 556640 | 2180 | 1982 | II | ***e1-as*** | ***e2*** | *E3-Ha* |
| PI 556653 | B203 | 1983 | II | ***e1-as*** | ***e2*** | *E3-Ha* |
| PI 556655 | 2400 | 1983 | II | ***e1-as*** | ***e2*** | *E3-Ha* |
| PI 556672 | GL 2250 | 1983 | II | ***e1-as*** | ***e2*** | *E3-Ha* |
| PI 556674 | GUTWEIN 225 | 1984 | II | ***e1-as*** | ***e2*** | *E3-Ha* |
| PI 556675 | SRF 205 | 1984 | II | ***e1-as*** | ***e2*** | *E3-Ha* |
| PI 556677 | HP 2530 | 1982 | II | ***e1-as*** | ***e2*** | *E3-Ha* |
| PI 556698 | BELLATTI-R-77-84 | 1982 | II | ***e1-as*** | ***e2*** | *E3-Ha* |
| PI 556712 | 90210-30266 | 1983 | II | ***e1-as*** | ***e2*** | *E3-Ha* |
| PI 556713 | 60524-30575 | 1983 | II | ***e1-as*** | ***e2*** | *E3-Ha* |
| PI 556715 | 90339-30435 | 1983 | II | ***e1-as*** | ***e2*** | *E3-Ha* |
| PI 556724 | S23-03 | 1986 | II | ***e1-as*** | ***e2*** | *E3-Ha* |
| PI 556729 | A2522 | 1985 | II | ***e1-as*** | ***e2*** | *E3-Ha* |
| PI 556746 | RIVERSIDE 303C | 1986 | II | ***e1-as*** | ***e2*** | *E3-Ha* |
| PI 556763 | S24-24 | 1985 | II | ***e1-as*** | ***e2*** | *E3-Ha* |
| PI 556783 | A2187 | 1987 | II | ***e1-as*** | ***e2*** | *E3-Ha* |
| PI 556786 | J-231 | 1987 | II | ***e1-as*** | ***e2*** | *E3-Ha* |
| PI 556797 | 9201 | 1987 | II | ***e1-as*** | ***e2*** | *E3-Ha* |
| PI 556798 | 9251 | 1987 | II | ***e1-as*** | ***e2*** | *E3-Ha* |
| PI 556810 | AP 2190 | 1986 | II | ***e1-as*** | ***e2*** | *E3-Ha* |
| PI 556821 | B236 | 1988 | II | ***e1-as*** | ***e2*** | *E3-Ha* |
| PI 556822 | S23-12 | 1988 | II | ***e1-as*** | ***e2*** | *E3-Ha* |
| PI 556845 | S29-20 | 1989 | II | ***e1-as*** | ***e2*** | *E3-Ha* |
| PI 556850 | A2234 | 1987 | II | ***e1-as*** | ***e2*** | *E3-Ha* |
| PI 556863 | S21-98 | 1988 | II | ***e1-as*** | ***e2*** | *E3-Ha* |
| PI 556864 | S26-06 | 1988 | II | ***e1-as*** | ***e2*** | *E3-Ha* |
| PI 556881 | AP 2021 | 1989 | II | ***e1-as*** | ***e2*** | *E3-Ha* |
| PI 556885 | AP 2324 | 1991 | II | ***e1-as*** | ***e2*** | *E3-Ha* |
| PI 556920 | HS 2455 | 1991 | II | ***e1-as*** | ***e2*** | *E3-Ha* |
| PI 559397 | FFR 253 | 1992 | II | ***e1-as*** | ***e2*** | *E3-Ha* |
| PI 559398 | FFR 299 | 1994 | II | ***e1-as*** | ***e2*** | *E3-Ha* |
| PI 561212 | 3202 | 1994 | II | ***e1-as*** | ***e2*** | *E3-Ha* |
| PI 561589 | BT 2919 | 1994 | II | ***e1-as*** | ***e2*** | *E3-Ha* |
| PI 561717 | A2506 | 1995 | II | ***e1-as*** | ***e2*** | *E3-Ha* |
| PI 533655 | Burlison | 1991 | II | ***e1-as*** | ***e2*** | *E3-Mi* |
| PI 556646 | B242 | 1982 | II | ***e1-as*** | ***e2*** | *E3-Mi* |
| PI 525453 | Conrad | 1989 | II | ***e1-as*** | *E2* | ***e3-tr*** |
| PI 540451 | L2333 | 1991 | II | ***e1-as*** | *E2* | ***e3-tr*** |
| PI 540454 | A2396 | 1992 | II | ***e1-as*** | *E2* | ***e3-tr*** |
| PI 540881 | S25-15 | 1991 | II | ***e1-as*** | *E2* | ***e3-tr*** |
| PI 542058 | 9221 | 1993 | II | ***e1-as*** | *E2* | ***e3-tr*** |
| PI 542059 | 9241 | 1992 | II | ***e1-as*** | *E2* | ***e3-tr*** |
| PI 542060 | 9273 | 1992 | II | ***e1-as*** | *E2* | ***e3-tr*** |
| PI 542064 | Ardir | 1994 | II | ***e1-as*** | *E2* | ***e3-tr*** |
| PI 542392 | 38735L | 1994 | II | ***e1-as*** | *E2* | ***e3-tr*** |
| PI 546480 | CX210 | 1992 | II | ***e1-as*** | *E2* | ***e3-tr*** |
| PI 547094 | CX291 | 1992 | II | ***e1-as*** | *E2* | ***e3-tr*** |
| PI 548505 | Amcor | 1980 | II | ***e1-as*** | *E2* | ***e3-tr*** |
| PI 548617 | Vickery | 1981 | II | ***e1-as*** | *E2* | ***e3-tr*** |
| PI 554604 | CX267 | 1992 | II | ***e1-as*** | *E2* | ***e3-tr*** |
| PI 556482 | S1474 | 1974 | II | ***e1-as*** | *E2* | ***e3-tr*** |
| PI 556488 | PX 181-88 | 1975 | II | ***e1-as*** | *E2* | ***e3-tr*** |
| PI 556489 | P61-22 | 1975 | II | ***e1-as*** | *E2* | ***e3-tr*** |
| PI 556500 | SHAWNEE | 1976 | II | ***e1-as*** | *E2* | ***e3-tr*** |
| PI 556563 | S-48 | 1979 | II | ***e1-as*** | *E2* | ***e3-tr*** |
| PI 556588 | 2877 | 1980 | II | ***e1-as*** | *E2* | ***e3-tr*** |
| PI 556633 | J103 | 1982 | II | ***e1-as*** | *E2* | ***e3-tr*** |
| PI 556639 | A2680 | 1982 | II | ***e1-as*** | *E2* | ***e3-tr*** |
| PI 556641 | 2480 | 1982 | II | ***e1-as*** | *E2* | ***e3-tr*** |
| PI 556651 | CS24 | 1983 | II | ***e1-as*** | *E2* | ***e3-tr*** |
| PI 556681 | AP 240 | 1984 | II | ***e1-as*** | *E2* | ***e3-tr*** |
| PI 556735 | 9292 | 1985 | II | ***e1-as*** | *E2* | ***e3-tr*** |
| PI 556736 | 9271 | 1985 | II | ***e1-as*** | *E2* | ***e3-tr*** |
| PI 556761 | S27-10 | 1985 | II | ***e1-as*** | *E2* | ***e3-tr*** |
| PI 556769 | L2230 | 1986 | II | ***e1-as*** | *E2* | ***e3-tr*** |
| PI 556869 | 9272 | 1988 | II | ***e1-as*** | *E2* | ***e3-tr*** |
| PI 556890 | 29725K | 1991 | II | ***e1-as*** | *E2* | ***e3-tr*** |
| PI 561201 | A2242 | 1994 | II | ***e1-as*** | *E2* | ***e3-tr*** |
| PI 561206 | S24-92 | 1994 | II | ***e1-as*** | *E2* | ***e3-tr*** |
| PI 561208 | S28-01 | 1994 | II | ***e1-as*** | *E2* | ***e3-tr*** |
| PI 568243 | A2540 | 1995 | II | ***e1-as*** | *E2* | ***e3-tr*** |
| PI 576145 | Sandusky | 1995 | II | ***e1-as*** | *E2* | ***e3-tr*** |
| PI 576146 | Vertex | 1995 | II | ***e1-as*** | *E2* | ***e3-tr*** |
| PI 540452 | A2427 | 1992 | II | ***e1-as*** | *E2* | *E3-Ha* |
| PI 540453 | A2872 | 1992 | II | ***e1-as*** | *E2* | *E3-Ha* |
| PI 540556 | Jack | 1992 | II | ***e1-as*** | *E2* | *E3-Ha* |
| PI 540876 | HS 2533 | 1992 | II | ***e1-as*** | *E2* | *E3-Ha* |
| PI 540883 | S29-39 | 1992 | II | ***e1-as*** | *E2* | *E3-Ha* |
| PI 542055 | DSR-262 | 1994 | II | ***e1-as*** | *E2* | *E3-Ha* |
| PI 542710 | Chapman | 1994 | II | ***e1-as*** | *E2* | *E3-Ha* |
| PI 543857 | Gnome 85 | 1987 | II | ***e1-as*** | *E2* | *E3-Ha* |
| PI 548565 | Gnome | 1980 | II | ***e1-as*** | *E2* | *E3-Ha* |
| PI 548692 | AP 200 | 1980 | II | ***e1-as*** | *E2* | *E3-Ha* |
| PI 556470 | XK-515 | 1974 | II | ***e1-as*** | *E2* | *E3-Ha* |
| PI 556472 | XK-535 | 1974 | II | ***e1-as*** | *E2* | *E3-Ha* |
| PI 556484 | PX 185-44 | 1974 | II | ***e1-as*** | *E2* | *E3-Ha* |
| PI 556493 | CLASSIC I | 1975 | II | ***e1-as*** | *E2* | *E3-Ha* |
| PI 556523 | PL 6940104 | 1977 | II | ***e1-as*** | *E2* | *E3-Ha* |
| PI 556561 | K-77 | 1979 | II | ***e1-as*** | *E2* | *E3-Ha* |
| PI 556565 | SRF 250 | 1979 | II | ***e1-as*** | *E2* | *E3-Ha* |
| PI 556601 | DSR-232 | 1981 | II | ***e1-as*** | *E2* | *E3-Ha* |
| PI 556606 | HS 265 | 1981 | II | ***e1-as*** | *E2* | *E3-Ha* |
| PI 556679 | RS 2330 | 1984 | II | ***e1-as*** | *E2* | *E3-Ha* |
| PI 556689 | A2943 | 1984 | II | ***e1-as*** | *E2* | *E3-Ha* |
| PI 556692 | L4207 | 1983 | II | ***e1-as*** | *E2* | *E3-Ha* |
| PI 556693 | L4204 | 1983 | II | ***e1-as*** | *E2* | *E3-Ha* |
| PI 556705 | BIRCH | 1983 | II | ***e1-as*** | *E2* | *E3-Ha* |
| PI 556708 | DSR-227 | 1983 | II | ***e1-as*** | *E2* | *E3-Ha* |
| PI 556790 | DSR-287 | 1987 | II | ***e1-as*** | *E2* | *E3-Ha* |
| PI 556791 | DSR-297 | 1987 | II | ***e1-as*** | *E2* | *E3-Ha* |
| PI 556874 | 9293 | 1988 | II | ***e1-as*** | *E2* | *E3-Ha* |
| PI 556888 | CX298 | 1989 | II | ***e1-as*** | *E2* | *E3-Ha* |
| PI 556892 | 34870 | 1991 | II | ***e1-as*** | *E2* | *E3-Ha* |
| PI 556922 | HS 2821 | 1991 | II | ***e1-as*** | *E2* | *E3-Ha* |
| PI 556929 | A2543 | 1992 | II | ***e1-as*** | *E2* | *E3-Ha* |
| PI 561207 | S25-07 | 1994 | II | ***e1-as*** | *E2* | *E3-Ha* |
| PI 561718 | A2835 | 1994 | II | ***e1-as*** | *E2* | *E3-Ha* |
| PI 548616 | Sloan | 1980 | II | ***e1-as*** | *E2* | *E3-Mi* |
| PI 548694 | Peterson Jade | 1974 | II | *E1* | ***e2*** | ***e3-tr*** |
| PI 548537 | Marion | 1977 | II | *E1* | ***e2*** | *E3-Ha* |
| PI 548566 | Nebsoy | 1981 | II | *E1* | ***e2*** | *E3-Ha* |
| PI 539867 | LS301 | 1988 | III | ***e1-as*** | ***e2*** | *E3-Ha* |
| PI 548523 | Pella | 1980 | III | ***e1-as*** | ***e2*** | *E3-Ha* |
| PI 548691 | Agripro 26 | 1976 | III | ***e1-as*** | ***e2*** | *E3-Ha* |
| PI 556485 | AGRIPRO 27 | 1974 | III | ***e1-as*** | ***e2*** | *E3-Ha* |
| PI 556487 | AGRIPRO 25 | 1974 | III | ***e1-as*** | ***e2*** | *E3-Ha* |
| PI 556496 | A3001 | 1976 | III | ***e1-as*** | ***e2*** | *E3-Ha* |
| PI 556607 | AP 250 | 1981 | III | ***e1-as*** | ***e2*** | *E3-Ha* |
| PI 556660 | S30-31 | 1984 | III | ***e1-as*** | ***e2*** | *E3-Ha* |
| PI 556691 | L4106 | 1982 | III | ***e1-as*** | ***e2*** | *E3-Ha* |
| PI 556809 | AP 3023 | 1986 | III | ***e1-as*** | ***e2*** | *E3-Ha* |
| PI 556854 | AP 3132 | 1987 | III | ***e1-as*** | ***e2*** | *E3-Ha* |
| PI 556899 | S30-41 | 1991 | III | ***e1-as*** | ***e2*** | *E3-Ha* |
| PI 556469 | SEEDMAKERS I-E | 1973 | III | ***e1-as*** | *E2* | ***e3-tr*** |
| PI 556486 | PONTIAC | 1974 | III | ***e1-as*** | *E2* | ***e3-tr*** |
| PI 556538 | MATSOY | 1978 | III | ***e1-as*** | *E2* | ***e3-tr*** |
| PI 556539 | PL 723299L | 1978 | III | ***e1-as*** | *E2* | ***e3-tr*** |
| PI 556586 | S33-45 | 1987 | III | ***e1-as*** | *E2* | ***e3-tr*** |
| PI 556614 | 2981 | 1981 | III | ***e1-as*** | *E2* | ***e3-tr*** |
| PI 556870 | 9301 | 1989 | III | ***e1-as*** | *E2* | ***e3-tr*** |
| PI 556909 | 9302 | 1990 | III | ***e1-as*** | *E2* | ***e3-tr*** |
| PI 556910 | 9303 | 1990 | III | ***e1-as*** | *E2* | ***e3-tr*** |
| PI 559379 | 3311 | 1992 | III | ***e1-as*** | *E2* | ***e3-tr*** |
| PI 518667 | Harper 87 | 1988 | III | ***e1-as*** | *E2* | *E3-Ha* |
| PI 534645 | Resnik | 1988 | III | ***e1-as*** | *E2* | *E3-Ha* |
| PI 534647 | GR8836 | 1988 | III | ***e1-as*** | *E2* | *E3-Ha* |
| PI 534648 | GR8936 | 1988 | III | ***e1-as*** | *E2* | *E3-Ha* |
| PI 536635 | Sprite | 1982 | III | ***e1-as*** | *E2* | *E3-Ha* |
| PI 540551 | Hobbit | 1983 | III | ***e1-as*** | *E2* | *E3-Ha* |
| PI 542044 | Kunitz | 1992 | III | ***e1-as*** | *E2* | *E3-Ha* |
| PI 542061 | 9311 | 1994 | III | ***e1-as*** | *E2* | *E3-Ha* |
| PI 542062 | 9381 | 1994 | III | ***e1-as*** | *E2* | *E3-Ha* |
| PI 542071 | DSR-373 | 1994 | III | ***e1-as*** | *E2* | *E3-Ha* |
| PI 542709 | Hayes | 1992 | III | ***e1-as*** | *E2* | *E3-Ha* |
| PI 542711 | Edison | 1994 | III | ***e1-as*** | *E2* | *E3-Ha* |
| PI 546373 | Hobbit 87 | 1992 | III | ***e1-as*** | *E2* | *E3-Ha* |
| PI 546374 | Sprite 87 | 1992 | III | ***e1-as*** | *E2* | *E3-Ha* |
| PI 548522 | BSR 301 | 1980 | III | ***e1-as*** | *E2* | *E3-Ha* |
| PI 548525 | BSR 302 | 1982 | III | ***e1-as*** | *E2* | *E3-Ha* |
| PI 548542 | Cumberland | 1980 | III | ***e1-as*** | *E2* | *E3-Ha* |
| PI 548543 | Oakland | 1980 | III | ***e1-as*** | *E2* | *E3-Ha* |
| PI 548585 | Winchester | 1985 | III | ***e1-as*** | *E2* | *E3-Ha* |
| PI 548614 | Sherman | 1987 | III | ***e1-as*** | *E2* | *E3-Ha* |
| PI 548634 | Zane | 1985 | III | ***e1-as*** | *E2* | *E3-Ha* |
| PI 548635 | Chamberlain | 1988 | III | ***e1-as*** | *E2* | *E3-Ha* |
| PI 548684 | SRF 307B | 1974 | III | ***e1-as*** | *E2* | *E3-Ha* |
| PI 549098 | SS 390 | 1992 | III | ***e1-as*** | *E2* | *E3-Ha* |
| PI 550730 | 9392 | 1992 | III | ***e1-as*** | *E2* | *E3-Ha* |
| PI 550739 | A3242 | 1992 | III | ***e1-as*** | *E2* | *E3-Ha* |
| PI 556471 | XK-585 | 1974 | III | ***e1-as*** | *E2* | *E3-Ha* |
| PI 556473 | SRF 350 | 1974 | III | ***e1-as*** | *E2* | *E3-Ha* |
| PI 556476 | SRF 307P | 1974 | III | ***e1-as*** | *E2* | *E3-Ha* |
| PI 556505 | CLASSIC II | 1976 | III | ***e1-as*** | *E2* | *E3-Ha* |
| PI 556510 | A 3585 | 1977 | III | ***e1-as*** | *E2* | *E3-Ha* |
| PI 556511 | A3127 | 1977 | III | ***e1-as*** | *E2* | *E3-Ha* |
| PI 556524 | IKE | 1977 | III | ***e1-as*** | *E2* | *E3-Ha* |
| PI 556535 | MAX | 1978 | III | ***e1-as*** | *E2* | *E3-Ha* |
| PI 556542 | PL 709947 | 1978 | III | ***e1-as*** | *E2* | *E3-Ha* |
| PI 556556 | GT 1310 | 1979 | III | ***e1-as*** | *E2* | *E3-Ha* |
| PI 556568 | PL 72-3176L | 1980 | III | ***e1-as*** | *E2* | *E3-Ha* |
| PI 556570 | GUTWEIN 221 | 1980 | III | ***e1-as*** | *E2* | *E3-Ha* |
| PI 556572 | A3659 | 1980 | III | ***e1-as*** | *E2* | *E3-Ha* |
| PI 556573 | A3501 | 1988 | III | ***e1-as*** | *E2* | *E3-Ha* |
| PI 556577 | FFR 224 | 1980 | III | ***e1-as*** | *E2* | *E3-Ha* |
| PI 556578 | RIVERSIDE 2025 | 1980 | III | ***e1-as*** | *E2* | *E3-Ha* |
| PI 556579 | MAGNUM | 1980 | III | ***e1-as*** | *E2* | *E3-Ha* |
| PI 556592 | 3081 | 1980 | III | ***e1-as*** | *E2* | *E3-Ha* |
| PI 556594 | 3981 | 1980 | III | ***e1-as*** | *E2* | *E3-Ha* |
| PI 556609 | GT 1380 | 1981 | III | ***e1-as*** | *E2* | *E3-Ha* |
| PI 556610 | HP 3700 | 1981 | III | ***e1-as*** | *E2* | *E3-Ha* |
| PI 556617 | SRF 350P | 1981 | III | ***e1-as*** | *E2* | *E3-Ha* |
| PI 556621 | L4104 | 1981 | III | ***e1-as*** | *E2* | *E3-Ha* |
| PI 556630 | CS31 | 1981 | III | ***e1-as*** | *E2* | *E3-Ha* |
| PI 556632 | J-112 | 1982 | III | ***e1-as*** | *E2* | *E3-Ha* |
| PI 556642 | TS250 | 1982 | III | ***e1-as*** | *E2* | *E3-Ha* |
| PI 556668 | 3580 | 1983 | III | ***e1-as*** | *E2* | *E3-Ha* |
| PI 556671 | 3481 | 1983 | III | ***e1-as*** | *E2* | *E3-Ha* |
| PI 556687 | A3966 | 1984 | III | ***e1-as*** | *E2* | *E3-Ha* |
| PI 556707 | DSR-320 | 1983 | III | ***e1-as*** | *E2* | *E3-Ha* |
| PI 556709 | DSR-352 | 1983 | III | ***e1-as*** | *E2* | *E3-Ha* |
| PI 556714 | 71229-31008 | 1983 | III | ***e1-as*** | *E2* | *E3-Ha* |
| PI 556725 | S39-93 | 1985 | III | ***e1-as*** | *E2* | *E3-Ha* |
| PI 556730 | A3420 | 1985 | III | ***e1-as*** | *E2* | *E3-Ha* |
| PI 556741 | COKER 393 | 1985 | III | ***e1-as*** | *E2* | *E3-Ha* |
| PI 556745 | RIVERSIDE 2024 | 1985 | III | ***e1-as*** | *E2* | *E3-Ha* |
| PI 556748 | PRN-82 | 1985 | III | ***e1-as*** | *E2* | *E3-Ha* |
| PI 556766 | B335 | 1986 | III | ***e1-as*** | *E2* | *E3-Ha* |
| PI 556778 | A3427 | 1986 | III | ***e1-as*** | *E2* | *E3-Ha* |
| PI 556780 | A3803 | 1986 | III | ***e1-as*** | *E2* | *E3-Ha* |
| PI 556781 | A3307 | 1987 | III | ***e1-as*** | *E2* | *E3-Ha* |
| PI 556792 | DSR-317 | 1987 | III | ***e1-as*** | *E2* | *E3-Ha* |
| PI 556799 | 9361 | 1987 | III | ***e1-as*** | *E2* | *E3-Ha* |
| PI 556814 | A3733 | 1987 | III | ***e1-as*** | *E2* | *E3-Ha* |
| PI 556815 | A3511 | 1987 | III | ***e1-as*** | *E2* | *E3-Ha* |
| PI 556816 | A3205 | 1987 | III | ***e1-as*** | *E2* | *E3-Ha* |
| PI 556829 | HS 302 | 1987 | III | ***e1-as*** | *E2* | *E3-Ha* |
| PI 556830 | HS 321 | 1987 | III | ***e1-as*** | *E2* | *E3-Ha* |
| PI 556831 | HS 339 | 1988 | III | ***e1-as*** | *E2* | *E3-Ha* |
| PI 556832 | HS 348 | 1988 | III | ***e1-as*** | *E2* | *E3-Ha* |
| PI 556839 | 9331 | 1988 | III | ***e1-as*** | *E2* | *E3-Ha* |
| PI 556840 | 9391 | 1988 | III | ***e1-as*** | *E2* | *E3-Ha* |
| PI 556855 | AP 3773 | 1987 | III | ***e1-as*** | *E2* | *E3-Ha* |
| PI 556857 | A3935 | 1988 | III | ***e1-as*** | *E2* | *E3-Ha* |
| PI 556859 | A3415 | 1989 | III | ***e1-as*** | *E2* | *E3-Ha* |
| PI 556865 | S34-19 | 1988 | III | ***e1-as*** | *E2* | *E3-Ha* |
| PI 556866 | S36-36 | 1990 | III | ***e1-as*** | *E2* | *E3-Ha* |
| PI 556875 | 9402 | 1990 | III | ***e1-as*** | *E2* | *E3-Ha* |
| PI 556882 | AP 3977 | 1989 | III | ***e1-as*** | *E2* | *E3-Ha* |
| PI 556900 | S31-33 | 1991 | III | ***e1-as*** | *E2* | *E3-Ha* |
| PI 556911 | 9341 | 1991 | III | ***e1-as*** | *E2* | *E3-Ha* |
| PI 556928 | A3322 | 1991 | III | ***e1-as*** | *E2* | *E3-Ha* |
| PI 556931 | CX329 | 1991 | III | ***e1-as*** | *E2* | *E3-Ha* |
| PI 556933 | W 20 | 1993 | III | ***e1-as*** | *E2* | *E3-Ha* |
| PI 556996 | S33-32 | 1992 | III | ***e1-as*** | *E2* | *E3-Ha* |
| PI 556997 | S39-11 | 1992 | III | ***e1-as*** | *E2* | *E3-Ha* |
| PI 559399 | FFR 343 | 1994 | III | ***e1-as*** | *E2* | *E3-Ha* |
| PI 559400 | FFR 373 | 1994 | III | ***e1-as*** | *E2* | *E3-Ha* |
| PI 561209 | S35-35 | 1994 | III | ***e1-as*** | *E2* | *E3-Ha* |
| PI 561210 | S38-83 | 1994 | III | ***e1-as*** | *E2* | *E3-Ha* |
| PI 561583 | 9351 | 1994 | III | ***e1-as*** | *E2* | *E3-Ha* |
| PI 561585 | 9312 | 1994 | III | ***e1-as*** | *E2* | *E3-Ha* |
| PI 564718 | Thorne | 1995 | III | ***e1-as*** | *E2* | *E3-Ha* |
| PI 567902 | Charleston | 1995 | III | ***e1-as*** | *E2* | *E3-Ha* |
| PI 568245 | A3510 | 1995 | III | ***e1-as*** | *E2* | *E3-Ha* |
| PI 574534 | Piatt | 1995 | III | ***e1-as*** | *E2* | *E3-Ha* |
| PI 556526 | A3860 | 1977 | III | ***e1-as*** | *E2* | *E3-Mi* |
| PI 556764 | S39-99 | 1985 | III | ***e1-as*** | *E2* | *E3-Mi* |
| PI 548619 | Sparks | 1984 | IV | ***e1-as*** | ***e2*** | *E3-Ha* |
| PI 556625 | AP 350 | 1982 | IV | ***e1-as*** | ***e2*** | *E3-Ha* |
| PI 515961 | Pennyrile | 1988 | IV | ***e1-as*** | *E2* | *E3-Ha* |
| PI 518663 | Avery | 1988 | IV | ***e1-as*** | *E2* | *E3-Ha* |
| PI 518668 | TN 4-86 | 1988 | IV | ***e1-as*** | *E2* | *E3-Ha* |
| PI 525454 | Spencer | 1988 | IV | ***e1-as*** | *E2* | *E3-Ha* |
| PI 534646 | Flyer | 1988 | IV | ***e1-as*** | *E2* | *E3-Ha* |
| PI 540555 | Hamilton | 1992 | IV | ***e1-as*** | *E2* | *E3-Ha* |
| PI 543793 | Delsoy 4500 | 1992 | IV | ***e1-as*** | *E2* | *E3-Ha* |
| PI 543856 | Pixie | 1982 | IV | ***e1-as*** | *E2* | *E3-Ha* |
| PI 543934 | S48-84 | 1992 | IV | ***e1-as*** | *E2* | *E3-Ha* |
| PI 548517 | Bonus | 1973 | IV | ***e1-as*** | *E2* | *E3-Ha* |
| PI 548518 | Cutler 71 | 1973 | IV | ***e1-as*** | *E2* | *E3-Ha* |
| PI 548541 | Crawford | 1980 | IV | ***e1-as*** | *E2* | *E3-Ha* |
| PI 548549 | DeSoto | 1982 | IV | ***e1-as*** | *E2* | *E3-Ha* |
| PI 548555 | Douglas | 1982 | IV | ***e1-as*** | *E2* | *E3-Ha* |
| PI 548559 | Emerald | 1975 | IV | ***e1-as*** | *E2* | *E3-Ha* |
| PI 548636 | Regal | 1986 | IV | ***e1-as*** | *E2* | *E3-Ha* |
| PI 548679 | Mitchell | 1974 | IV | ***e1-as*** | *E2* | *E3-Ha* |
| PI 548682 | SRF 400 | 1973 | IV | ***e1-as*** | *E2* | *E3-Ha* |
| PI 548685 | SRF 450 | 1973 | IV | ***e1-as*** | *E2* | *E3-Ha* |
| PI 550731 | 9443 | 1992 | IV | ***e1-as*** | *E2* | *E3-Ha* |
| PI 556475 | SRF 425 | 1973 | IV | ***e1-as*** | *E2* | *E3-Ha* |
| PI 556528 | A4268 | 1977 | IV | ***e1-as*** | *E2* | *E3-Ha* |
| PI 556533 | RA-401 | 1978 | IV | ***e1-as*** | *E2* | *E3-Ha* |
| PI 556541 | GUTWEIN 421 | 1978 | IV | ***e1-as*** | *E2* | *E3-Ha* |
| PI 556546 | FFR 444 | 1978 | IV | ***e1-as*** | *E2* | *E3-Ha* |
| PI 556549 | S4055 | 1978 | IV | ***e1-as*** | *E2* | *E3-Ha* |
| PI 556550 | AP 40 | 1979 | IV | ***e1-as*** | *E2* | *E3-Ha* |
| PI 556551 | CO-OP 500 | 1979 | IV | ***e1-as*** | *E2* | *E3-Ha* |
| PI 556560 | MITCHELL 450 | 1979 | IV | ***e1-as*** | *E2* | *E3-Ha* |
| PI 556562 | VICTOR | 1979 | IV | ***e1-as*** | *E2* | *E3-Ha* |
| PI 556567 | SRF 450P | 1980 | IV | ***e1-as*** | *E2* | *E3-Ha* |
| PI 556576 | FFR 335 | 1980 | IV | ***e1-as*** | *E2* | *E3-Ha* |
| PI 556581 | DELTA | 1980 | IV | ***e1-as*** | *E2* | *E3-Ha* |
| PI 556589 | 4280 | 1980 | IV | ***e1-as*** | *E2* | *E3-Ha* |
| PI 556595 | STEVENS | 1980 | IV | ***e1-as*** | *E2* | *E3-Ha* |
| PI 556616 | GT 1440 | 1981 | IV | ***e1-as*** | *E2* | *E3-Ha* |
| PI 556618 | PL 707743 | 1981 | IV | ***e1-as*** | *E2* | *E3-Ha* |
| PI 556629 | CS45 | 1981 | IV | ***e1-as*** | *E2* | *E3-Ha* |
| PI 556631 | CS38 | 1982 | IV | ***e1-as*** | *E2* | *E3-Ha* |
| PI 556645 | S40-44 | 1982 | IV | ***e1-as*** | *E2* | *E3-Ha* |
| PI 556659 | S45-01 | 1985 | IV | ***e1-as*** | *E2* | *E3-Ha* |
| PI 556676 | HP 4800 | 1982 | IV | ***e1-as*** | *E2* | *E3-Ha* |
| PI 556680 | AP 420 | 1984 | IV | ***e1-as*** | *E2* | *E3-Ha* |
| PI 556701 | MITCHELL 410 | 1983 | IV | ***e1-as*** | *E2* | *E3-Ha* |
| PI 556702 | RA-403 | 1983 | IV | ***e1-as*** | *E2* | *E3-Ha* |
| PI 556723 | S42-40 | 1985 | IV | ***e1-as*** | *E2* | *E3-Ha* |
| PI 556759 | RA-451 | 1985 | IV | ***e1-as*** | *E2* | *E3-Ha* |
| PI 556760 | RA-405 | 1985 | IV | ***e1-as*** | *E2* | *E3-Ha* |
| PI 556762 | S42-30 | 1986 | IV | ***e1-as*** | *E2* | *E3-Ha* |
| PI 556784 | A4595 | 1986 | IV | ***e1-as*** | *E2* | *E3-Ha* |
| PI 556811 | AP 4321 | 1986 | IV | ***e1-as*** | *E2* | *E3-Ha* |
| PI 556841 | 9442 | 1988 | IV | ***e1-as*** | *E2* | *E3-Ha* |
| PI 556852 | A4393 | 1988 | IV | ***e1-as*** | *E2* | *E3-Ha* |
| PI 556860 | A4009 | 1989 | IV | ***e1-as*** | *E2* | *E3-Ha* |
| PI 556886 | SS 487 | 1990 | IV | ***e1-as*** | *E2* | *E3-Ha* |
| PI 556889 | CX458 | 1989 | IV | ***e1-as*** | *E2* | *E3-Ha* |
| PI 556901 | S42-50 | 1991 | IV | ***e1-as*** | *E2* | *E3-Ha* |
| PI 556905 | FFR 398 | 1992 | IV | ***e1-as*** | *E2* | *E3-Ha* |
| PI 556912 | 9411 | 1991 | IV | ***e1-as*** | *E2* | *E3-Ha* |
| PI 556913 | 9461 | 1991 | IV | ***e1-as*** | *E2* | *E3-Ha* |
| PI 556932 | CX469c | 1991 | IV | ***e1-as*** | *E2* | *E3-Ha* |
| PI 559931 | Corsica | 1994 | IV | ***e1-as*** | *E2* | *E3-Ha* |
| PI 559932 | Manokin | 1994 | IV | ***e1-as*** | *E2* | *E3-Ha* |
| PI 559933 | KS4390 | 1995 | IV | ***e1-as*** | *E2* | *E3-Ha* |
| PI 560206 | Delsoy 4210 | 1994 | IV | ***e1-as*** | *E2* | *E3-Ha* |
| PI 560207 | Delsoy 4710 | 1994 | IV | ***e1-as*** | *E2* | *E3-Ha* |
| PI 564525 | S46-44 | 1994 | IV | ***e1-as*** | *E2* | *E3-Ha* |
| PI 540877 | FFR 464 | 1992 | IV | ***e1-as*** | *E2* | *E3-Mi* |
| PI 556688 | A4997 | 1984 | IV | ***e1-as*** | *E2* | *E3-Mi* |
| PI 556731 | A4271 | 1986 | IV | ***e1-as*** | *E2* | *E3-Mi* |
| PI 556733 | 9471 | 1985 | IV | ***e1-as*** | *E2* | *E3-Mi* |
| PI 556734 | 9441 | 1985 | IV | ***e1-as*** | *E2* | *E3-Mi* |
| PI 556846 | S44-77 | 1987 | IV | ***e1-as*** | *E2* | *E3-Mi* |
| PI 540884 | S43-34 | 1992 | IV | ***e1-as*** | *E2* | *E3-Ha* |
| PI 556540 | BIG BOY | 1978 | IV | *E1* | ***e2*** | ***e3-tr*** |
| PI 536636 | Ripley | 1987 | IV | *E1* | ***e2*** | *E3-Ha* |
| PI 556566 | HOBSON | 1979 | IV | *E1* | ***e2*** | *E3-Ha* |
| PI 556569 | PL 70-10546D | 1980 | IV | *E1* | ***e2*** | *E3-Ha* |
| PI 553051 | Spry | 1994 | IV | *E1* | *E2* | *E3-Ha* |
| PI 556596 | RA-402 | 1980 | IV | *E1* | *E2* | *E3-Ha* |
| PI 556851 | A4906 | 1987 | IV | *E1* | *E2* | *E3-Ha* |
| PI 561191 | H4464 | 1995 | IV | *E1* | *E2* | *E3-Ha* |
| PI 583366 | Chesapeake | 1995 | IV | *E1* | *E2* | *E3-Ha* |
| PI 543794 | Delsoy 4900 | 1992 | IV | *E1* | *E2* | *E3-Mi* |
| PI 548604 | Pershing | 1985 | IV | *E1* | *E2* | *E3-Mi* |
| PI 556758 | RA-452 | 1985 | IV | *E1* | *E2* | *E3-Mi* |
| PI 568238 | FFR 500 | 1995 | V | *E1* | *E2* | ***e3-tr*** |
| PI 518664 | Hutcheson | 1988 | V | *E1* | *E2* | *E3-Ha* |
| PI 527701 | A5474 | 1980 | V | *E1* | *E2* | *E3-Ha* |
| PI 543795 | Hartwig | 1992 | V | *E1* | *E2* | *E3-Ha* |
| PI 548655 | Forrest | 1975 | V | *E1* | *E2* | *E3-Ha* |
| PI 548974 | Bedford | 1980 | V | *E1* | *E2* | *E3-Ha* |
| PI 548977 | Epps | 1985 | V | *E1* | *E2* | *E3-Ha* |
| PI 550732 | 9593 | 1992 | V | *E1* | *E2* | *E3-Ha* |
| PI 550735 | 9521 | 1994 | V | *E1* | *E2* | *E3-Ha* |
| PI 556506 | McNair 500 | 1977 | V | *E1* | *E2* | *E3-Ha* |
| PI 556559 | RA-480 | 1979 | V | *E1* | *E2* | *E3-Ha* |
| PI 556582 | DELTAPINE 345 | 1980 | V | *E1* | *E2* | *E3-Ha* |
| PI 556626 | AP 55 | 1982 | V | *E1* | *E2* | *E3-Ha* |
| PI 556635 | DELTAPINE 105 | 1982 | V | *E1* | *E2* | *E3-Ha* |
| PI 556664 | SHILOH | 1984 | V | *E1* | *E2* | *E3-Ha* |
| PI 556669 | 5482 | 1983 | V | *E1* | *E2* | *E3-Ha* |
| PI 556685 | A5308 | 1982 | V | *E1* | *E2* | *E3-Ha* |
| PI 556697 | TERRA-VIG 505 | 1983 | V | *E1* | *E2* | *E3-Ha* |
| PI 556700 | RA-580 | 1984 | V | *E1* | *E2* | *E3-Ha* |
| PI 556711 | FFR 560 | 1984 | V | *E1* | *E2* | *E3-Ha* |
| PI 556718 | HARTZ 5252 | 1984 | V | *E1* | *E2* | *E3-Ha* |
| PI 556719 | HARTZ 5370 | 1984 | V | *E1* | *E2* | *E3-Ha* |
| PI 556721 | HARTZ 5171 | 1984 | V | *E1* | *E2* | *E3-Ha* |
| PI 556732 | 9571 | 1985 | V | *E1* | *E2* | *E3-Ha* |
| PI 556742 | COKER 355 | 1985 | V | *E1* | *E2* | *E3-Ha* |
| PI 556743 | COKER 485 | 1985 | V | *E1* | *E2* | *E3-Ha* |
| PI 556752 | HT5203 | 1985 | V | *E1* | *E2* | *E3-Ha* |
| PI 556775 | 9531 | 1986 | V | *E1* | *E2* | *E3-Ha* |
| PI 556782 | A5149 | 1986 | V | *E1* | *E2* | *E3-Ha* |
| PI 556793 | TERRA-VIG 553 | 1986 | V | *E1* | *E2* | *E3-Ha* |
| PI 556800 | 9581 | 1987 | V | *E1* | *E2* | *E3-Ha* |
| PI 556820 | S59-19 | 1987 | V | *E1* | *E2* | *E3-Ha* |
| PI 556828 | DELTAPINE 675 | 1987 | V | *E1* | *E2* | *E3-Ha* |
| PI 556861 | A5403 | 1989 | V | *E1* | *E2* | *E3-Ha* |
| PI 556902 | 6925 | 1991 | V | *E1* | *E2* | *E3-Ha* |
| PI 556903 | 6955 | 1991 | V | *E1* | *E2* | *E3-Ha* |
| PI 556904 | 6995 | 1991 | V | *E1* | *E2* | *E3-Ha* |
| PI 556914 | 9582 | 1991 | V | *E1* | *E2* | *E3-Ha* |
| PI 556930 | A5979 | 1992 | V | *E1* | *E2* | *E3-Ha* |
| PI 560307 | S59-60 | 1994 | V | *E1* | *E2* | *E3-Ha* |
| PI 561218 | A5560 | 1994 | V | *E1* | *E2* | *E3-Ha* |
| PI 561219 | A5885 | 1994 | V | *E1* | *E2* | *E3-Ha* |
| PI 561576 | FFR 595 | 1994 | V | *E1* | *E2* | *E3-Ha* |
| PI 561578 | HSC 591 | 1994 | V | *E1* | *E2* | *E3-Ha* |
| PI 561599 | H5088 | 1994 | V | *E1* | *E2* | *E3-Ha* |
| PI 527702 | A5980 | 1985 | V | *E1* | *E2* | *E3-Mi* |
| PI 549099 | SS 516 | 1992 | V | *E1* | *E2* | *E3-Mi* |
| PI 550733 | 9551 | 1994 | V | *E1* | *E2* | *E3-Mi* |
| PI 550734 | 9583 | 1992 | V | *E1* | *E2* | *E3-Mi* |
| PI 556494 | RA-526 | 1975 | V | *E1* | *E2* | *E3-Mi* |
| PI 556527 | A5312 | 1977 | V | *E1* | *E2* | *E3-Mi* |
| PI 556529 | A5618 | 1978 | V | *E1* | *E2* | *E3-Mi* |
| PI 556587 | S53-34 | 1988 | V | *E1* | *E2* | *E3-Mi* |
| PI 556597 | A5939 | 1981 | V | *E1* | *E2* | *E3-Mi* |
| PI 556611 | RA-481 | 1981 | V | *E1* | *E2* | *E3-Mi* |
| PI 556670 | 9561 | 1983 | V | *E1* | *E2* | *E3-Mi* |
| PI 556704 | RA-502 | 1983 | V | *E1* | *E2* | *E3-Mi* |
| PI 556744 | COKER 425 | 1985 | V | *E1* | *E2* | *E3-Mi* |
| PI 556773 | 9591 | 1986 | V | *E1* | *E2* | *E3-Mi* |
| PI 556826 | FFR 561 | 1987 | V | *E1* | *E2* | *E3-Mi* |
| PI 556834 | HARTZ 5164 | 1988 | V | *E1* | *E2* | *E3-Mi* |
| PI 556876 | FFR 565 | 1988 | V | *E1* | *E2* | *E3-Mi* |
| PI 556915 | 9592 | 1991 | V | *E1* | *E2* | *E3-Mi* |
| PI 559934 | KS5292 | 1994 | V | *E1* | *E2* | *E3-Mi* |
| PI 561596 | H507 | 1994 | V | *E1* | *E2* | *E3-Mi* |
| PI 561597 | H5566 | 1994 | V | *E1* | *E2* | *E3-Mi* |
| PI 556491 | BRYSOY 9 | 1975 | VI | *E1* | *E2* | ***e3-tr*** |
| PI 542067 | FFR 646 | 1992 | VI | *E1* | *E2* | *E3-Ha* |
| PI 542982 | H639 | 1992 | VI | *E1* | *E2* | *E3-Ha* |
| PI 543935 | S64-23 | 1992 | VI | *E1* | *E2* | *E3-Ha* |
| PI 543936 | S61-89 | 1992 | VI | *E1* | *E2* | *E3-Ha* |
| PI 548834 | FFR 695 | 1992 | VI | *E1* | *E2* | *E3-Ha* |
| PI 548975 | Centennial | 1979 | VI | *E1* | *E2* | *E3-Ha* |
| PI 548981 | Leflore | 1985 | VI | *E1* | *E2* | *E3-Ha* |
| PI 550738 | A6961 | 1992 | VI | *E1* | *E2* | *E3-Ha* |
| PI 556480 | McNair 600 | 1974 | VI | *E1* | *E2* | *E3-Ha* |
| PI 556504 | LANCER | 1976 | VI | *E1* | *E2* | *E3-Ha* |
| PI 556514 | COKER 136 | 1973 | VI | *E1* | *E2* | *E3-Ha* |
| PI 556534 | RA-601 | 1978 | VI | *E1* | *E2* | *E3-Ha* |
| PI 556558 | RA-604 | 1979 | VI | *E1* | *E2* | *E3-Ha* |
| PI 556564 | COKER 156 | 1979 | VI | *E1* | *E2* | *E3-Ha* |
| PI 556612 | TERRA-VIG 606 | 1981 | VI | *E1* | *E2* | *E3-Ha* |
| PI 556620 | FFR 668 | 1981 | VI | *E1* | *E2* | *E3-Ha* |
| PI 556647 | RA-680 | 1982 | VI | *E1* | *E2* | *E3-Ha* |
| PI 556649 | CYPRESS 6000 | 1982 | VI | *E1* | *E2* | *E3-Ha* |
| PI 556656 | S69-96 | 1983 | VI | *E1* | *E2* | *E3-Ha* |
| PI 556690 | A6520 | 1984 | VI | *E1* | *E2* | *E3-Ha* |
| PI 556703 | RA-606 | 1983 | VI | *E1* | *E2* | *E3-Ha* |
| PI 556716 | GK-67 | 1985 | VI | *E1* | *E2* | *E3-Ha* |
| PI 556720 | HARTZ 6383 | 1984 | VI | *E1* | *E2* | *E3-Ha* |
| PI 556722 | SUMTER | 1984 | VI | *E1* | *E2* | *E3-Ha* |
| PI 556726 | S69-54 | 1985 | VI | *E1* | *E2* | *E3-Ha* |
| PI 556728 | A6242 | 1985 | VI | *E1* | *E2* | *E3-Ha* |
| PI 556738 | Bradley | 1984 | VI | *E1* | *E2* | *E3-Ha* |
| PI 556795 | TERRA-VIG 616 | 1986 | VI | *E1* | *E2* | *E3-Ha* |
| PI 556796 | SANALONA | 1986 | VI | *E1* | *E2* | *E3-Ha* |
| PI 556803 | HARTZ 6130 | 1986 | VI | *E1* | *E2* | *E3-Ha* |
| PI 556804 | HARTZ 6383R | 1986 | VI | *E1* | *E2* | *E3-Ha* |
| PI 556807 | DELTAPINE 566 | 1986 | VI | *E1* | *E2* | *E3-Ha* |
| PI 556824 | SPARTAN | 1987 | VI | *E1* | *E2* | *E3-Ha* |
| PI 556827 | COKER 686 | 1987 | VI | *E1* | *E2* | *E3-Ha* |
| PI 556835 | HARTZ 6385 | 1988 | VI | *E1* | *E2* | *E3-Ha* |
| PI 556842 | 9641 | 1988 | VI | *E1* | *E2* | *E3-Ha* |
| PI 556867 | S61-10 | 1988 | VI | *E1* | *E2* | *E3-Ha* |
| PI 556893 | HSC B2J | 1988 | VI | *E1* | *E2* | *E3-Ha* |
| PI 556894 | HSC BALDWIN | 1988 | VI | *E1* | *E2* | *E3-Ha* |
| PI 556907 | DELTAPINE 726 | 1990 | VI | *E1* | *E2* | *E3-Ha* |
| PI 556917 | HARTZ 6200 | 1990 | VI | *E1* | *E2* | *E3-Ha* |
| PI 556918 | HARTZ 6686 | 1990 | VI | *E1* | *E2* | *E3-Ha* |
| PI 556919 | HARTZ 6372 | 1991 | VI | *E1* | *E2* | *E3-Ha* |
| PI 559927 | DP 3627 | 1994 | VI | *E1* | *E2* | *E3-Ha* |
| PI 560300 | HSC 623 | 1994 | VI | *E1* | *E2* | *E3-Ha* |
| PI 564526 | S62-66 | 1994 | VI | *E1* | *E2* | *E3-Ha* |
| PI 564745 | H6397 | 1995 | VI | *E1* | *E2* | *E3-Ha* |
| PI 508266 | Young | 1985 | VI | *E1* | *E2* | *E3-Mi* |
| PI 515960 | Sharkey | 1989 | VI | *E1* | *E2* | *E3-Mi* |
| PI 527703 | A6381 | 1985 | VI | *E1* | *E2* | *E3-Mi* |
| PI 527704 | A6785 | 1987 | VI | *E1* | *E2* | *E3-Mi* |
| PI 542053 | HARTZ 922 | 1993 | VI | *E1* | *E2* | *E3-Mi* |
| PI 556479 | FFR 666 | 1974 | VI | *E1* | *E2* | *E3-Mi* |
| PI 556636 | DELTAPINE 506 | 1982 | VI | *E1* | *E2* | *E3-Mi* |
| PI 556663 | DELTAPINE 246 | 1984 | VI | *E1* | *E2* | *E3-Mi* |
| PI 556699 | HARTZ 930 | 1982 | VI | *E1* | *E2* | *E3-Mi* |
| PI 556770 | 9691 | 1986 | VI | *E1* | *E2* | *E3-Mi* |
| PI 556817 | SAMPSON | 1986 | VI | *E1* | *E2* | *E3-Mi* |
| PI 556858 | A6297 | 1988 | VI | *E1* | *E2* | *E3-Mi* |
| PI 564742 | H608 | 1995 | VI | *E1* | *E2* | *E3-Mi* |
| PI 538774 | HSC 721 | 1991 | VII | *E1* | *E2* | *E3-Ha* |
| PI 542972 | H7190 | 1992 | VII | *E1* | *E2* | *E3-Ha* |
| PI 543832 | Buckshot 723 | 1992 | VII | *E1* | *E2* | *E3-Ha* |
| PI 550736 | 9761 | 1992 | VII | *E1* | *E2* | *E3-Ha* |
| PI 556478 | FFR 777 | 1973 | VII | *E1* | *E2* | *E3-Ha* |
| PI 556516 | TERRA-VIG 708 | 1977 | VII | *E1* | *E2* | *E3-Ha* |
| PI 556536 | COKER 237 | 1978 | VII | *E1* | *E2* | *E3-Ha* |
| PI 556553 | AP 70 | 1979 | VII | *E1* | *E2* | *E3-Ha* |
| PI 556571 | WILSTAR 790 | 1979 | VII | *E1* | *E2* | *E3-Ha* |
| PI 556583 | McNair 710 | 1980 | VII | *E1* | *E2* | *E3-Ha* |
| PI 556608 | AP 71 | 1981 | VII | *E1* | *E2* | *E3-Ha* |
| PI 556654 | S72-60 | 1983 | VII | *E1* | *E2* | *E3-Ha* |
| PI 556661 | DELTAPINE 417 | 1983 | VII | *E1* | *E2* | *E3-Ha* |
| PI 556662 | DELTAPINE 497 | 1983 | VII | *E1* | *E2* | *E3-Ha* |
| PI 556686 | A7372 | 1982 | VII | *E1* | *E2* | *E3-Ha* |
| PI 556717 | HARTZ 7126 | 1984 | VII | *E1* | *E2* | *E3-Ha* |
| PI 556794 | TERRA-VIG 717 | 1986 | VII | *E1* | *E2* | *E3-Ha* |
| PI 556801 | 9751 | 1987 | VII | *E1* | *E2* | *E3-Ha* |
| PI 556813 | A7986 | 1986 | VII | *E1* | *E2* | *E3-Ha* |
| PI 556818 | STARR | 1986 | VII | *E1* | *E2* | *E3-Ha* |
| PI 556825 | COKER 627 | 1987 | VII | *E1* | *E2* | *E3-Ha* |
| PI 556833 | HARTZ 7110 | 1987 | VII | *E1* | *E2* | *E3-Ha* |
| PI 556862 | 6847 | 1988 | VII | *E1* | *E2* | *E3-Ha* |
| PI 556868 | S74-40 | 1988 | VII | *E1* | *E2* | *E3-Ha* |
| PI 556916 | 9711 | 1991 | VII | *E1* | *E2* | *E3-Ha* |
| PI 568239 | FFR 731 | 1995 | VII | *E1* | *E2* | *E3-Ha* |
| PI 570653 | HSC 741 | 1995 | VII | *E1* | *E2* | *E3-Ha* |
| PI 583367 | Pearl | 1995 | VII | *E1* | *E2* | *E3-Ha* |
| PI 556481 | McNair 800 | 1974 | VII | *E1* | *E2* | *E3-Mi* |
| PI 556623 | COKER 317 | 1981 | VII | *E1* | *E2* | *E3-Mi* |
| PI 556774 | 9791 | 1986 | VII | *E1* | *E2* | *E3-Mi* |
| PI 556847 | 6727 | 1987 | VII | *E1* | *E2* | *E3-Mi* |
| PI 508267 | Johnston | 1985 | VIII | *E1* | *E2* | *E3-Ha* |
| PI 538781 | DELTAPINE 878 | 1992 | VIII | *E1* | *E2* | *E3-Ha* |
| PI 543933 | S83-30 | 1992 | VIII | *E1* | *E2* | *E3-Ha* |
| PI 556515 | COKER 338 | 1976 | VIII | *E1* | *E2* | *E3-Ha* |
| PI 556537 | COKER 488 | 1978 | VIII | *E1* | *E2* | *E3-Ha* |
| PI 556806 | HARTZ 8112 | 1986 | VIII | *E1* | *E2* | *E3-Ha* |
| PI 561586 | 9831 | 1994 | VIII | *E1* | *E2* | *E3-Ha* |
| PI 561712 | H8558 | 1995 | VIII | *E1* | *E2* | *E3-Ha* |
| PI 564528 | DP 3818 | 1995 | VIII | *E1* | *E2* | *E3-Ha* |
| PI 556467 | Coker Hampton 266A | 1973 | VIII | *E1* | *E2* | *E3-Mi* |
| PI 556727 | COLLIER | 1985 | VIII | *E1* | *E2* | *E3-Mi* |
| PI 556848 | 6738 | 1988 | VIII | *E1* | *E2* | *E3-Mi* |
| PI 556805 | Hartz 9190 | 1986 | IX | *E1* | *E2* | *E3-Ha* |

**Table S5.** *E* genotypes of major maturity genes of Dow AgroSciences soybean lines

| **Dow ID** | **RM** | ***E1/e1-as*** | ***E2/e2*** | ***E3/e3-tr*** |
| --- | --- | --- | --- | --- |
| 116 | 00.8 | ***e1-nl*** | ***e2*** | ***e3-tr*** |
| 120 | 00.8 | ***e1-nl*** | ***e2*** | *E3-Ha* |
| 114 | 00.9 | ***e1-nl*** | ***e2*** | ***e3-tr*** |
| 117 | 00.9 | ***e1-nl*** | ***e2*** | *E3-Ha* |
| 122 | 00.9 | ***e1-nl*** | ***e2*** | ***e3-tr*** |
| 126 | 00.9 | ***e1-nl*** | ***e2*** | ***e3-tr*** |
| 103 | 0.1 | ***e1-nl*** | ***e2*** | ***e3-tr*** |
| 121 | 0.1 | ***e1-nl*** | ***e2*** | *E3-Ha* |
| 124 | 0.1 | ***e1-nl*** | ***e2*** | *E3-Ha* |
| 102 | 0.2 | ***e1-nl*** | ***e2*** | *E3-Ha* |
| 110 | 0.2 | ***e1-as*** | ***e2*** | *E3-Ha* |
| 127 | 0.3 | ***e1-as*** | ***e2*** | *E3-Ha* |
| 108 | 0.4 | ***e1-as*** | ***e2*** | ***e3-tr*** |
| 123 | 0.4 | ***e1-nl*** | *E2* | *E3-Ha* |
| 132 | 0.4 | ***e1-as*** | ***e2*** | ***e3-tr*** |
| 113 | 0.5 | ***e1-as*** | ***e2*** | ***e3-tr*** |
| 129 | 0.5 | ***e1-nl*** | *E2* | ***e3-tr*** |
| 97 | 0.5 | ***e1-as*** | ***e2*** | ***e3-tr*** |
| 125 | 0.6 | ***e1-as*** | ***e2*** | ***e3-tr*** |
| 104 | 0.7 | ***e1-nl*** | *E2* | ***e3-tr*** |
| 115 | 0.7 | ***e1-as*** | ***e2*** | ***e3-tr*** |
| 59 | 0.7 | ***e1-as*** | ***e2*** | ***e3-tr*** |
| 94 | 0.7 | ***e1-as*** | ***e2*** | *E3-Ha* |
| 74 | 0.7 | ***e1-as*** | ***e2*** | *E3-Ha* |
| 119 | 0.8 | ***e1-as*** | ***e2*** | ***e3-tr*** |
| 131 | 0.8 | ***e1-nl*** | *E2* | *E3-Ha* |
| 101 | 0.9 | ***e1-as*** | ***e2*** | *E3-Ha* |
| 105 | 0.9 | ***e1-as*** | ***e2*** | *E3-Ha* |
| 107 | 0.9 | ***e1-as*** | ***e2*** | *E3-Ha* |
| 109 | 0.9 | ***e1-as*** | ***e2*** | *E3-Ha* |
| 112 | 0.9 | ***e1-as*** | ***e2*** | *E3-Ha* |
| 87 | 0.9 | ***e1-as*** | ***e2*** | *E3-Ha* |
| 64 | 1 | ***e1-as*** | *E2* | ***e3-tr*** |
| 26 | 1.1 | ***e1-as*** | ***e2*** | ***e3-tr*** |
| 57 | 1.5 | ***e1-as*** | ***e2*** | ***e3-tr*** |
| 23 | 1.5 | ***e1-as*** | ***e2*** | *E3-Ha* |
| 48 | 1.6 | ***e1-as*** | *E2* | ***e3-tr*** |
| 93 | 1.6 | ***e1-as*** | *E2* | ***e3-tr*** |
| 55 | 1.7 | ***e1-as*** | *E2* | *E3-Ha* |
| 71 | 1.8 | ***e1-as*** | *E2* | ***e3-tr*** |
| 19 | 1.9 | ***e1-as*** | *E2* | ***e3-tr*** |
| 88 | 1.9 | ***e1-as*** | *E2* | ***e3-tr*** |
| 34 | 1.9 | ***e1-as*** | *E2* | *E3-Ha* |
| 86 | 2 | ***e1-as*** | *E2* | ***e3-tr*** |
| 6 | 2 | ***e1-as*** | *E2* | ***e3-tr*** |
| 13 | 2 | ***e1-as*** | *E2* | ***e3-tr*** |
| 24 | 2 | ***e1-as*** | *E2* | ***e3-tr*** |
| 37 | 2.1 | ***e1-as*** | *E2* | ***e3-tr*** |
| 100 | 2.2 | ***e1-as*** | *E2* | ***e3-tr*** |
| 11 | 2.2 | ***e1-as*** | *E2* | ***e3-tr*** |
| 16 | 2.2 | ***e1-as*** | *E2* | ***e3-tr*** |
| 31 | 2.2 | ***e1-as*** | *E2* | ***e3-tr*** |
| 62 | 2.2 | ***e1-as*** | *E2* | ***e3-tr*** |
| 9 | 2.3 | ***e1-as*** | *E2* | ***e3-tr*** |
| 72 | 2.3 | ***e1-as*** | *E2* | ***e3-tr*** |
| 81 | 2.3 | ***e1-as*** | *E2* | ***e3-tr*** |
| 99 | 2.3 | ***e1-as*** | *E2* | ***e3-tr*** |
| 89 | 2.4 | ***e1-as*** | *E2* | ***e3-tr*** |
| 98 | 2.4 | ***e1-as*** | *E2* | ***e3-tr*** |
| 84 | 2.4 | ***e1-as*** | *E2* | *E3-Ha* |
| 69 | 2.4 | ***e1-as*** | *E2* | *E3-Ha* |
| 4 | 2.5 | ***e1-as*** | *E2* | ***e3-tr*** |
| 61 | 2.5 | ***e1-as*** | *E2* | ***e3-tr*** |
| 27 | 2.6 | ***e1-as*** | *E2* | ***e3-tr*** |
| 35 | 2.6 | ***e1-as*** | *E2* | *E3-Ha* |
| 92 | 2.7 | ***e1-as*** | ***e2*** | *E3-Ha* |
| 7 | 2.7 | ***e1-as*** | *E2* | *E3-Ha* |
| 49 | 2.7 | ***e1-as*** | *E2* | *E3-Ha* |
| 58 | 2.7 | ***e1-as*** | *E2* | *E3-Ha* |
| 85 | 2.7 | ***e1-as*** | *E2* | *E3-Ha* |
| 40 | 2.8 | ***e1-as*** | *E2* | ***e3-tr*** |
| 43 | 2.8 | ***e1-as*** | *E2* | ***e3-tr*** |
| 42 | 2.8 | ***e1-as*** | *E2* | *E3-Ha* |
| 32 | 3 | ***e1-as*** | *E2* | *E3-Ha* |
| 51 | 3 | ***e1-as*** | *E2* | *E3-Ha* |
| 63 | 3 | ***e1-as*** | *E2* | *E3-Ha* |
| 36 | 3.1 | ***e1-as*** | *E2* | *E3-Ha* |
| 73 | 3.1 | ***e1-as*** | *E2* | *E3-Ha* |
| 14 | 3.2 | ***e1-as*** | *E2* | *E3-Ha* |
| 28 | 3.2 | ***e1-as*** | *E2* | *E3-Ha* |
| 29 | 3.2 | ***e1-as*** | *E2* | *E3-Ha* |
| 46 | 3.2 | ***e1-as*** | *E2* | *E3-Ha* |
| 33 | 3.4 | ***e1-as*** | *E2* | *E3-Ha* |
| 18 | 3.5 | ***e1-as*** | *E2* | *E3-Ha* |
| 30 | 3.5 | ***e1-as*** | *E2* | *E3-Ha* |
| 80 | 3.5 | ***e1-as*** | *E2* | *E3-Ha* |
| 82 | 3.5 | ***e1-as*** | *E2* | *E3-Ha* |
| 15 | 3.6 | ***e1-as*** | *E2* | *E3-Ha* |
| 25 | 3.6 | ***e1-as*** | *E2* | *E3-Ha* |
| 50 | 3.6 | ***e1-as*** | *E2* | *E3-Ha* |
| 96 | 3.6 | ***e1-as*** | *E2* | *E3-Ha* |
| 1 | 3.7 | ***e1-as*** | *E2* | *E3-Ha* |
| 66 | 3.7 | ***e1-as*** | *E2* | *E3-Ha* |
| 52 | 3.8 | ***e1-as*** | *E2* | *E3-Ha* |
| 65 | 3.8 | ***e1-as*** | *E2* | *E3-Ha* |
| 78 | 3.8 | ***e1-as*** | *E2* | *E3-Ha* |
| 95 | 3.8 | ***e1-as*** | *E2* | *E3-Ha* |
| 2 | 3.9 | ***e1-as*** | *E2* | *E3-Ha* |
| 3 | 4 | ***e1-as*** | *E2* | *E3-Ha* |
| 20 | 4 | ***e1-as*** | *E2* | *E3-Ha* |
| 60 | 4.1 | ***e1-as*** | *E2* | *E3-Ha* |
| 76 | 4.1 | ***e1-as*** | *E2* | *E3-Ha* |
| 38 | 4.2 | ***e1-as*** | *E2* | *E3-Ha* |
| 79 | 4.2 | ***e1-as*** | *E2* | *E3-Ha* |
| 8 | 4.3 | ***e1-as*** | *E2* | *E3-Ha* |
| 10 | 4.3 | ***e1-as*** | *E2* | *E3-Ha* |
| 39 | 4.3 | ***e1-as*** | *E2* | *E3-Ha* |
| 22 | 4.4 | ***e1-as*** | *E2* | *E3-Ha* |
| 47 | 4.4 | ***e1-as*** | *E2* | *E3-Ha* |
| 70 | 4.4 | ***e1-as*** | *E2* | *E3-Ha* |
| 68 | 4.5 | ***e1-as*** | *E2* | *E3-Ha* |
| 17 | 4.6 | ***e1-as*** | *E2* | *E3-Ha* |
| 41 | 4.6 | ***e1-as*** | *E2* | *E3-Ha* |
| 53 | 4.6 | ***e1-as*** | *E2* | *E3-Ha* |
| 83 | 4.6 | ***e1-as*** | *E2* | *E3-Ha* |
| 12 | 4.7 | ***e1-as*** | *E2* | *E3-Ha* |
| 54 | 4.7 | ***e1-as*** | *E2* | *E3-Ha* |
| 5 | 4.8 | ***e1-as*** | *E2* | *E3-Ha* |
| 77 | 4.8 | ***e1-as*** | *E2* | *E3-Ha* |
| 44 | 4.9 | ***e1-as*** | *E2* | *E3-Ha* |
| 56 | 4.9 | ***e1-as*** | *E2* | *E3-Ha* |
| 91 | 4.9 | ***e1-as*** | *E2* | *E3-Ha* |
| 67 | 4.9 | *E1* | *E2* | *E3-Ha* |

**Table S6.** Allele calls and maturity gene predictions from Wen, et al. dataset [29].

| alleles | A/G | C/T | G/A | A/C |
| --- | --- | --- | --- | --- |
| chrom | 6 | 10 | 19 | 19 |
| pos (Wm82.a1) | 19653985 | 44691998 | 47392861 | 47442735 |
|  |  |  |  |  |
| 133 | AA | CC | GG | AA |
| 134 | AA | CC | GG | AA |
| 135 | AA | CC | **e3** | AA |
| 136 | AA | **e2** | **e3** | AA |
| 137 | AA | CC | GG | AA |
| 138 | AA | CC | GG | AA |
| 139 | AA | CC | **e3** | AA |
| 140 | AA | **e2** | **e3** | AA |
| 141 | AA | **e2** | GG | AA |
| 142 | AA | CC | GG | AA |
| 143 | AA | **e2** | **e3** | AA |
| 144 | AA | CC | GG | AA |
| 145 | AA | CC | GG | AA |
| 146 | AA | CC | **e3** | AA |
| 147 | AA | CC | **e3** | AA |
| 148 | AA | CC | **e3** | AA |
| 149 | AA | CC | **e3** | AA |
| 150 | AA | CC | **e3** | AA |
| 151 | AA | CC | GG | AA |
| 152 | AA | CC | **e3** | AA |
| 153 | AA | CC | **e3** | AA |
| 154 | AA | CC | GG | AA |
| 155 | AA | CC | GG | AA |
| 156 | AA | CC | GG | AA |
| 157 | AA | CC | **e3** | AA |
| 158 | AA | CC | GG | AA |
| 159 | AA | CC | GG | AA |
| 160 | AA | CC | GG | AA |
| 161 | AA | CC | **e3** | AA |
| 162 | AA | CC | GG | AA |
| 163 | AA | **e2** | **e3** | AA |
| 164 | AA | **e2** | **e3** | AA |
| 165 | AA | CC | GG | AA |
| 166 | AA | CC | **e3** | AA |
| 167 | AA | CC | **e3** | AA |
| 168 | AA | CC | GG | AA |
| 169 | AA | CC | GG | AA |
| 170 | AA | CC | GG | AA |
| 171 | AA | CC | GG | AA |
| 172 | AA | CC | GG | AA |
| 173 | AA | CC | GG | AA |
| 174 | AA | **e2** | **e3** | AA |
| 175 | AA | CC | GG | AA |
| 176 | AA | **e2** | **e3** | AA |
| 177 | AA | CC | GG | AA |
| 178 | AA | CC | **e3** | AA |
| 179 | AA | CC | **e3** | AA |
| 180 | AA | CC | **e3** | AA |
| 181 | AA | CC | NN | AA |
| 182 | AA | CC | GG | AA |
| 183 | AA | CC | GG | AA |
| 184 | AA | CC | **e3** | AA |
| 185 | AA | CC | **e3** | AA |
| 186 | AA | CC | **e3** | AA |
| 187 | AA | CC | **e3** | AA |
| 188 | AA | CC | GG | AA |
| 189 | AA | CC | GG | AA |
| 190 | AA | CC | GG | AA |
| 191 | AA | CC | GG | AA |
| 192 | AA | CC | GG | AA |
| 193 | AA | CC | GG | AA |
| 194 | AA | CC | GG | AA |
| 195 | AA | CC | GG | AA |
| 196 | AA | **e2** | GG | AA |
| 197 | AA | CC | **e3** | AA |
| 198 | AA | CC | **e3** | AA |
| 199 | AA | CC | GG | AA |
| 200 | AA | CC | GG | AA |
| 201 | AA | CC | GG | AA |
| 202 | AA | CC | GG | AA |
| 203 | AA | CC | GG | AA |
| 204 | AA | CC | **e3** | AA |
| 205 | AA | CC | GG | AA |
| 206 | AA | CC | **e3** | AA |
| 207 | AA | CC | GA | AA |
| 208 | AA | CC | **e3** | AA |
| 209 | AA | CC | GG | AA |
| 210 | AA | CC | **e3** | AA |
| 211 | AA | CC | GA | AA |
| 212 | AA | CC | **e3** | AA |
| 213 | AA | CC | GA | AA |
| 214 | AA | CC | GG | AA |
| 215 | AA | CC | GG | AA |
| 216 | AA | CC | GG | AA |
| 217 | AA | CC | GG | AA |
| 218 | AA | CC | GG | AA |
| 219 | AA | CC | GG | AA |
| 220 | AA | CC | GA | AA |
| 221 | AA | CC | GG | AA |
| 222 | AA | CC | GG | AA |
| 223 | AA | CC | GG | AA |
| 224 | AA | CC | **e3** | AA |
| 225 | AA | **e2** | **e3** | AA |
| 226 | AA | CC | GG | AA |
| 227 | AA | CC | GG | AA |
| 228 | AA | CC | NN | AA |
| 229 | AA | CC | GG | AA |
| 230 | AA | CC | GG | AA |
| 231 | AA | CC | **e3** | AA |
| 232 | AA | CC | **e3** | AA |
| 233 | AA | **e2** | GG | AA |
| 234 | AA | **e2** | **e3** | AA |
| 235 | AA | CC | GG | AA |
| 236 | AA | **e2** | **e3** | AA |
| 237 | AA | CC | GG | AA |
| 238 | AA | CC | **e3** | AA |
| 239 | AA | CC | **e3** | AA |
| 240 | AA | CC | GG | AA |
| 241 | AA | CC | GG | AA |
| 242 | AA | CC | GG | AA |
| 243 | AA | CC | GG | AA |
| 244 | AA | CC | GG | AA |
| 245 | AA | CC | GG | AA |
| 246 | AA | CC | **e3** | AA |
| 247 | AA | CC | **e3** | AA |
| 248 | AA | CC | **e3** | AA |
| 249 | AA | CC | **e3** | AA |
| 250 | AA | CC | **e3** | AA |
| 251 | AA | CC | GG | AA |
| 252 | AA | CC | GG | AA |
| 253 | AA | CC | GG | AA |
| 254 | AA | CC | GG | AA |
| 255 | AA | CC | GG | AA |
| 256 | AA | CC | **e3** | AA |
| 257 | AA | CC | GG | AA |
| 258 | AA | CC | GA | AA |
| 259 | AA | CC | GG | AA |
| 260 | AA | CC | GG | AA |
| 261 | AA | CC | GG | AA |
| 262 | AA | CC | GG | AA |
| 263 | AA | **e2** | **e3** | AA |
| 264 | AA | CC | **e3** | AA |
| 265 | AA | CC | **e3** | AA |
| 266 | AA | CC | **e3** | AA |
| 267 | AA | CC | GG | AA |
| 268 | AA | CC | GG | AA |
| 269 | AA | CC | **e3** | AA |
| 270 | AA | CC | **e3** | AA |
| 271 | AA | CC | **e3** | AA |
| 272 | AA | CC | GG | AA |
| 273 | AA | CC | GG | AA |
| 274 | AA | CC | GG | AA |
| 275 | AA | **e2** | **e3** | AA |
| 276 | AA | CC | GG | AA |
| 277 | AA | CC | **e3** | AA |
| 278 | AA | CC | **e3** | AA |
| 279 | AA | CC | **e3** | AA |
| 280 | AA | CC | **e3** | AA |
| 281 | AA | CC | NN | AA |
| 282 | AA | CC | GA | AA |
| 283 | AA | CC | GG | AA |
| 284 | AA | CC | GG | AA |
| 285 | AA | CC | GG | AA |
| 286 | AA | CC | GG | AA |
| 287 | AA | CC | GG | AA |
| 288 | AA | CC | GG | AA |
| 289 | AA | CC | GG | AA |
| 290 | AA | CC | GG | AA |
| 291 | AA | CC | GG | AA |
| 292 | AA | CC | **e3** | AA |
| 293 | AA | CC | **e3** | AA |
| 294 | AA | CC | **e3** | AA |
| 295 | AA | CC | **e3** | AA |
| 296 | AA | CC | GG | AA |
| 297 | AA | CC | GG | AA |
| 298 | AA | CT | GA | AA |
| 299 | AA | CC | GG | AA |
| 300 | AA | CC | GG | AA |
| 301 | AA | CC | **e3** | AA |
| 302 | AA | CC | **e3** | AA |
| 303 | AA | CC | GG | AA |
| 304 | AA | CC | GG | AA |
| 305 | AA | CC | GG | AA |
| 306 | AA | CC | GG | AA |
| 307 | AA | CT | GA | AA |
| 308 | AA | CC | GG | AA |
| 309 | AA | CC | GG | AA |
| 310 | AA | CC | GG | AA |
| 311 | AA | CC | **e3** | AA |
| 312 | AA | CC | GG | AA |
| 313 | AA | CC | GG | AA |
| 314 | AA | CC | **e3** | AA |
| 315 | AA | CC | **e3** | AA |
| 316 | AA | CC | GG | AA |
| 317 | AA | CC | **e3** | AA |
| 318 | AA | CC | GG | AA |
| 319 | AA | CC | GG | AA |
| 320 | AA | CC | GG | AA |
| 321 | AA | CC | GG | AA |
| 322 | AA | CC | GG | AA |
| 323 | AA | CC | GG | AA |
| 324 | AA | CC | GG | AA |
| 325 | AA | CC | GG | AA |
| 326 | AA | CC | GG | AA |
| 327 | AA | CC | GG | AA |
| 328 | AA | CC | GG | AA |
| 329 | AA | CC | GG | AA |
| 330 | AA | CC | GG | AA |
| 331 | AA | CC | GG | AA |
| 332 | AA | CC | GG | AA |
| 333 | AA | CC | GG | AA |
| 334 | AA | **e2** | **e3** | AA |
| 335 | AA | CC | **e3** | AA |
| 336 | AA | **e2** | **e3** | AA |
| 337 | AA | **e2** | **e3** | AA |
| 338 | AA | **e2** | **e3** | AA |
| 339 | AA | CT | **e3** | AA |
| 340 | AA | **e2** | **e3** | AA |
| 341 | AA | **e2** | **e3** | AA |
| 342 | AA | **e2** | **e3** | AA |
| 343 | AA | **e2** | GA | AA |
| 344 | AA | CC | **e3** | AA |
| 345 | AA | CC | **e3** | AA |
| 346 | AA | CC | **e3** | AA |
| 347 | AA | CC | GG | AA |
| 348 | AA | **e2** | **e3** | AA |
| 349 | AA | CC | GG | AA |
| 350 | AA | **e2** | **e3** | AA |
| 351 | AA | CC | **e3** | AA |
| 352 | AA | CC | GG | AA |
| 353 | AA | CC | **e3** | AA |
| 354 | AA | CC | **e3** | AA |
| 355 | AA | CC | **e3** | AA |
| 356 | AA | CC | GG | AA |
| 357 | AA | CC | **e3** | AA |
| 358 | AA | CC | **e3** | AA |
| 359 | AA | CC | GG | AA |
| 360 | AA | CC | GA | AA |
| 361 | AA | CC | GG | AA |
| 362 | AA | **e2** | **e3** | AA |
| 363 | AA | **e2** | GA | AA |
| 364 | AA | CC | GG | AA |
| 365 | AA | CC | **e3** | AA |
| 366 | AA | CC | **e3** | AA |
| 367 | AA | CC | **e3** | AA |
| 368 | AA | CC | **e3** | AA |
| 369 | AA | CC | **e3** | AA |
| 370 | AA | CC | GA | AA |
| 371 | AA | CC | **e3** | AA |
| 372 | AA | CC | GG | AA |
| 373 | AA | CC | GG | AA |
| 374 | AA | CC | GG | AA |
| 375 | AA | CC | **e3** | AA |
| 376 | AA | CC | GG | AA |
| 377 | AA | CC | **e3** | AA |
| 378 | AA | CC | **e3** | AA |
| 379 | AA | CC | GG | AA |
| 380 | AA | CC | GG | AA |
| 381 | AA | CC | **e3** | AA |
| 382 | AA | CC | GG | AA |
| 383 | AA | **e2** | **e3** | AA |
| 384 | AA | CC | GG | AA |
| 385 | AA | CC | GG | AA |
| 386 | AA | CC | GG | AA |
| 387 | AA | CC | GG | AA |
| 388 | AA | CC | GG | AA |
| 389 | AA | CC | GG | AA |
| 390 | AA | CC | **e3** | AA |
| 391 | AA | CC | **e3** | AA |
| 392 | AA | CC | GG | AA |
| 393 | AA | CC | **e3** | AA |
| 394 | AA | CC | GG | AA |
| 395 | AA | CC | GG | AA |
| 396 | AA | **e2** | **e3** | AA |
| 397 | AA | CC | GG | AA |
| 398 | AA | CC | **e3** | AA |
| 399 | AA | CC | GG | AA |
| 400 | AA | CC | GG | AA |
| 401 | AA | CC | GG | AA |
| 402 | AA | CC | GG | AA |
| 403 | AA | CC | GG | AA |
| 404 | AA | CC | GG | AA |
| 405 | AA | CC | GG | AA |
| 406 | AA | CC | GG | AA |
| 407 | AA | CC | GG | AA |
| 408 | AA | CC | GG | AA |
| 409 | AA | **e2** | **e3** | AA |
| 410 | AA | CC | GG | AA |
| 411 | AA | CC | GG | AA |
| 412 | AA | CC | **e3** | AA |
| 413 | AA | CC | GG | AA |
| 414 | AA | CC | **e3** | AA |
| 415 | AA | CC | GG | AA |
| 416 | AA | CC | GA | AA |
| 417 | AA | CC | GG | AA |
| 418 | AA | CC | GG | AA |
| 419 | AA | **e2** | GG | AA |
| 420 | AA | CC | GG | AA |
| 421 | AA | CC | **e3** | AA |
| 422 | AA | CC | **e3** | AA |
| 423 | AA | CC | GG | AA |
| 424 | AA | CC | GG | AA |
| 425 | AA | CC | **e3** | AA |
| 426 | AA | CC | GA | AA |
| 427 | AA | CC | GG | AA |
| 428 | AA | CC | GG | AA |
| 429 | AA | CC | GG | AA |
| 430 | AA | CC | GG | AA |
| 431 | AA | CC | GG | AA |
| 432 | AA | CC | GG | AA |
| 433 | AA | CC | GG | AA |
| 434 | AA | CC | GG | AA |
| 435 | AA | CC | **e3** | AA |
| 436 | AA | CC | GG | AA |
| 437 | AA | CC | GA | AA |
| 438 | AA | CC | GG | AA |
| 439 | AA | CC | **e3** | AA |
| 440 | AA | CC | **e3** | AA |
| 441 | AA | CC | **e3** | AA |
| 442 | AA | CC | GG | AA |
| 443 | AA | CC | GG | AA |
| 444 | AA | CC | GG | AA |
| 445 | AA | CC | GG | AA |
| 446 | AA | CC | GG | AA |
| 447 | AA | CC | GG | AA |
| 448 | AA | CC | GG | AA |
| 449 | AA | CC | GG | AA |
| 450 | AA | CC | **e3** | AA |
| 451 | AA | CC | **e3** | AA |
| 452 | AA | CC | **e3** | AA |
| 453 | AA | CC | NN | AA |
| 454 | AA | CC | GG | AA |
| 455 | AA | CC | GG | AA |
| 456 | AA | **e2** | **e3** | AA |
| 457 | AA | CC | GG | AA |
| 458 | AA | **e2** | **e3** | AA |
| 459 | AA | CC | GG | AA |
| 460 | AA | CC | GG | AA |
| 461 | AA | CC | **e3** | AA |
| 462 | AA | CC | **e3** | AA |
| 463 | AA | CC | GG | AA |
| 464 | AA | CC | **e3** | AA |
| 465 | AA | CC | **e3** | AA |
| 466 | AA | CC | GG | AA |
| 467 | AA | CC | GG | AA |
| 468 | AA | CC | GG | AA |
| 469 | AA | CC | GG | AA |
| 470 | AA | CC | GG | AA |
| 471 | AA | CC | **e3** | AA |
| 472 | AA | CC | **e3** | AA |
| 473 | AA | CC | GG | AA |
| 474 | AA | CC | GG | AA |
| 475 | AA | CC | GG | AA |
| 476 | AA | CC | GG | AA |
| 477 | AA | CC | GG | AA |
| 478 | AA | CC | GG | AA |
| 479 | AA | CC | GG | AA |
| 480 | AA | CC | GA | AA |
| 481 | AA | CC | GG | AA |
| 482 | AA | CC | **e3** | AA |
| 483 | AA | CC | **e3** | AA |
| 484 | AA | CC | **e3** | AA |
| 485 | AA | CC | **e3** | AA |
| 486 | AA | CC | **e3** | AA |
| 487 | AA | CC | **e3** | AA |
| 488 | AA | CC | **e3** | AA |
| 489 | AA | CC | GG | AA |
| 490 | AA | CC | GA | AA |
| 491 | AA | CC | GA | AA |
| 492 | AA | CC | **e3** | AA |
| 493 | AA | CC | **e3** | AA |
| 494 | AA | CC | **e3** | AA |
| 495 | AA | CC | **e3** | AA |
| 496 | AA | CC | GA | AA |
| 497 | AA | CC | GG | AA |
| 498 | AA | CC | **e3** | AA |
| 499 | AA | CC | GG | AA |
| 500 | AA | CC | GG | AA |
| 501 | AA | CC | **e3** | AA |
| 502 | AA | CC | GG | AA |
| 503 | AA | CC | GG | AA |
| 504 | AA | CC | GG | AA |
| 505 | AA | CC | GG | AA |
| 506 | AA | **e2** | **e3** | AA |
| 507 | AA | CC | GG | AA |
| 508 | AA | CC | **e3** | AA |
| 509 | AA | CC | **e3** | AA |
| 510 | AA | CC | **e3** | AA |
| 511 | AA | CC | GG | AA |
| 512 | AA | CC | **e3** | AA |
| 513 | AA | CC | GG | AA |
| 514 | AA | CC | GG | AA |
| 515 | AA | CC | GG | AA |
| 516 | AA | CC | GG | AA |
| 517 | AA | CC | GG | AA |
| 518 | AA | CC | GG | AA |
| 519 | AA | CC | GG | AA |
| 520 | AA | CC | GG | AA |
| 521 | AA | CC | GG | AA |
| 522 | AA | CC | GG | AA |
| 523 | AA | CC | GG | AA |
| 524 | AA | CC | **e3** | AA |
| 525 | GA | **e2** | **e3** | AA |
| 526 | AA | **e2** | **e3** | AA |
| 527 | AA | **e2** | **e3** | AA |
| 528 | AA | CC | GG | AA |
| 529 | AA | CC | GG | AA |
| 530 | AA | CC | **e3** | AA |
| 531 | AA | CC | **e3** | AA |
| 532 | AA | CC | GA | AA |
| 533 | AA | CC | **e3** | AA |
| 534 | AA | CC | **e3** | AA |
| 535 | AA | CC | GG | AA |
| 536 | AA | CC | GG | AA |
| 537 | AA | CC | GG | AA |
| 538 | AA | CC | GG | AA |
| 539 | AA | CC | GG | AA |
| 540 | AA | CC | GA | AA |
| 541 | AA | CC | GG | AA |
| 542 | AA | CC | GG | AA |
| 543 | AA | CC | GG | AA |
| 544 | AA | CC | GG | AA |
| 545 | AA | CC | GG | AA |
| 546 | AA | **e2** | GG | AA |
| 547 | AA | CC | **e3** | AA |
| 548 | AA | CC | **e3** | AA |
| 549 | AA | CC | **e3** | AA |
| 550 | AA | CC | **e3** | AA |
| 551 | AA | CC | **e3** | AA |
| 552 | AA | CC | GG | AA |
| 553 | AA | CC | GG | AA |
| 554 | AA | CC | GA | AA |
| 555 | AA | CC | GG | AA |
| 556 | AA | CC | GG | AA |
| 557 | AA | CT | GA | AA |
| 558 | AA | **e2** | **e3** | AA |
| 559 | AA | CC | **e3** | AA |
| 560 | AA | CC | GG | AA |
| 561 | AA | CC | GG | AA |
| 562 | AA | CC | GG | AA |
| 563 | AA | CC | GG | AA |
| 564 | AA | CC | GA | AA |
| 565 | AA | CC | GG | AA |
| 566 | AA | CC | **e3** | AA |
| 567 | AA | CC | **e3** | AA |
| 568 | AA | CC | **e3** | AA |
| 569 | AA | CC | GG | AA |
| 570 | AA | CC | **e3** | AA |
| 571 | AA | CC | **e3** | AA |
| 572 | AA | CC | **e3** | AA |
| 573 | AA | CC | GG | AA |
| 574 | AA | CC | GG | AA |
| 575 | AA | CC | GA | AA |
| 576 | AA | CC | GG | AA |
| 577 | AA | CC | GG | AA |
| 578 | AA | CC | **e3** | AA |
| 579 | AA | CC | **e3** | AA |
| 580 | AA | CC | GG | AA |
| 581 | AA | CC | GG | AA |
| 582 | AA | CC | **e3** | AA |
| 583 | AA | CC | **e3** | AA |
| 584 | AA | CC | GG | AA |
| 585 | AA | CC | GG | AA |
| 586 | AA | CC | GG | AA |
| 587 | AA | CC | GG | AA |
| 588 | AA | CC | **e3** | AA |
| 589 | AA | CC | **e3** | AA |
| 590 | AA | CC | GG | AA |
| 591 | AA | CC | GG | AA |
| 592 | AA | CC | **e3** | AA |
| 593 | AA | CC | **e3** | AA |
| 594 | AA | CC | **e3** | AA |
| 595 | AA | CC | **e3** | AA |
| 596 | AA | CC | **e3** | AA |
| 597 | AA | CC | **e3** | AA |
| 598 | AA | CC | GG | AA |
| 599 | AA | CC | GG | AA |
| 600 | AA | CC | GG | AA |
| 601 | AA | CC | GG | AA |
| 602 | AA | CC | GG | AA |
| 603 | AA | CC | GG | AA |
| 604 | AA | CC | GG | AA |
| 605 | AA | CC | GG | AA |
| 606 | AA | CC | GG | AA |
| 607 | AA | CC | **e3** | AA |
| 608 | AA | CC | GG | AA |
| 609 | AA | CC | GG | AA |
| 610 | AA | CC | **e3** | AA |
| 611 | AA | CC | GG | AA |
| 612 | AA | CC | GG | AA |
| 613 | AA | CC | **e3** | AA |
| 614 | AA | CC | **e3** | AA |
| 615 | AA | CC | **e3** | AA |
| 616 | AA | CC | GG | AA |
| 617 | AA | CC | GG | AA |
| 618 | AA | CC | GG | AA |
| 619 | AA | CC | GG | AA |
| 620 | AA | CC | GG | AA |
| 621 | AA | CC | GG | AA |
| 622 | AA | CC | GG | AA |
| 623 | AA | CC | GG | AA |
| 624 | AA | CC | GG | AA |
| 625 | AA | CC | GG | AA |
| 626 | AA | CC | GG | AA |
| 627 | AA | CC | GG | AA |
| 628 | AA | CC | **e3** | AA |
| 629 | AA | CC | **e3** | AA |
| 630 | AA | CC | GG | AA |
| 631 | AA | CC | GG | AA |
| 632 | AA | CC | GG | AA |
| 633 | AA | CC | GG | AA |
| 634 | AA | CC | GG | AA |
| 635 | AA | CC | GG | AA |
| 636 | AA | CC | GG | AA |
| 637 | AA | CC | GG | AA |
| **638** |  |  |  |  |
| 639 | AA | CC | **e3** | AA |
| 640 | AA | CC | GG | AA |
| 641 | AA | CC | GG | AA |
| 642 | AA | CC | **e3** | AA |
| 643 | AA | CC | **e3** | AA |
| 644 | AA | CC | GG | AA |
| 645 | AA | CC | **e3** | AA |
| 646 | AA | CC | GG | AA |
| 647 | AA | CC | **e3** | AA |
| 648 | AA | CC | GG | AA |
| 649 | AA | CC | GG | AA |
| 650 | AA | CC | GG | AA |
| 651 | AA | CC | **e3** | AA |
| 652 | AA | CC | GG | AA |
| 653 | AA | CC | GG | AA |
| 654 | AA | CC | GG | AA |
| 655 | AA | **e2** | GG | AA |
| **656** |  |  |  |  |
| 657 | AA | CC | GG | AA |
| 658 | AA | CT | **e3** | AA |
| 659 | AA | **e2** | **e3** | AA |
| 660 | AA | **e2** | **e3** | AA |
| 661 | AA | CT | **e3** | AA |
| 662 | **E1** | **e2** | GG | CA |
| 663 | AA | CC | **e3** | AA |
| 664 | AA | CC | GG | AA |
| 665 | AA | CC | GG | AA |
| 666 | AA | CC | GG | AA |
| 667 | AA | CC | **e3** | AA |
| 668 | AA | CC | GG | AA |
| 669 | AA | CC | **e3** | AA |
| 670 | AA | CC | GG | AA |
| 671 | AA | CC | GG | AA |
| 672 | AA | CC | GG | AA |
| 673 | AA | CC | GG | AA |
| 674 | AA | CC | GG | AA |
| 675 | AA | CC | **e3** | AA |
| 676 | AA | CC | GG | AA |
| 677 | AA | CC | GG | AA |
| 678 | AA | CC | **e3** | AA |
| 679 | AA | CC | GG | AA |
| 680 | AA | CC | **e3** | AA |
| 681 | AA | CC | GG | AA |
| 682 | AA | CC | GG | AA |
| 683 | AA | CC | GG | AA |
| 684 | AA | CC | GG | AA |
| 685 | AA | CC | GG | AA |
| 686 | AA | CC | GG | AA |
| 687 | AA | CC | **e3** | AA |
| 688 | AA | CC | GG | AA |
| 689 | AA | CC | GG | AA |
| 690 | AA | CC | GG | AA |
| 691 | AA | CC | GG | AA |
| 692 | AA | CC | GG | AA |
| 693 | AA | CC | GG | AA |
| 694 | AA | CC | **e3** | AA |
| 695 | AA | CC | GG | AA |
| 696 | AA | CC | GG | AA |
| 697 | AA | CC | GG | AA |
| 698 | AA | CC | GG | AA |
| 699 | AA | CC | GG | AA |
| 700 | AA | CC | GG | AA |
| 701 | AA | CC | GG | AA |
| 702 | AA | CC | GG | AA |
| 703 | AA | CC | **e3** | AA |
| 704 | AA | CC | GG | AA |
| 705 | AA | CC | GG | AA |
| 706 | AA | CC | GG | AA |
| 707 | AA | CC | GG | AA |
| 708 | AA | CC | GG | AA |
| 709 | AA | CC | **e3** | AA |
| 710 | AA | CC | NN | AA |
| 711 | AA | CC | GG | AA |
| 712 | AA | CC | **e3** | AA |
| 713 | AA | CC | **e3** | AA |
| 714 | AA | CC | **e3** | AA |
| 715 | AA | CC | GG | AA |
| 716 | AA | CC | GG | AA |
| 717 | AA | CC | GG | AA |
| 718 | AA | CC | GG | AA |
| 719 | AA | CC | GG | AA |
| 720 | AA | CC | GG | AA |
| 721 | AA | CC | GG | AA |
| 722 | AA | CC | GA | AA |
| 723 | AA | CC | **e3** | AA |
| 724 | AA | CC | GG | AA |
| 725 | AA | CC | **e3** | AA |
| 726 | AA | CC | GG | AA |
| 727 | AA | CC | GG | AA |
| 728 | AA | CC | GG | AA |
| 729 | AA | CC | GG | AA |
| 730 | AA | CC | GG | AA |
| 731 | AA | CC | GG | AA |
| 732 | AA | CC | GG | AA |
| 733 | AA | CC | GG | AA |
| 734 | AA | CC | GG | AA |
| 735 | AA | CC | GG | AA |
| 736 | AA | CC | GG | AA |
| 737 | AA | **e2** | **e3** | AA |
| 738 | AA | CC | GG | AA |
| 739 | **E1** | **e2** | GG | AA |
| 740 | AA | **e2** | **e3** | AA |
| 741 | AA | CC | GG | AA |
| 742 | AA | CT | NN | AA |
| 743 | AA | CC | GG | AA |
| 744 | AA | **e2** | GG | AA |
| 745 | AA | CC | GG | AA |
| 746 | AA | CC | **e3** | AA |
| 747 | AA | CC | **e3** | AA |
| 748 | AA | CC | **e3** | AA |
| 749 | AA | CC | GG | AA |
| 750 | AA | CC | GG | AA |
| 751 | AA | CC | GG | AA |
| 752 | AA | CC | GG | AA |
| 753 | AA | CC | GG | AA |
| 754 | AA | CC | GG | AA |
| 755 | AA | CC | GG | AA |
| 756 | AA | **e2** | **e3** | AA |
| 757 | AA | **e2** | GG | AA |
| 758 | AA | CC | **e3** | AA |
| 759 | AA | CC | **e3** | AA |
| 760 | AA | CC | GG | AA |
| 761 | AA | CC | GG | AA |
| 762 | AA | CC | **e3** | AA |
| 763 | AA | CC | GG | AA |
| 764 | AA | CC | **e3** | AA |
| 765 | AA | **e2** | **e3** | AA |
| 766 | AA | CC | GG | AA |
| 767 | AA | CC | GG | AA |
| 768 | AA | CC | GG | AA |
| 769 | AA | CC | GG | AA |
| 770 | AA | CC | **e3** | AA |
| 771 | AA | CC | GG | AA |
| 772 | AA | CC | GG | AA |
| 773 | AA | CC | **e3** | AA |
| 774 | AA | CC | GG | AA |
| 775 | AA | CC | GG | AA |
| 776 | AA | CC | GG | AA |
| 777 | AA | CC | GG | AA |
| 778 | AA | **e2** | **e3** | AA |
| 779 | AA | CC | GG | AA |
| 780 | AA | CC | GG | AA |
| 781 | AA | CC | GG | AA |
| 782 | AA | CC | GG | AA |
| 783 | AA | CC | GG | AA |
| 784 | AA | CC | GG | AA |
| 785 | AA | CC | GG | AA |
| 786 | AA | CC | GG | AA |
| 787 | AA | CC | **e3** | AA |
| 788 | AA | CC | **e3** | AA |
| 789 | AA | CC | GG | AA |
| 790 | AA | CC | GG | AA |
| 791 | AA | CC | GG | AA |
| 792 | AA | CC | GG | AA |
| 793 | AA | CC | GG | AA |
| 794 | AA | CC | GG | AA |
| 795 | AA | CC | GG | AA |
| 796 | AA | **e2** | **e3** | AA |
| 797 | AA | CC | **e3** | AA |
| 798 | AA | CC | **e3** | AA |
| 799 | AA | CC | **e3** | AA |
| 800 | AA | CC | GG | AA |
| 801 | AA | CC | GG | AA |
| 802 | AA | CC | GG | AA |
| 803 | AA | CC | GG | AA |
| 804 | AA | CC | GG | AA |
| 805 | AA | CC | GG | AA |
| 806 | AA | CC | GG | AA |
| 807 | AA | CC | GG | AA |
| 808 | AA | NN | **e3** | AA |
| 809 | AA | **e2** | **e3** | AA |
| 810 | AA | **e2** | **e3** | AA |
| 811 | AA | CC | **e3** | AA |
| 812 | AA | CC | **e3** | AA |
| 813 | AA | CC | GG | AA |
| 814 | AA | CC | **e3** | AA |
| 815 | AA | CC | **e3** | AA |
| 816 | AA | CC | **e3** | AA |
| 817 | AA | CC | GG | AA |
| 818 | AA | CC | GG | AA |
| 819 | AA | CC | **e3** | AA |
| 820 | AA | CC | GG | AA |
| 821 | AA | CC | GG | AA |
| 822 | AA | CC | GG | AA |
| 823 | AA | CC | GG | AA |
| 824 | AA | CC | GG | AA |
| 825 | AA | CC | GG | AA |
| 826 | AA | CC | GG | AA |
| 827 | AA | CC | GG | AA |
| 828 | AA | CC | GG | AA |
| 829 | AA | **e2** | GG | AA |
| 830 | AA | CC | GG | AA |
| 831 | AA | CC | GG | AA |
| 832 | AA | CC | **e3** | AA |
| 833 | AA | CC | **e3** | AA |
| 834 | AA | CC | **e3** | AA |
| 835 | AA | CC | GG | AA |
| 836 | AA | CC | GG | AA |
| 837 | AA | CC | GG | AA |
| 838 | AA | CC | GG | AA |
| 839 | AA | CC | GG | AA |
| 840 | AA | CC | GG | AA |
| 841 | AA | CC | GG | AA |
| 842 | AA | CC | GG | AA |
| 843 | AA | **e2** | GG | AA |
| 844 | AA | **e2** | GG | AA |
| 845 | AA | CC | **e3** | AA |
| 846 | AA | CC | GG | AA |
| 847 | AA | CC | GG | AA |
| 848 | AA | CC | GG | AA |
| 849 | AA | CC | GG | AA |
| 850 | AA | CC | GG | AA |
| 851 | AA | CC | GG | AA |
| 852 | AA | CC | GG | AA |
| 853 | AA | CC | GG | AA |
| 854 | AA | CC | **e3** | AA |
| 855 | AA | CC | **e3** | AA |
| 856 | AA | CC | GG | AA |
| 857 | AA | CC | **e3** | AA |
| 858 | AA | CC | GG | AA |
| 859 | AA | CC | GG | AA |
| 860 | AA | CC | GG | AA |
| 861 | AA | CC | GG | AA |
| 862 | AA | CC | GG | AA |
| 863 | AA | CC | GG | AA |
| 864 | AA | CC | GG | AA |
| 865 | AA | CC | GG | AA |
| 866 | AA | CC | GG | AA |
| 867 | AA | CC | GG | AA |
| 868 | AA | CC | GG | AA |
| 869 | AA | CC | GG | AA |
| 870 | AA | CC | **e3** | AA |
| 871 | AA | CC | GG | AA |
| 872 | AA | CC | GG | AA |
| 873 | AA | CC | GG | AA |
| 874 | AA | CC | GG | AA |
| 875 | AA | CC | GG | AA |
| 876 | AA | CC | GG | AA |
| 877 | AA | CC | GG | AA |
| 878 | AA | CC | **e3** | AA |
| 879 | AA | **e2** | **e3** | AA |
| 880 | AA | CC | **e3** | AA |
| 881 | AA | CC | GG | AA |
| 882 | AA | CC | GG | AA |
| 883 | AA | CC | GG | AA |
| 884 | AA | CC | GG | AA |
| 885 | AA | CC | GG | AA |
| 886 | AA | CC | GG | AA |
| 887 | AA | CC | GG | AA |
| 888 | AA | NN | NN | AA |
| 889 | AA | CC | GG | AA |
| 890 | AA | CC | GG | AA |
| 891 | AA | CT | GG | AA |
| 892 | AA | **e2** | GG | AA |
| 893 | AA | CC | GG | AA |
| 894 | AA | **e2** | **e3** | AA |
| 895 | AA | **e2** | **e3** | AA |
| 896 | AA | **e2** | **e3** | AA |
| 897 | AA | **e2** | GA | AA |
| 898 | AA | **e2** | GA | AA |
| 899 | AA | **e2** | GG | AA |
| 900 | AA | **e2** | GG | AA |
| 901 | AA | **e2** | **e3** | AA |
| 902 | AA | **e2** | **e3** | AA |
| 903 | AA | **e2** | **e3** | AA |
| 904 | AA | NN | **e3** | AA |
| 905 | AA | CC | GG | AA |
| 906 | AA | CC | GG | AA |
| 907 | AA | **e2** | GG | AA |
| 908 | AA | CC | GG | AA |
| 909 | AA | **e2** | **e3** | AA |
| 910 | AA | **e2** | **e3** | AA |
| 911 | AA | **e2** | **e3** | AA |
| 912 | AA | **e2** | **e3** | AA |
| 913 | AA | CT | **e3** | AA |
| 914 | AA | CT | GA | NN |
| 915 | AA | CC | GA | CA |
| 916 | AA | NN | NN | AA |
| 917 | AA | **e2** | **e3** | **e3** |
| 918 | AA | **e2** | **e3** | **e3** |
| 919 | AA | **e2** | **e3** | AA |
| 920 | AA | **e2** | **e3** | AA |
| 921 | AA | **e2** | **e3** | AA |
| 922 | AA | **e2** | **e3** | AA |
| 923 | AA | **e2** | **e3** | AA |
| 924 | AA | **e2** | **e3** | AA |
| 925 | AA | **e2** | **e3** | AA |
| 926 | AA | NN | GA | AA |
| 927 | AA | CC | GG | AA |
| 928 | AA | CC | GG | AA |
| 929 | AA | CC | GG | AA |
| 930 | AA | CC | **e3** | AA |
| 931 | AA | CC | **e3** | AA |
| 932 | AA | **e2** | **e3** | AA |
| 933 | AA | CC | GG | AA |
| 934 | AA | CC | GG | AA |
| 935 | AA | **e2** | **e3** | AA |
| 936 | AA | CT | NN | AA |
| 937 | AA | **e2** | **e3** | AA |
| 938 | AA | CC | GG | AA |
| 939 | AA | CC | GG | AA |
| **940** |  |  |  |  |
| 941 | AA | CC | GG | AA |
| 942 | AA | CC | GA | AA |
| 943 | AA | CC | GG | AA |
| 944 | AA | CC | **e3** | AA |
| 945 | AA | CC | **e3** | AA |
| 946 | AA | CC | GG | AA |
| 947 | AA | **e2** | GG | AA |
| 948 | AA | CC | GG | AA |
| 949 | AA | CC | GG | AA |
| 950 | AA | CC | GG | AA |
| 951 | AA | CC | **e3** | **e3** |
| 952 | AA | CC | GG | AA |
| 953 | AA | CC | NN | AA |
| 954 | AA | CC | GA | AA |
| 955 | AA | CC | GG | AA |
| 956 | AA | CC | **e3** | AA |
| 957 | AA | CC | **e3** | AA |
| 958 | AA | CC | **e3** | AA |
| 959 | AA | CC | GG | AA |
| 960 | AA | CC | GG | AA |
| 961 | AA | CC | GG | AA |
| 962 | AA | CC | **e3** | AA |
| 963 | AA | CC | GG | AA |
| 964 | AA | NN | **e3** | **e3** |
| 965 | **E1** | **e2** | **e3** | AA |
| 966 | AA | **e2** | **e3** | AA |
| 967 | AA | **e2** | **e3** | AA |
| 968 | AA | **e2** | **e3** | AA |
| 969 | AA | **e2** | **e3** | AA |
| 970 | AA | **e2** | GG | AA |
| 971 | AA | **e2** | **e3** | AA |
| 972 | **E1** | **e2** | **e3** | AA |
| 973 | AA | **e2** | **e3** | AA |
| 974 | AA | **e2** | **e3** | AA |
| 975 | AA | **e2** | **e3** | AA |
| 976 | AA | NN | NN | AA |
| 977 | AA | CC | GG | AA |
| 978 | AA | CC | GG | AA |
| 979 | AA | CC | **e3** | AA |
| 980 | AA | CC | **e3** | AA |
| 981 | AA | CC | GG | AA |
| 982 | AA | **e2** | **e3** | AA |
| 983 | AA | CC | GG | AA |
| 984 | AA | CC | GG | AA |
| 985 | AA | CT | **e3** | AA |
| 986 | AA | CC | GG | AA |
| 987 | AA | CC | GG | AA |
| 988 | AA | CC | GG | AA |
| 989 | AA | CC | GG | AA |
| 990 | AA | CC | GG | AA |
| 991 | AA | CC | GG | AA |
| 992 | AA | CC | **e3** | AA |
| 993 | AA | CC | GG | AA |
| 994 | AA | CC | GG | AA |
| 995 | AA | CC | GG | AA |
| 996 | AA | CC | GG | AA |
| 997 | AA | CC | GG | AA |
| 998 | AA | CC | GG | AA |
| 999 | AA | CC | GG | AA |
| 1000 | AA | CC | GG | AA |
| 1001 | AA | CC | GG | AA |
| 1002 | AA | CC | GG | AA |
| 1003 | AA | CC | GA | AA |
| 1004 | AA | CC | **e3** | AA |
| 1005 | AA | CC | **e3** | AA |
| 1006 | AA | CC | **e3** | AA |
| 1007 | AA | CC | GG | AA |
| 1008 | AA | CC | GG | AA |
| 1009 | AA | CC | GA | AA |
| 1010 | AA | **e2** | **e3** | AA |
| 1011 | AA | CC | **e3** | AA |
| 1012 | AA | CC | **e3** | AA |
| 1013 | AA | CC | **e3** | AA |
| 1014 | AA | CC | **e3** | AA |
| 1015 | AA | CC | **e3** | AA |
| 1016 | AA | CC | GA | AA |
| 1017 | AA | CC | **e3** | AA |
| 1018 | AA | CC | **e3** | AA |
| 1019 | AA | CC | **e3** | AA |
| 1020 | AA | CC | GA | AA |
| 1021 | AA | CC | GG | AA |
| 1022 | AA | CC | GG | AA |
| 1023 | AA | CC | **e3** | AA |
| 1024 | AA | CC | **e3** | AA |
| 1025 | AA | CC | **e3** | AA |
| 1026 | AA | CC | **e3** | AA |
| 1027 | AA | CC | **e3** | AA |
| 1028 | AA | CC | GG | AA |
| 1029 | AA | CC | GG | AA |
| 1030 | AA | CC | GG | AA |
| 1031 | AA | CC | GG | AA |
| 1032 | AA | CC | GG | AA |
| 1033 | AA | CC | **e3** | AA |
| 1034 | AA | CC | **e3** | AA |
| 1035 | AA | **e2** | **e3** | AA |
| 1036 | AA | CT | **e3** | AA |
| 1037 | AA | CC | **e3** | AA |
| 1038 | AA | CC | **e3** | AA |
| 1039 | AA | CC | **e3** | AA |
| 1040 | AA | CC | GA | AA |
| 1041 | AA | CC | **e3** | AA |
| 1042 | AA | CC | **e3** | AA |
| 1043 | AA | CC | GG | AA |
| 1044 | AA | CC | **e3** | AA |
| 1045 | AA | CC | **e3** | AA |
| 1046 | AA | CC | **e3** | AA |
| 1047 | AA | CC | GG | AA |
| 1048 | AA | CC | GG | AA |
| 1049 | AA | CC | GG | AA |
| 1050 | AA | CC | GG | AA |
| 1051 | AA | CC | GG | AA |
| 1052 | AA | CC | GG | AA |
| 1053 | AA | CC | GG | AA |
| 1054 | AA | CC | NN | AA |
| 1055 | AA | CT | GG | AA |
| 1056 | AA | CC | GG | AA |
| 1057 | AA | CC | GG | AA |
| 1058 | AA | CT | **e3** | AA |
| 1059 | AA | CC | GG | AA |
| 1060 | AA | CC | GA | AA |
| 1061 | AA | CT | GG | AA |
| 1062 | AA | CC | GG | AA |
| 1063 | AA | CC | GG | AA |
| 1064 | AA | CC | **e3** | AA |
| 1065 | AA | CC | **e3** | AA |
| 1066 | AA | CC | GG | AA |
| 1067 | AA | CC | GA | AA |
| 1068 | AA | CC | GG | AA |
| 1069 | AA | CC | GG | AA |
| 1070 | AA | CT | **e3** | AA |
| 1071 | AA | CT | **e3** | AA |
| 1072 | AA | CC | **e3** | AA |
| 1073 | AA | CT | GA | AA |
| 1074 | AA | CC | GA | AA |
| 1075 | AA | CC | GG | AA |
| 1076 | AA | CC | **e3** | AA |
| 1077 | AA | CC | **e3** | AA |
| 1078 | AA | CC | GA | AA |
| 1079 | AA | CC | GG | AA |
| 1080 | AA | CC | GG | AA |
| 1081 | AA | CC | GG | AA |
| 1082 | AA | NN | NN | NN |
| 1083 | AA | CC | GG | AA |
| 1084 | AA | CC | GG | AA |
| 1085 | AA | CC | GG | AA |
| 1086 | AA | CC | GG | AA |
| 1087 | AA | CC | GG | AA |
| 1088 | AA | CC | GG | AA |
| 1089 | AA | CC | GG | NN |
| 1090 | AA | CC | GG | AA |
| 1091 | AA | CC | GG | AA |
| 1092 | AA | CC | GG | AA |
| 1093 | AA | CC | GG | AA |
| 1094 | AA | CC | GG | AA |
| 1095 | AA | CC | GG | AA |
| 1096 | AA | CC | GG | AA |
| 1097 | AA | CC | GG | AA |
| 1098 | AA | CC | GG | AA |
| 1099 | AA | CC | GG | AA |
| 1100 | AA | CC | GG | AA |
| 1101 | AA | CC | NN | AA |
| 1102 | AA | CC | GG | AA |
| 1103 | AA | CC | **e3** | AA |
| 1104 | AA | **e2** | **e3** | AA |
| 1105 | AA | CC | GG | AA |
| 1106 | AA | CT | GA | AA |
| 1107 | AA | CC | **e3** | AA |
| 1108 | AA | CC | **e3** | AA |
| 1109 | AA | CC | **e3** | AA |
| 1110 | AA | CC | GG | AA |
| 1111 | AA | NN | **e3** | AA |
| 1112 | AA | **e2** | **e3** | AA |
| 1113 | AA | CC | **e3** | AA |
| 1114 | AA | CC | GG | AA |
| 1115 | AA | CT | GA | AA |
| 1116 | AA | CC | GG | AA |
| 1117 | AA | **e2** | **e3** | AA |
| 1118 | AA | CC | GG | AA |
| 1119 | AA | **e2** | GA | AA |
| 1120 | AA | CC | GG | AA |
| 1121 | AA | CC | GG | AA |
| 1122 | AA | NN | GG | AA |
| 1123 | AA | **e2** | **e3** | AA |
| 1124 | AA | CC | GG | AA |
| 1125 | AA | CC | GG | AA |
| 1126 | AA | CC | GG | AA |
| 1127 | AA | CC | GG | AA |
| 1128 | AA | CC | GG | AA |
| 1129 | AA | CC | GG | AA |
| 1130 | AA | CC | GG | AA |
| 1131 | AA | CC | GG | AA |
| 1132 | AA | CC | GG | AA |
| 1133 | AA | CC | GG | AA |
| 1134 | AA | CC | GG | AA |
| 1135 | AA | CC | GG | AA |
| 1136 | AA | CC | GG | AA |
| 1137 | AA | CC | GG | AA |
| 1138 | AA | CC | GG | AA |
| 1139 | AA | CC | GG | AA |
| 1140 | AA | CC | GG | AA |
| 1141 | AA | CC | GG | AA |
| 1142 | AA | CC | GG | AA |
| 1143 | AA | **e2** | GG | NN |
| 1144 | AA | NN | GG | AA |
| 1145 | AA | CC | GA | AA |
| 1146 | AA | CC | **e3** | AA |
| 1147 | AA | CC | **e3** | AA |
| 1148 | AA | CC | GA | AA |
| 1149 | AA | CC | **e3** | AA |
| 1150 | AA | CC | GG | AA |
| 1151 | AA | CC | GG | AA |
| 1152 | AA | CC | **e3** | AA |
| 1153 | AA | CC | GG | AA |
| 1154 | AA | CC | **e3** | AA |
| 1155 | AA | CC | GG | AA |
| 1156 | AA | CC | GG | AA |
| 1157 | AA | CC | GG | AA |
| 1158 | AA | CC | GG | AA |
| 1159 | AA | CC | GA | AA |
| 1160 | AA | CC | GG | AA |
| 1161 | AA | CC | GG | AA |
| 1162 | AA | CC | GG | AA |
| 1163 | AA | CC | GG | AA |
| 1164 | AA | CT | GG | CA |
| 1165 | AA | CC | GG | NN |
| 1166 | AA | CT | GG | NN |
| 1167 | AA | CC | GG | CA |
| 1168 | AA | CC | GG | AA |
| 1169 | AA | CC | GG | AA |
| 1170 | AA | **e2** | GG | **e3** |
| 1171 | AA | CT | GG | NN |
| 1172 | AA | CC | GG | AA |
| 1173 | AA | CC | GG | AA |
| 1174 | AA | **e2** | **e3** | AA |
| 1175 | AA | CC | **e3** | AA |
| 1176 | AA | CC | GG | AA |
| 1177 | AA | CC | GG | AA |
| 1178 | AA | CC | **e3** | AA |
| 1179 | AA | CC | GG | AA |
| 1180 | NN | **e2** | **e3** | AA |
| 1181 | AA | CT | GG | AA |
| 1182 | AA | CC | GG | AA |
| 1183 | AA | CC | GG | **e3** |
| 1184 | AA | **e2** | GG | **e3** |
| 2012-1 | AA | CT | **e3** | AA |
| 2012-10 | AA | CC | GG | AA |
| 2012-100 | AA | CC | **e3** | AA |
| 2012-101 | AA | CC | GG | AA |
| 2012-102 | AA | CC | GG | AA |
| 2012-103 | AA | CC | **e3** | AA |
| 2012-104 | AA | CC | GG | AA |
| 2012-105 | AA | CC | GA | AA |
| 2012-106 | AA | CC | **e3** | AA |
| 2012-107 | AA | CC | GG | AA |
| 2012-108 | AA | CC | GG | AA |
| 2012-109 | AA | CC | GG | AA |
| 2012-11 | AA | CT | GA | AA |
| 2012-110 | AA | CC | **e3** | AA |
| 2012-111 | AA | CC | GG | AA |
| 2012-112 | AA | **e2** | **e3** | AA |
| 2012-113 | AA | CC | GG | AA |
| 2012-114 | AA | CC | GA | AA |
| 2012-115 | AA | CC | GG | AA |
| 2012-116 | AA | CC | **e3** | AA |
| 2012-117 | AA | CC | GG | AA |
| 2012-118 | AA | CC | GG | AA |
| 2012-119 | AA | CC | GG | AA |
| 2012-12 | AA | CC | GG | AA |
| 2012-120 | AA | CC | GG | AA |
| 2012-121 | AA | CC | GG | AA |
| 2012-122 | AA | CC | **e3** | AA |
| 2012-123 | AA | **e2** | **e3** | AA |
| 2012-124 | AA | CC | GG | AA |
| 2012-125 | AA | **e2** | **e3** | AA |
| 2012-126 | AA | CC | GG | AA |
| 2012-127 | AA | CC | **e3** | AA |
| 2012-128 | AA | CC | GG | AA |
| 2012-129 | AA | CC | **e3** | AA |
| 2012-13 | AA | CC | **e3** | AA |
| 2012-130 | AA | CC | **e3** | AA |
| 2012-131 | AA | CC | GG | AA |
| 2012-132 | AA | CC | **e3** | AA |
| 2012-133 | AA | CC | GG | AA |
| 2012-134 | AA | CC | **e3** | AA |
| 2012-135 | AA | CC | **e3** | AA |
| 2012-136 | AA | CC | GG | AA |
| 2012-137 | AA | CC | **e3** | AA |
| 2012-138 | AA | CC | **e3** | AA |
| 2012-139 | AA | CC | **e3** | AA |
| 2012-14 | AA | CC | **e3** | AA |
| 2012-140 | AA | CC | GG | AA |
| 2012-141 | AA | CC | **e3** | AA |
| 2012-142 | AA | CC | **e3** | AA |
| 2012-143 | AA | CC | **e3** | AA |
| 2012-144 | AA | CC | **e3** | AA |
| 2012-145 | AA | CC | GG | AA |
| 2012-146 | AA | CC | GG | AA |
| 2012-147 | AA | CC | **e3** | AA |
| 2012-148 | AA | CC | GG | AA |
| 2012-149 | AA | CC | GG | AA |
| 2012-15 | AA | **e2** | GG | AA |
| 2012-150 | AA | CC | GG | AA |
| 2012-151 | AA | CC | GG | AA |
| 2012-152 | AA | **e2** | **e3** | AA |
| 2012-153 | AA | **e2** | **e3** | AA |
| 2012-154 | AA | **e2** | **e3** | AA |
| 2012-155 | AA | CC | GG | AA |
| 2012-156 | AA | CC | GA | AA |
| 2012-157 | AA | CC | **e3** | AA |
| 2012-158 | AA | CC | GG | AA |
| 2012-159 | AA | CC | GG | AA |
| 2012-16 | AA | CC | GG | AA |
| 2012-160 | AA | CC | GG | AA |
| 2012-161 | AA | CC | GG | AA |
| 2012-162 | AA | CC | GG | AA |
| 2012-163 | AA | CC | **e3** | AA |
| 2012-164 | AA | CC | **e3** | AA |
| 2012-165 | AA | CC | **e3** | AA |
| 2012-166 | AA | CC | GG | AA |
| 2012-167 | AA | **e2** | GA | AA |
| 2012-168 | AA | CC | GG | AA |
| 2012-169 | AA | CC | GG | AA |
| 2012-17 | AA | CC | GG | AA |
| 2012-170 | AA | CC | **e3** | AA |
| 2012-171 | AA | **e2** | **e3** | AA |
| 2012-172 | AA | **e2** | **e3** | AA |
| 2012-173 | AA | **e2** | **e3** | AA |
| 2012-174 | AA | CC | GG | AA |
| 2012-175 | AA | CC | GG | AA |
| 2012-176 | AA | CC | GG | AA |
| 2012-177 | AA | CC | GG | AA |
| 2012-178 | AA | CC | GG | AA |
| 2012-179 |  |  |  |  |
| 2012-18 | AA | **e2** | **e3** | AA |
| 2012-180 | AA | CC | GG | AA |
| 2012-181 | AA | CC | GG | AA |
| 2012-182 | AA | CC | **e3** | AA |
| 2012-183 | AA | CC | GG | AA |
| 2012-184 | AA | CC | GG | AA |
| 2012-185 | AA | CC | GG | AA |
| 2012-186 | AA | CC | **e3** | AA |
| 2012-19 | AA | **e2** | **e3** | AA |
| 2012-2 | **E1** | **e2** | GG | AA |
| 2012-20 | AA | **e2** | **e3** | AA |
| 2012-21 | AA | CC | GG | AA |
| 2012-22 | AA | CC | GG | AA |
| 2012-23 | AA | CT | **e3** | AA |
| 2012-24 | AA | CC | **e3** | AA |
| 2012-25 | AA | CC | GG | AA |
| 2012-26 | AA | CC | GA | AA |
| 2012-27 | AA | CC | GA | AA |
| 2012-28 | AA | CC | GA | AA |
| 2012-29 | AA | CC | GA | AA |
| 2012-3 | NN | CC | GG | AA |
| 2012-30 | AA | **e2** | **e3** | AA |
| 2012-31 | AA | **e2** | **e3** | AA |
| 2012-32 | AA | CC | **e3** | AA |
| 2012-33 | AA | CC | **e3** | AA |
| 2012-34 | AA | CC | GG | AA |
| 2012-35 | AA | CC | GA | CA |
| 2012-36 | AA | CC | GA | CA |
| 2012-37 | AA | CT | **e3** | AA |
| 2012-38 | AA | CC | **e3** | AA |
| 2012-39 | AA | **e2** | **e3** | AA |
| 2012-4 | AA | CT | GG | NN |
| 2012-40 | AA | **e2** | **e3** | AA |
| 2012-41 | AA | **e2** | **e3** | AA |
| 2012-42 | AA | **e2** | **e3** | AA |
| 2012-43 | AA | **e2** | **e3** | AA |
| 2012-44 | AA | CC | GG | AA |
| 2012-45 | AA | CC | GG | AA |
| 2012-46 | AA | CC | GG | AA |
| 2012-47 | AA | CC | GG | AA |
| 2012-48 | AA | CC | GG | AA |
| 2012-49 | AA | CC | GG | AA |
| 2012-5 | AA | CC | GG | **e3** |
| 2012-50 | AA | CC | GG | AA |
| 2012-51 | AA | CC | GG | AA |
| 2012-52 | AA | CC | GG | AA |
| 2012-53 | AA | CC | GG | AA |
| 2012-54 | AA | CC | GG | AA |
| 2012-55 | AA | CC | GG | AA |
| 2012-56 | AA | **e2** | GG | AA |
| 2012-57 | AA | CC | GG | AA |
| 2012-58 | AA | **e2** | GG | AA |
| 2012-59 | AA | CC | GG | AA |
| 2012-6 | AA | CC | GA | AA |
| 2012-60 | AA | CC | GG | AA |
| 2012-61 | AA | CC | GG | AA |
| 2012-62 | AA | CC | GG | AA |
| 2012-63 | AA | CC | GG | AA |
| 2012-64 | AA | CC | GG | AA |
| 2012-65 | AA | CC | GG | AA |
| 2012-66 | AA | CC | GG | AA |
| 2012-67 | AA | CC | GG | AA |
| 2012-68 | AA | CC | **e3** | AA |
| 2012-69 | AA | CC | **e3** | AA |
| 2012-7 | AA | CC | **e3** | AA |
| 2012-70 | AA | CC | **e3** | AA |
| 2012-71 | AA | CC | GG | AA |
| 2012-72 | AA | CC | **e3** | AA |
| 2012-73 | AA | CC | **e3** | AA |
| 2012-74 |  |  |  |  |
| 2012-75 | AA | CC | GG | AA |
| 2012-76 | AA | CC | GG | AA |
| 2012-77 |  |  |  |  |
| 2012-78 |  |  |  |  |
| 2012-79 | AA | CC | **e3** | NN |
| 2012-8 | AA | CC | GG | AA |
| 2012-80 | AA | CC | NN | **e3** |
| 2012-81 | **E1** | **e2** | GG | **e3** |
| 2012-82 | AA | CC | NN | **e3** |
| 2012-83 |  |  |  |  |
| 2012-84 |  |  |  |  |
| 2012-85 | AA | CC | GG | AA |
| 2012-86 | AA | CC | GG | AA |
| 2012-87 | NN | NN | GG | **e3** |
| 2012-88 | AA | CC | **e3** | AA |
| 2012-89 | AA | CC | GG | AA |
| 2012-9 | AA | CC | GG | AA |
| 2012-90 |  |  |  |  |
| 2012-91 | AA | CC | GG | AA |
| 2012-92 | AA | CC | **e3** | AA |
| 2012-93 | AA | CC | GG | AA |
| 2012-94 | AA | CC | GG | AA |
| 2012-95 | AA | CC | **e3** | AA |
| 2012-96 |  |  |  |  |
| 2012-97 | AA | CC | **e3** | AA |
| 2012-98 | AA | CC | **e3** | AA |
| 2012-99 | AA | CC | GG | AA |

*
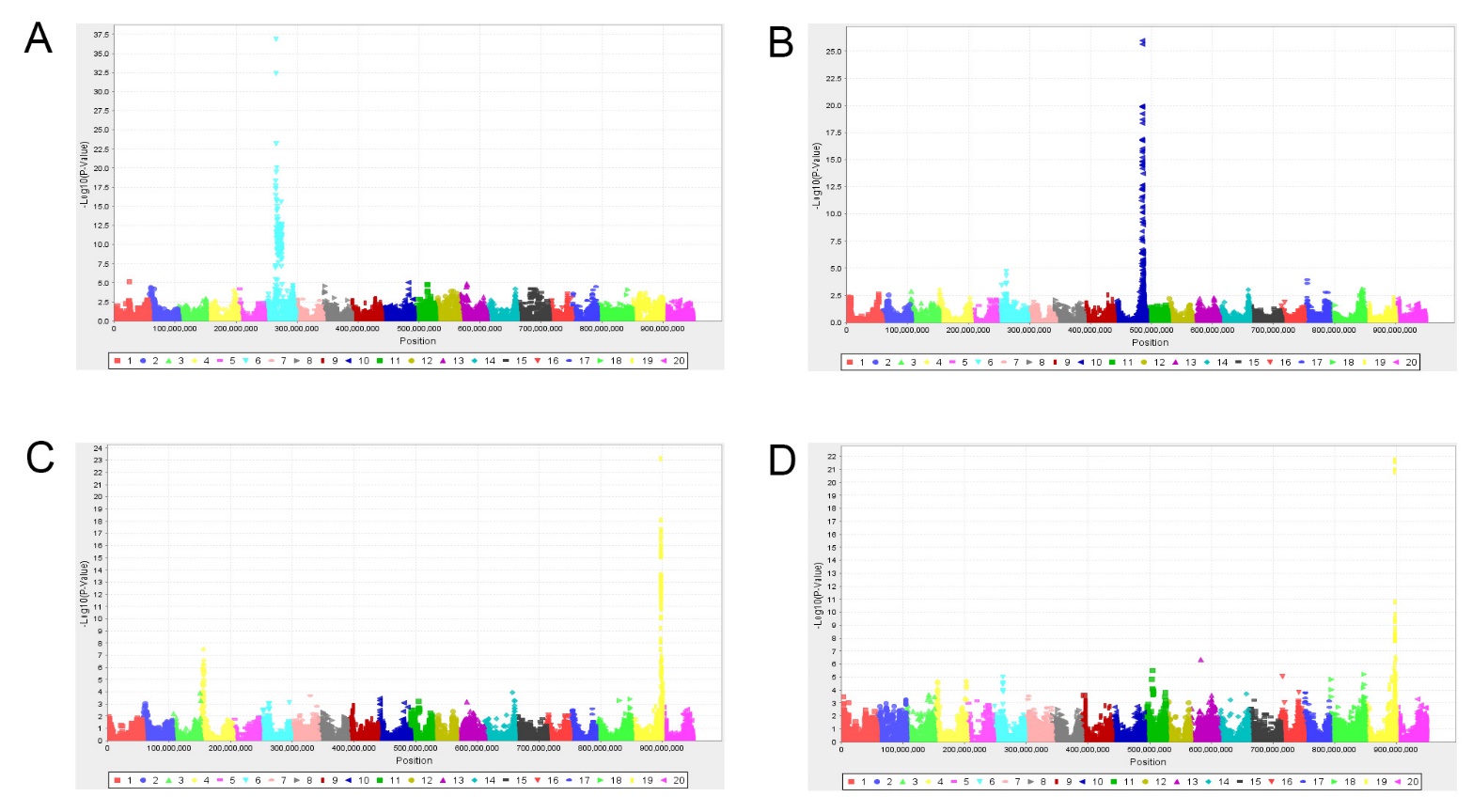
*

**Fig. S1.** Identification of associated SoySNP50K SNPs used to predict the allele status of *E1*, *E2*, and *E3* for *Glycine max* accessions from the USDA Soybean Germplasm Collection. The Manhattan plot *p*-values arranged by chromosome are shown for GWAS that used known *E1*, *E2*, and *E3* alleles as “phenotypes.” *(A)* *p*-values for *E1*. *(B)* *p*-values for *E2* *(C)* *p*-values for *E3-Ha* and *E3-Mi* alleles treated as one “phenotype” *(D)* *p*-values for *E3-Ha* and *E3-Mi* treated as individual “phenotypes”.
